# Impact on backpropagation of the spatial heterogeneity of sodium channel kinetics in the axon initial segment

**DOI:** 10.1371/journal.pcbi.1011846

**Published:** 2024-03-15

**Authors:** Benjamin S. M. Barlow, André Longtin, Béla Joós

**Affiliations:** 1 Department of Physics, University of Ottawa, STEM Complex, 150 Louis-Pasteur Pvt, Ottawa, Ontario, Canada; 2 Center for Neural Dynamics and AI, University of Ottawa, Ottawa, Ontario, Canada; 3 Department of Cellular and Molecular Medicine, Faculty of Medicine, University of Ottawa, Ottawa, Ontario, Canada; University of Pittsburgh, UNITED STATES

## Abstract

In a variety of neurons, action potentials (APs) initiate at the proximal axon, within a region called the axon initial segment (AIS), which has a high density of voltage-gated sodium channels (Na_V_s) on its membrane. In pyramidal neurons, the proximal AIS has been reported to exhibit a higher proportion of Na_V_s with gating properties that are “*right-shifted*” to more depolarized voltages, compared to the distal AIS. Further, recent experiments have revealed that as neurons develop, the spatial distribution of Na_V_ subtypes along the AIS can change substantially, suggesting that neurons tune their excitability by modifying said distribution. When neurons are stimulated axonally, computational modelling has shown that this spatial separation of gating properties in the AIS enhances the backpropagation of APs into the dendrites. In contrast, in the more natural scenario of somatic stimulation, our simulations show that the same distribution can impede backpropagation, suggesting that the choice of orthodromic versus antidromic stimulation can bias or even invert experimental findings regarding the role of Na_V_ subtypes in the AIS. We implemented a range of hypothetical Na_V_ distributions in the AIS of three multicompartmental pyramidal cell models and investigated the precise kinetic mechanisms underlying such effects, as the spatial distribution of Na_V_ subtypes is varied. With axonal stimulation, proximal Na_V_ *availability* dominates, such that concentrating *right-shifted* Na_V_s in the proximal AIS promotes backpropagation. However, with somatic stimulation, the models are insensitive to *availability* kinetics. Instead, the higher *activation* threshold of *right-shifted* Na_V_s in the AIS impedes backpropagation. Therefore, recently observed developmental changes to the spatial separation and relative proportions of Na_V_1.2 and Na_V_1.6 in the AIS differentially impact *activation* and *availability*. The observed effects on backpropagation, and potentially learning via its putative role in synaptic plasticity (e.g. through spike-timing-dependent plasticity), are opposite for orthodromic versus antidromic stimulation, which should inform hypotheses about the impact of the developmentally regulated subcellular localization of these Na_V_ subtypes.

## Author summary

Neurons use sodium ion currents, controlled by a neuron’s voltage, to trigger signals called action potentials (APs). These APs typically result from synaptic input from other neurons onto the dendrites and soma. An AP is generated at the axon initial segment (AIS) just beyond the soma. From there, it travels down the axon to other cells, but can also propagate “backwards” into the soma and dendrites. This “backpropagation” allows the neuron to compare the timing of outgoing and incoming signals at synapses where input was received, a feedback process that modifies its connections to other neurons (spike-timing-dependent synaptic plasticity) which is a mechanism for learning. It is puzzling that in many neurons, sodium ion channels come in two types: high-voltage threshold channels clustered near the soma where the AIS begins, and low-voltage ones further away towards the axon. This separation changes in the early development of the animal, which raises the question of its role in backpropagation. We constructed detailed mathematical models to explore how separation affects backpropagation. Separation either impedes or enhances backpropagation, depending on whether the AP results from input to the soma or dendrites or, less typically, input received in the axon. This is explained by the different effects the separation has on two key kinetic processes that govern sodium currents.

## Introduction

In fluorescence microscopy images of neurons, the axon initial segment (AIS) is visible as a patch of axonal membrane near the soma with a high density of voltage-gated ion channels. These channels enable the AIS to initiate and shape action potentials (spikes) and regulate neuronal excitability [1]. The AIS can be thought of as an organelle that lives within the first ≈100μm of axonal membrane and whose function it is to supply the current needed to initiate spikes when the neuron is poised to fire—usually in response to synaptic input. The AIS can move up and down the axon and also change its length on a timescale of hours to days. This phenomenon, called structural AIS plasticity, enables neurons to optimize their sensitivity to specific input frequencies during development and to homeostatically adjust their intrinsic excitability [2–4]. GABAergic input can also impinge on the AIS from axo-axonic synapses, such that the AIS can be modulated directly by interneurons. Synaptic input at the AIS can rapidly and precisely control the excitability of individual neurons for sound localization [5]. Fast AIS plasticity, including receptor-mediated changes to local ion channel properties and endocytosis of voltage-gated channels, occurs on timescales of seconds to minutes [6]. (This is distinct from pathological remodelling induced by ischemia, although in [7], it was recently demonstrated that cortical neurons are more robust to interruptions in blood flow than previously thought.) The outsized electrophysiological influence of the AIS demands robust characterization of this short piece of axon as it interacts with its environment.

Over three-quarters of all neurons in the mammalian cortex are pyramidal cells (see Fig A in S1 Text), which have dendrites spanning the thickness of the cortex (several mm) and AIS lengths on the order of tens of μm [8–11]. The AIS requires a high density of voltage-gated sodium channels (Na_V_s) to prime and initiate action potentials (APs) [12–14]. In pyramidal cells, the AIS features two Na_V_ subtypes, with an interesting spatial distribution: Na_V_1.2 channels cluster near the soma (i.e. at the proximal AIS) while Na_V_1.6 cluster toward the distal AIS [15–17]. However, the purpose of this separated distribution of Na_V_ subtypes remains unclear [18, 19]. Further, recent experiments have revealed that as neurons develop, the spatial distribution of Na_V_s in the AIS can change substantially, suggesting that neurons tune their excitability by modifying said distribution [20].

Our modelling study is motivated by the following question: What effect does the separated spatial distribution of Na_V_1.2 and Na_V_1.6 in the AIS have on excitability and backpropagation? And does the answer depend on whether stimulation is orthodromic or antidromic? In particular, how does the finding in [15], that the separated distribution of Na_V_ subtypes favours backpropagation—simulated with axonal (antidromic) current injection—generalize to the more common situation of somatic (orthodromic) stimulation?

It is a prevalent view that the Hodgkin-Huxley style kinetics of Na_V_1.2 are *right-shifted* relative to those of Na_V_1.6 by an amount $V_{\text{RS}}∼10-15\text{mV}$ [13–15, 17, 21, 22]. Due to their *right-shifted* gating properties (see Fig P in S1 Text), Na_V_1.2 channels are often described as “high-threshold” channels, since the *right-shift* increases their half-*activation* voltage, relative to Na_V_1.6. Because the same *right-shift* also increases Na_V_1.2 *availability*—i.e. it reduces the proportion of inactivated Na_V_1.2 channels at a given voltage, compared to Na_V_1.6—it is an oversimplification for the purposes of this study to call them high- and low-threshold channels, respectively. For this reason we instead say that Na_V_1.2 channels are *right-shifted*.

Interestingly, Na_V_1.6 invades the proximal AIS as pyramidal neurons mature [20]. To be meaningful, the statement of Hu et al. that concentrating Na_V_1.2 in the proximal AIS promotes backpropagation [15], requires that the *right-shifted* *gating properties* of Na_V_1.2 do the promoting: Suppose a given stimulus is just barely sufficient to evoke a backpropagating AP (BAP) in the neuron with Na_V_1.2 concentrated in the proximal AIS and Na_V_1.6 in the distal AIS. If the function of said channel distribution is to ensure backpropagation of the AP to the soma and dendrites (as stated in [15]), then backpropagation should fail with the same stimulus if the proximal AIS were instead populated with Na_V_1.6.

In [15], the rôle of Na_V_1.2 in promoting backpropagation is contingent upon simulations wherein the density of Na_V_1.2 was incrementally lowered in the AIS. However, at the proximal AIS, the active Na^+^ conductance was almost entirely composed of Na_V_1.2 channels. It does not follow then, that concentrating Na_V_1.2 in the proximal AIS promotes backpropagation, from the fact that removing *the only* Na_V_ channels in that area (which happen to be Na_V_1.2 at that developmental stage [20]) stopped backpropagation.

A more recent experimental paper which is the most directly relevant to [15] is Katz et al. (2018) [18], which compared AP thresholds in engineered mouse pyramidal neurons lacking Na_V_1.6 to wild-type neurons with Na_V_1.2 and Na_V_1.6 in the AIS. In [18], they downplay the importance of Na_V_ subtypes in determining the excitability differences seen in the proximal versus distal AIS. Whereas antidromic stimulation was used in [15], orthodromic stimulation (somatic current injection) was used in [18]. There were no data available that isolated the effect of orthodromic versus antidromic stimulation w.r.t. the role of Na_V_ subtypes in the AIS in setting the backpropagation threshold. Here our modelling shows that the stimulation site matters, and can invert the experimental conclusions, which should motivate a comparative experimental study.

The separated Na_V_ distribution is reported to promote backpropagation—which is important for learning—following axonal stimulation [15]. There is also evidence that mutations which alter the gating properties of Na_V_1.2 are involved in epilepsy and autism [23]. Backpropagated spikes drive learning by depolarizing the postsynaptic membrane, which triggers metabolic events that give rise to synaptic plasticity, including spike-timing-dependent plasticity [24]. There is experimental evidence that postsynaptic backpropagation can release retrograde messengers into the synapse, and influence the future release of neurotransmitters from the presynaptic neuron [25]. A backpropagating action potential can also underlie bursting in cortical neurons as it can return to the cell body from the dendrites as a depolarizing after-potential, which in turn can initiate another somatic AP [26, 27]. Bursting can also occur in layer 5 pyramidal cells following the generation of a dendritic BAP-activated Ca^2+^ spike (BAC spike), e.g. in the presence of synaptic input. The associated BAPs can further influence the dendritic dynamics [28–31].

Not all layer 5 pyramidal cells can generate dendritic spikes as the size of the apical dendritic tree varies [32]. Dendritic spikes have also been reported to vary across species, and are not common in human layer 5 pyramidal cells [33], owing partly to their enhanced dendritic compartmentalization [34]. Thus, to further understand the original reports that Na_V_ segregation promotes BAPs, we investigate how Na_V_ segregation in the AIS can decrease the BAP threshold (described below) using the model of [15] (itself based on [26]). This provides the backbone to study the basic effects on BAPs of the AIS excitability profile, under both somatic and axonal stimulation. For the sake of generality, we complement these results by considering a state-of-the-art model of layer 5 pyramidal cells with perisomatic BAPs and dendritic BAC firing [29], adapted to include a more realistic AIS and axon.

Other computational powers are attributed to the AIS. Moving the initiation site away from the soma (i.e. toward the distal AIS) beyond a critical distance enables high-frequency spiking in cortical neurons, increasing the maximum spike frequency by an order of magnitude [14]. Separating Na_V_1.6 into the distal AIS is said to push the initiation site toward that location, owing to those channels’ lower voltage threshold [15, 21]. However, in [35], simulations having only one Na_V_ type demonstrated that passive cable properties are sufficient to locate AP initiation at the distal AIS.

In our simulations, we alter the composition of the AIS and look for changes in the backpropagation threshold. We distribute *right-shifted* Na_V_ gating properties along the AIS by differentially distributing two functionally distinct classes of sodium channels, referred to here as Na_V_1.2 and Na_V_1.6 following [15, 21] and [20]. We systematically alter the Na_V_ distribution, by varying the extent to which Na_V_ subtypes are spatially segregated in the AIS without affecting the total Na_V_ density.

**Fig 1:**
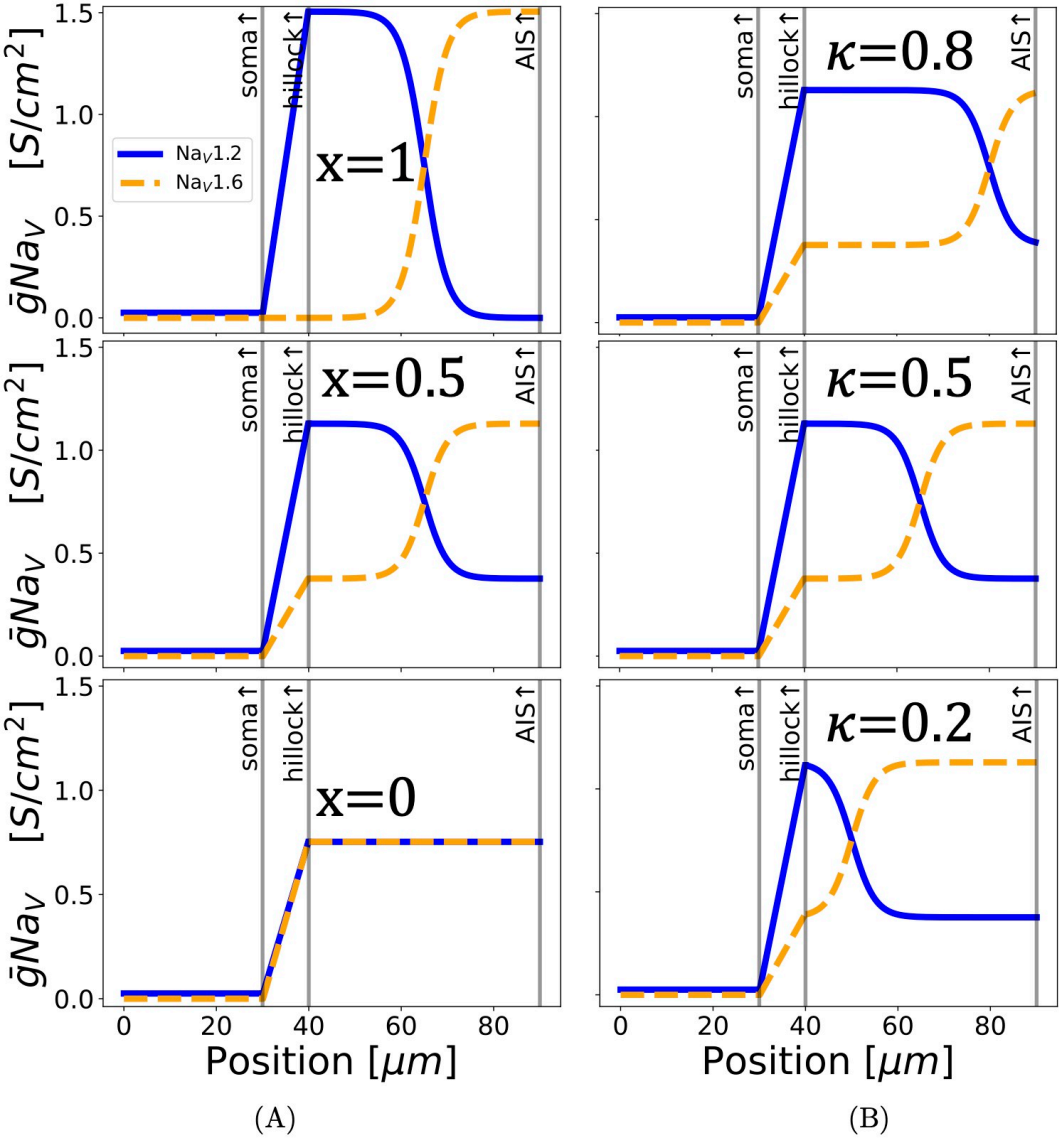
Modifying the spatial distribution of Na_V_ subtypes in the AIS while keeping the total conductance constant. (**A**) The spatial separation of Na_V_ subtypes in the AIS is varied using the parameter “*x*” with *κ* = 0.5. The top plot is a model setup with a separated distribution [15, 20] of Na_V_s in the AIS. The high threshold Na_V_1.2 (indicated in blue) are concentrated close to the soma, and the low threshold Na_V_1.6 (indicated in orange) are kept distal to the soma. Moving from top to bottom, both Na_V_ subtypes are distributed ever more evenly along the AIS. We chose the parameter name “*x*” to vary the spatial separation of the AIS Na_V_ distributions, because the separated distribution is *x*-shaped. Setting *x* = 1 in our simulations gives the separated distribution, and *x* = 0 gives the “flat” distribution wherein both Na_V_ subtypes are uniformly mixed. (**B**) Variation of the crossover location (*κ*) of Na_V_s in the AIS with *x* = 0.5. We have lengthened the AIS to 50μm in this graphic for visual clarity.

We compute the threshold for backpropagation as the amplitude of a brief current pulse that causes an AP to propagate back into the dendrites and cause a sufficient depolarization (Sections B and C in S1 Text). This is done in three biophysically detailed and independently tuned multicompartmental pyramidal cell models ([15, 29]), two of which are based on the Hu et al. (2009) model and involve the same morphology but with differing soma-dendrite excitability balance (cell geometries are provided in Figs A and B in S1 Text). This threshold is computed as a function of the spatial segregation of the Na_V_ subtypes in the AIS by continuously varying their density profiles from fully overlapping to strongly separated (Fig 1).

We show that Na_V_ separation reduces the backpropagation threshold with axonal stimulation but can impede backpropagation with somatic stimulation. This asymmetrical result was not expected. To explain our results, we independently modify the *right-shift* (*V*_RS_) of selected Na_V_1.2 gating variables and their respective time constants by an amount Δ*V*_RS_ (i.e. *V*_RS_ → *V*_RS_ + Δ*V*_RS_). These modifications to Na_V_1.2 gating are applied only in the AIS.

Sweeping Δ*V*_RS_ (while clamping other gating variables to nominal *V*_RS_ values) reveals that (I) Na_V_1.2 *availability* and its time constant explain how proximal Na_V_1.2 promotes backpropagation with axonal stimulation, and (II) the threshold of steady-state *activation* explains how Na_V_1.2 suppresses backpropagation and reduces excitability with somatic stimulation.

Being a feature of pyramidal cells, the plastic distribution of AIS Na_V_ subtypes that we model applies to something like eight out of ten cortical neurons [8]. Various experimental and computational techniques used to study the biophysical determinants of AIS excitability across the lifespan have involved different stimulation sites [36, 37]. Here we demonstrate opposing effects on backpropagation with orthodromic versus antidromic stimulation by altering the separated Na_V_ distribution. Both stimulation modes are used by experimentalists [11, 15, 18, 38], and certain pyramidal neurons are also known to receive axo-axonic input at the AIS as well as somatodendritic input [6]. It is thus important to know whether and how the spatial profile of Na_V_ channel subtypes really enhances backpropagation in vivo, and whether moving the stimulating electrode can bias or even invert experimental findings, as our work demonstrates. Apart from explaining the dynamical mechanism behind the dependence of AP generation on AIS Na_V_ distribution, we clearly show that the site of stimulation matters, a finding that is present robustly in different models and which merits experimental confirmation. Changes to AIS properties and the follow-on effects on backpropagation must affect the entire cortex.

## Results

### Hypothetical Na_V_ distributions in the AIS

We begin with our implementation of the model from Hu et al. (2009) [15] (Hu-based model), using their morphology, K_V_ and Na_V_ kinetics (for details, see Materials and methods). The standard AIS length in our model is 25 μm, based on measurements from [10]. A key feature of the Na_V_ distribution that changes during development, is the extent to which the voltage-gated sodium channel subtypes Na_V_1.2 and Na_V_1.6 are localized in the proximal and distal AIS, respectively [20]. In our simulations, the relative proportion of Na_V_1.2 versus Na_V_1.6 at a given position along the AIS can be changed without affecting the total Na_V_ density at any point (Eq 6).

Fig 1 shows how the parameters *x* and *κ* control the way Na_V_ subtypes are spread out along the AIS. When *x* is at its highest value of 1, the subtypes Na_V_1.2 and Na_V_1.6 are spaced apart from each other, with Na_V_1.2 concentrated in the proximal AIS and Na_V_1.6 in the distal AIS, approximating the distribution observed in developing pyramidal neurons (see [20]). Decreasing *x* transforms this separated distribution into a uniform mix (*x* → 0) where Na_V_1.2 and Na_V_1.6 are distributed homogeneously. This can be seen in Fig 1A.

Every distribution except the uniform Na_V_ mix has a location along the AIS at which the density of Na_V_1.6 overtakes the Na_V_1.2 density. That location, which we call the Na_V_ crossover and denote *κ*, is also varied in our simulations (see Fig 1A; *κ* is a dimensionless length normalized by the AIS length).

To cement our results, we will further apply identical transformations to the Na_V_ distribution in a cell having a ‘backward’ AIS, that is, with *distal* Na_V_1.2 and *proximal* Na_V_1.6. The results from the backward AIS model are nearly a mirror image of our findings.

For each hypothetical Na_V_ distribution, a short current pulse (1ms) is injected at a specific site, and the minimum (i.e. threshold) pulse amplitude *I* (in nA) required to elicit a spike is determined. Brief pulse durations separate the stimulation waveform from the intrinsic response of the cell. We define excitability in terms of two thresholds: backpropagation threshold *I*_*BP*_ (AP leading to a spike in the distal dendrites) and forward-propagation threshold *I*_*FP*_ (axonal AP threshold, recorded without regard to the amplitude of the somatodendritic depolarization).

Current is injected either in the middle of the soma (somatic stimulation) or the axon just distal to the AIS (axonal stimulation). In both cases, forward propagation refers to an AP travelling down the axon, and backpropagation always refers to an AP visible as a spike in the dendrites. Backpropagation was deemed to have occurred if all apical dendritic tips exceeded -63.0mV (i.e. a depolarization of 7.0mV above *V*_rest_) following stimulation (see Fig C in S1 Text).

In the following sections, we implement the above Na_V_ distributions in the Hu-based model [15] (Fig A in S1 Text), which we chose as a starting point because of its seminal role in the study of how Na_V_ subtypes in the AIS affect backpropagation. At *V*_rest_, the *activation* of Na_V_1.2 and Na_V_1.6 is negligible, so the total conductance at rest is not affected by *x* or *κ* (see Fig Pi in S1 Text).

Due to the “curse of dimensionality” and limitations in the spatial resolution of experimental measurements, many parameters in multicompartmental models—such as the density of ion channels at every point on the cell membrane—must be estimated and require tuning, introducing subjective judgement on the part of the modeller (reviewed in [39]). Hu et al. [15] based their simulations on a hand-tuned model from [26]. The adjustment and re-adjustment of hand-tuned models is a potentially endless cycle [39]. It is desirable to have a model which is tuned automatically via an objective procedure, to break the loop of hand-tuning. To this end, in Generalization to Hay-based model and modified Hu-based model, we repeat the procedure described above—i.e. varying the Na_V_ distributions in the AIS as in Fig 1—in the model of Hay et al. (2011) [29] (Fig B in S1 Text). Hay et al. used an evolutionary algorithm to optimize the densities of nine simulated ion channels in each compartment of reconstructed layer-5b pyramidal neurons. The ground truth in that fitting consisted of somatodendritic spiking patterns recorded in a variety of such neurons from adult rats.

The third model, a modification of our Hu-based model with significant qualitative differences in its backpropagating action potential, is included in Section D in S1 Text. In the latter model, the dendritic excitability is much higher, with negligible attenuation of the backpropagating action potential. The dendritic Na_V_ density is increased 10-fold relative to the Hu-based model in the main text, and the somatic Na_V_ density is decreased 3-fold. Multiple models with differing conductances, biophysics, and morphology demonstrate the robustness of our results, as we can modify the density profiles of Na_V_1.2 and Na_V_1.6 in the AIS without affecting any other compartments. For further details, see Materials and methods and S1 Text.

### Somatic stimulation

**Fig 2:**
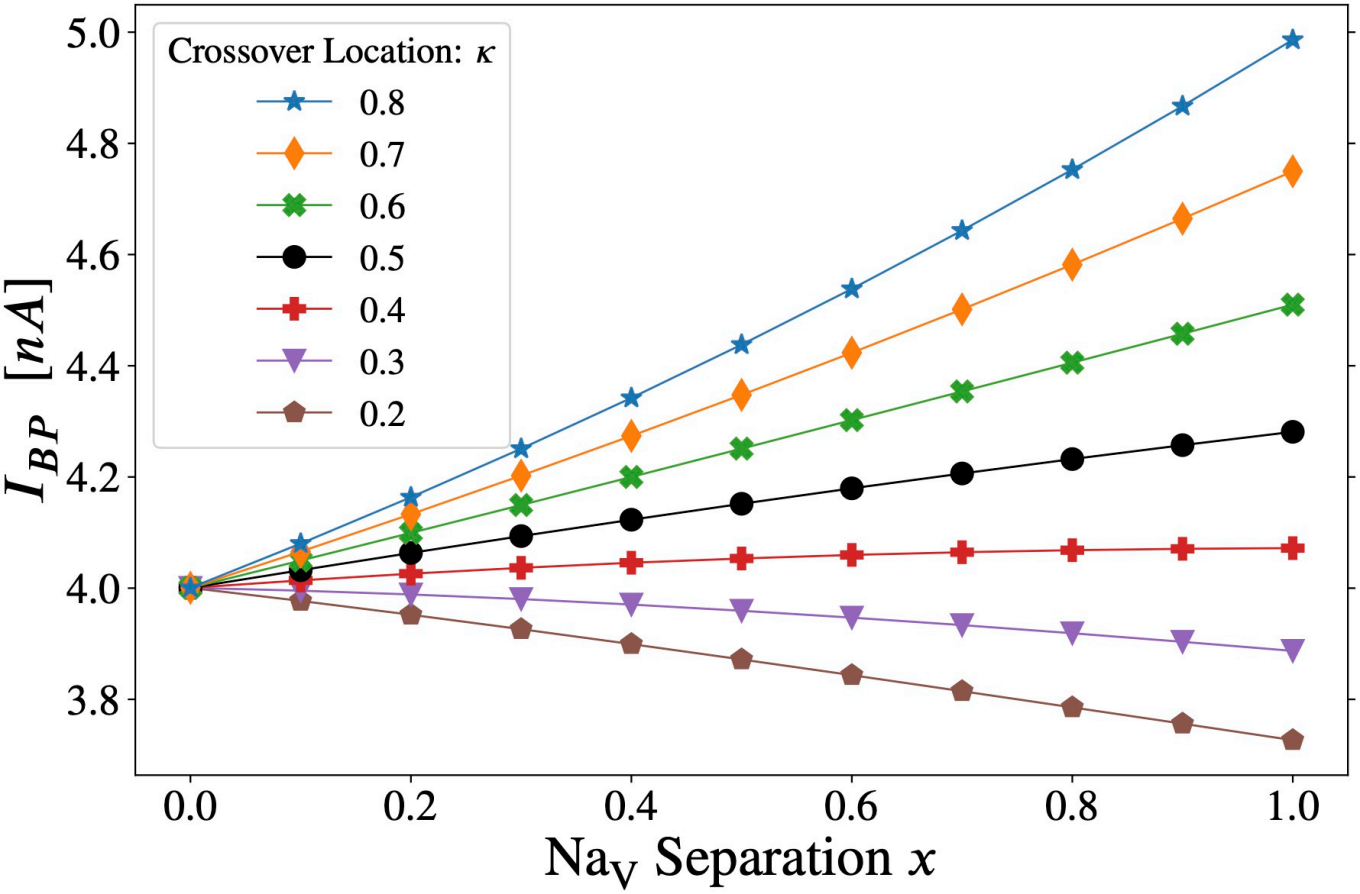
Somatic stimulation: Combined effect of varying crossover location (*κ*) and Na_V_ separation (*x*) in the axon initial segment. The threshold for forward AP propagation is the same as for backpropagation. Varying the separation parameter “*x*” from *x* = 0 to *x* = 1, the distribution of Na_V_ channels goes from flat (homogeneous) to separated, the latter approximating the distribution observed in developing pyramidal neurons (see Fig 1A). Note that curves for all values of *κ* converge to a single point at *x* = 0, since *κ* can have no effect when the two Na_V_ subtypes are uniformly distributed along the AIS. The lines have been drawn to guide the eye.

In Fig 2, both negatively and positively sloped backpropagation threshold curves with respect to *x* are present, indicating that Na_V_ separation can promote or impede backpropagation (respectively). Changes in threshold can be as large as 30%. Moving the Na_V_ crossover (*κ*) toward the distal AIS shifts the backpropagation threshold curves upward. A qualitative change, namely the sign of the slope, occurs around *κ* = 0.4.

An intuitive explanation for this latter effect is that moving the crossover location away from the soma causes the AIS to be dominated by Na_V_1.2 channels (see Fig 1B, *κ* = 0.8), which have a higher *activation* voltage threshold than Na_V_1.6. APs still initiate in the distal AIS, but the dominant Na_V_1.2 renders the cell less excitable. Further, for *κ* ≳ 0.4, the backpropagation threshold increases as we tend toward the separated, *x*-shaped distribution of Na_V_s. This behaviour is the opposite of what is observed for axonal stimulation below and in [15]. We repeated these simulations with AIS length up to 100μm (Fig L in S1 Text) and also with stimulation at the main apical dendrite (Fig N in S1 Text) instead of the soma, and obtained the same qualitative results as Fig 2 (see Section D.1 in S1 Text).

The negatively sloped curves do not necessarily imply that proximal Na_V_1.2 promotes backpropagation in the case of somatic stimulation. In those curves (*κ* ≲ 0.4), the AIS is mainly populated with Na_V_1.6 when *x* > 0. Also note that decreasing *κ* places more Na_V_1.6 channels nearer to the soma (see Fig 1B, *κ* = 0.2). In that case, the threshold-lowering effect of Na_V_ separation could come from the increased total Na_V_1.6 density that results from increasing *x* when *κ* is relatively small, rather than from the proximal accumulation of Na_V_1.2 with increasing *x*. Further, increasing *κ* (which increases the ratio of Na_V_1.2 to Na_V_1.6 in the AIS) raises the threshold for all curves in Fig 2 (see also Fig M in S1 Text). It is then consistent to postulate that for somatic stimulation, the backpropagation threshold is increased by AIS Na_V_1.2 at all values of *x* and *κ*, and Fig 2 is consistent with AIS Na_V_1.6 enhancing excitability and backpropagation. In other words, for **somatic stimulation**:

- when *κ* < 0.5 and *x* > 0, the AIS is dominated by Na_V_1.6: increasing *x* decreases the proportion of total AIS Na_V_ conductance due to Na_V_1.2 (negative slope: separated distribution yields the lowest backpropagation threshold).
- when *κ* > 0.5 and *x* > 0, the AIS is dominated by Na_V_1.2: increasing *x* increases the proportion of total AIS Na_V_ conductance due to Na_V_1.2 (positive slope: separated distribution yields the highest backpropagation threshold).

This effect is shown in Fig R in S1 Text. Although the above description is an appealing simplification, the impact of the spatial separation of Na_V_ subtypes (*x*) remains important, even when the AIS has equal amounts of Na_V_1.2 and Na_V_1.6. In other words, the combined effect of *x* and *κ* cannot be reduced to the resulting ratio of total Na_V_1.2 versus Na_V_1.6.

Lengthening the hillock with *κ* fixed also moves the crossover away from the soma. Curves with negative slope in Fig 2 became positively sloped when the hillock was lengthened from 10μm to 30μm (Fig O in S1 Text). The forward propagation threshold for somatic stimulation with a single 1ms current pulse is not included in a separate figure since it is identical to the backpropagation threshold in this model. This does not depend on the somatic injection site. The effect of Na_V_ gating properties in the AIS on backpropagation threshold is examined systematically in Modifying the *right-shift* of Na_V_1.2 gating properties in the AIS.

**Fig 3:**
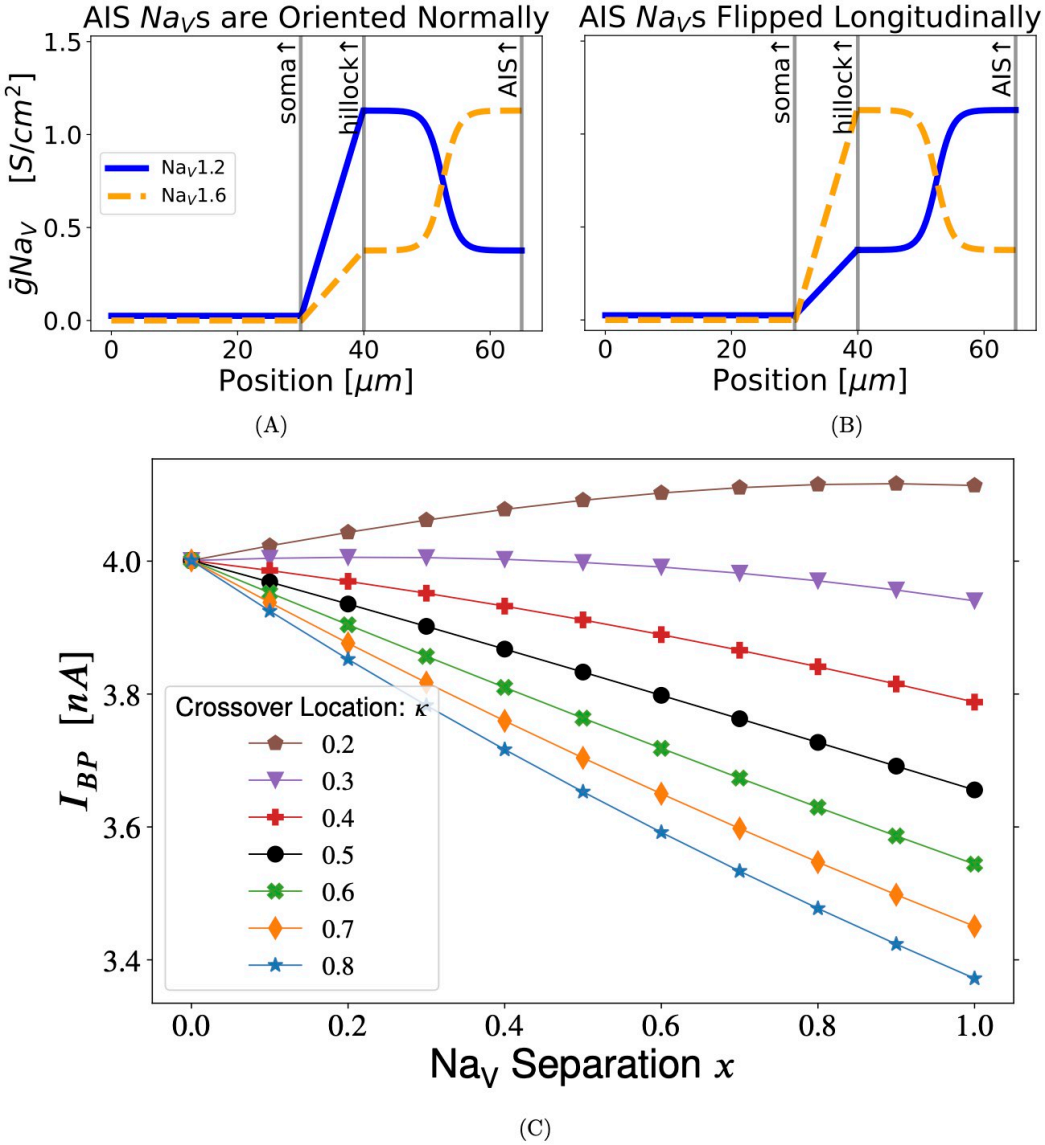
Somatic stimulation with a flipped Na_V_ distribution: Backward AIS. When the AIS Na_V_ distribution is flipped proximal-to-distal, setting *x* = 1 concentrates Na_V_1.6 at the proximal AIS and Na_V_1.2 at the distal AIS—the opposite of what is observed in many pyramidal cells [15–17]. **(A)** AIS with proper longitudinal placement of Na_V_s. **(B)** AIS with a longitudinally flipped Na_V_ distribution. In both plots, *x* = 0.5 and *κ* = 0.5. **(C)** Somatic stimulation with AIS Na_V_s flipped as in **(B)**: This result is close to a mirror image of Fig 2. The lines have been drawn to guide the eye.

An informative variation on Fig 2 is shown in Fig 3C in which the AIS is “put on backward”, such that Na_V_1.2 is concentrated in the distal AIS and Na_V_1.6 is proximal to the soma. As one might expect, the effect of varying *x* and *κ* in Fig 3 is opposite to what is seen in Fig 2, albeit with some new curvature at low *κ*. This reinforces the observation that proximal Na_V_1.6 facilitates backpropagation with somatic stimulation.

### Axonal stimulation

**Fig 4:**
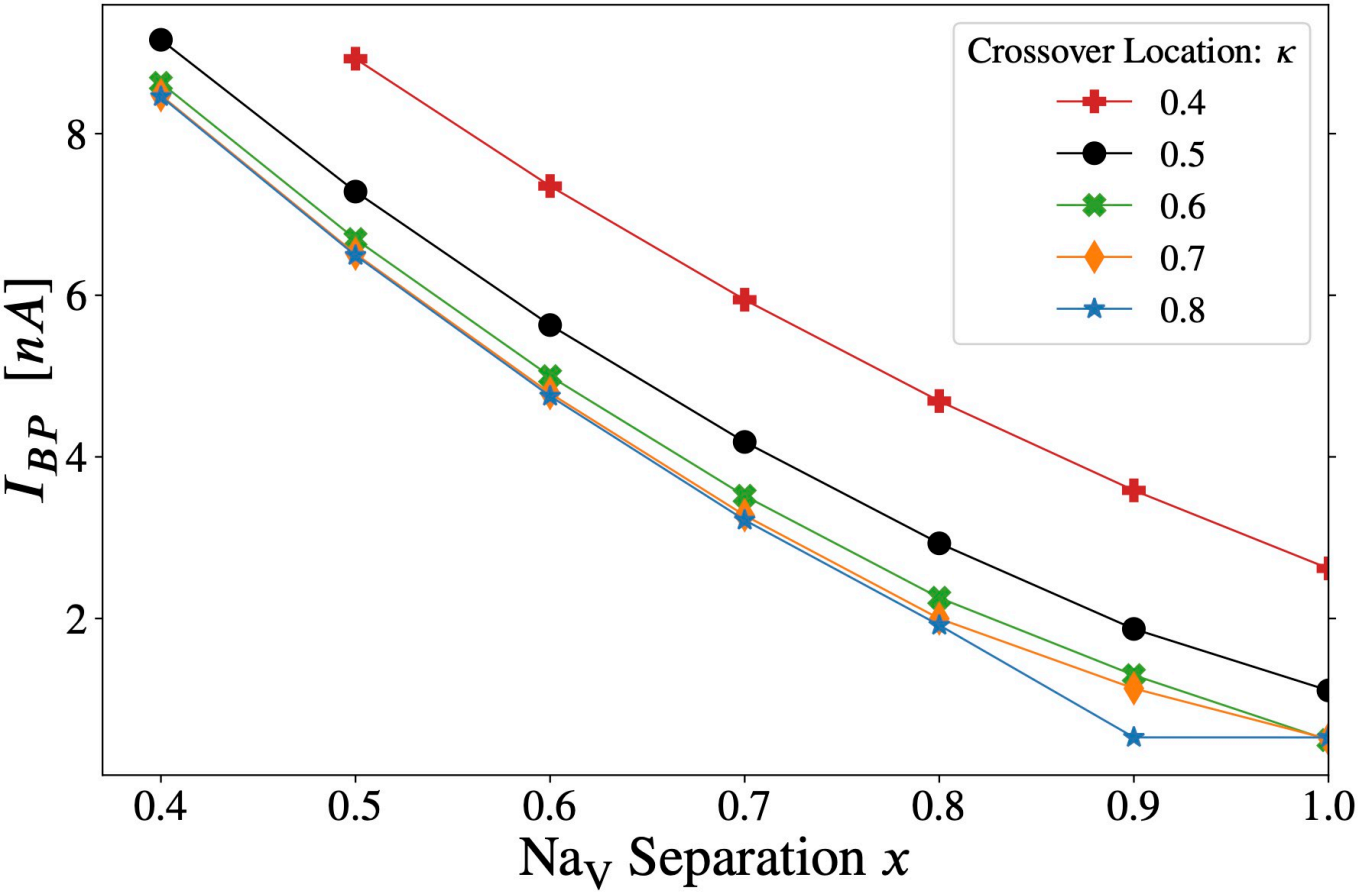
Axonal stimulation: Effect of varying crossover location (*κ*) and Na_V_ separation (*x*) in the AIS on the backpropagation threshold (see Fig 1). When computing the threshold, the stimulating current was limited to a maximum of 10nA, to prevent unphysiological local depolarization at the stimulation site. Due to the smaller diameter of the axon (relative to the soma), 10nA is sufficient to depolarize the membrane potential to ≈+80mV at the stimulation site, whereas the resting potential is $V_{\text{rest}}=-70\text{mV}$. To achieve backpropagation within that constraint (following axonal stimulation), our model required some amount of proximal Na_V_1.2, delivered through the combined effects of Na_V_ separation (*x* ≳ 0.5) and a sufficiently distal crossover position *κ* ≳ 0.4. Separating the two Na_V_ subtypes (*x* → 1) lowers the threshold, in agreement with the finding in [15] that proximal accumulation of Na_V_1.2 promotes backpropagation, albeit due to different gating properties (Fig 6B). Increasing *κ* raises the proportion of Na_V_1.2 (relative to Na_V_1.6) in the AIS and lowers the backpropagation threshold as well. Threshold changes here are larger than for somatic stimulation (Fig 2). The lines have been drawn to guide the eye.

With axonal stimulation (current injection just distal to the AIS), Na_V_ separation consistently lowers the backpropagation threshold (Fig 4). Contrary to somatic stimulation (Fig 2), moving the Na_V_ crossover (*κ*) toward the distal AIS shifts the backpropagation threshold curves downward.

The decreasing threshold with respect to *x* in Fig 4 is consistent with the conclusion from [15], which used axonal stimulation, that proximal Na_V_1.2 in the AIS promotes backpropagation. Our results for *κ*, with axonal stimulation, provide new support for their findings.

This agreement is interesting because our method of modifying the AIS Na_V_ distribution (described above in Fig 1) is quite different from their simulations. Our transformations deliberately preserve the total Na_V_ density at every AIS segment—if Na_V_1.2 is removed, Na_V_1.6 must take its place. Conversely, in [15], the density profile of Na_V_1.2 is scaled by a constant factor everywhere in the AIS, leaving the Na_V_1.6 profile intact. We denote the scaling factor

$$\alpha_{{\text{Na}}_{\text{V}}1.2}⩾0.$$ (1)

That is, if the Na_V_1.2 density profile is scaled down in [15], nothing is added to compensate for the missing channels. Under the latter transformation, we expect that $\alpha_{{\text{Na}}_{\text{V}}1.2}>1$ would lower *I*_*BP*_ and $\alpha_{{\text{Na}}_{\text{V}}1.2}<1$ would raise *I*_*BP*_ in our models as well, since scaling the density profile of Na_V_1.2 in a separated distribution with a specified *κ* and *x* > 0 would scale the total AIS Na_V_ conductance, especially at the proximal AIS. We have reproduced this procedure in the Hay model, see Rescaling the Na_V_1.2 density profile by a uniform factor in the AIS.

It is one thing to say that reducing (increasing) the total density of voltage-gated sodium channels in the proximal AIS, which happen to be Na_V_1.2 channels, will raise (lower) the backpropagation threshold (respectively). But since we preserved the local Na_V_ density in our results (above), the changes to *I*_*BP*_ can only be a manifestation of the spatial heterogeneity of sodium channel *gating properties*. Since *right-shift* is the most important feature distinguishing Na_V_1.2 from Na_V_1.6 in this model, we included a sensitivity analysis, see Modifying the *right-shift* of Na_V_1.2 gating properties in the AIS. The analysis in that section explains how the proximal accumulation of Na_V_1.2 is able to simultaneously lower *I*_*BP*_ with axonal stimulation (Fig 4) and raise *I*_*BP*_ with somatic stimulation (Fig 2).

### Forward propagation threshold

**Fig 5:**
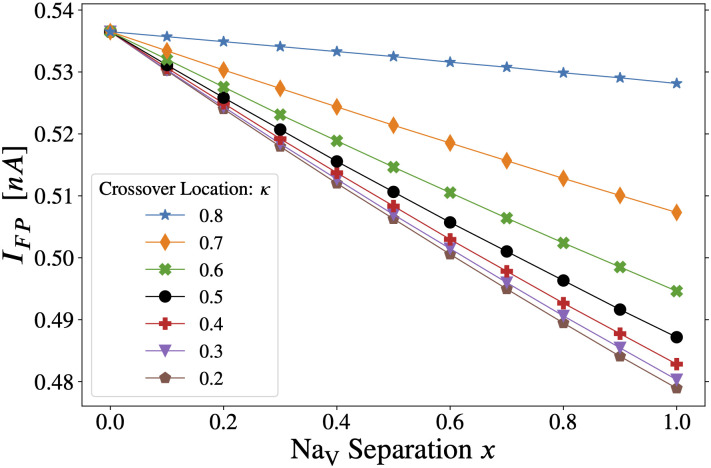
Axonal stimulation: Effect of *x* and *κ* on forward propagation threshold. The trend for all constant *κ* curves is that raising the proportion of total AIS Na_V_1.6 (by reducing *κ*) or concentrating Na_V_1.6 in the distal AIS (by increasing *x*) lowers the threshold to initiate forward propagating action potentials (see Fig 1). Note that although this threshold current pulse is not sufficient to satisfy our strict backpropagation criterion (see Section B in S1 Text), the most distal apical dendrites will be depolarized by several mV relative to their local resting potential (see Fig Dii in S1 Text). The lines have been drawn to guide the eye.

The forward-propagation threshold *I*_*FP*_, also referred to as the AP threshold, is shown in Fig 5 for the Hu-based model. With axonal stimulation only, it is possible to elicit an action potential without creating sufficient depolarization in the apical dendrites to meet our strict criterion for backpropagation (see Figs D and C in S1 Text). Note, however, that the most distal dendrites depolarize to several mV above their local resting potential (see Fig Dii in S1 Text). Stimulation amplitude is an order of magnitude lower than in the case of *I*_*BP*_. This is expected with axonal stimulation due to the high Na_V_ density of the distal AIS, its electrical isolation from the soma, its proximity to the stimulus, and our stringent definition of *I*_*BP*_ (Section B in S1 Text). (Antidromically stimulated axonal APs that do not trigger a somatodendritic BAP have been observed in several neuron types [36, 37].) Further, as discussed in [39], Hu et al. [15] built their model on [26], in which somatic invasion of the axonal action potential is reduced.

As with *I*_*BP*_, increasing *x* lowers *I*_*FP*_. Na_V_ separation concentrates Na_V_1.6 in the distal AIS, making it more excitable in the portion nearer to the stimulation site. This finding is consistent with [15], who found that distal Na_V_1.6 density places the lowest initiation threshold (and therefore the AP trigger zone) at the distal AIS. However, [35] has shown that cable properties are sufficient to explain why the trigger zone is located at the distal AIS (see Discussion). Moving the crossover distally (*κ* → 1) increases the total proportion of Na_V_1.2 in the AIS and thereby raises the *I*_*FP*_ threshold due to *activation* *right-shift*.

### Modifying the *right-shift* of Na_V_1.2 gating properties in the AIS

Our results from varying the Na_V_ distribution may be counterintuitive. With axonal stimulation, concentrating low-threshold (i.e. *left-shifted*) Na_V_1.6 channels at the distal AIS ought to promote forward propagation (and it does, see Fig 5), but why would concentrating the high-threshold (i.e. *right-shifted*) Na_V_1.2 channels at the proximal AIS promote [15] backpropagation (see Fig 4)? And how does the asymmetry come about, such that separating Na_V_ subtypes can raise the backpropagation threshold with somatic stimulation, but always lowers it with axonal stimulation?

**Fig 6:**
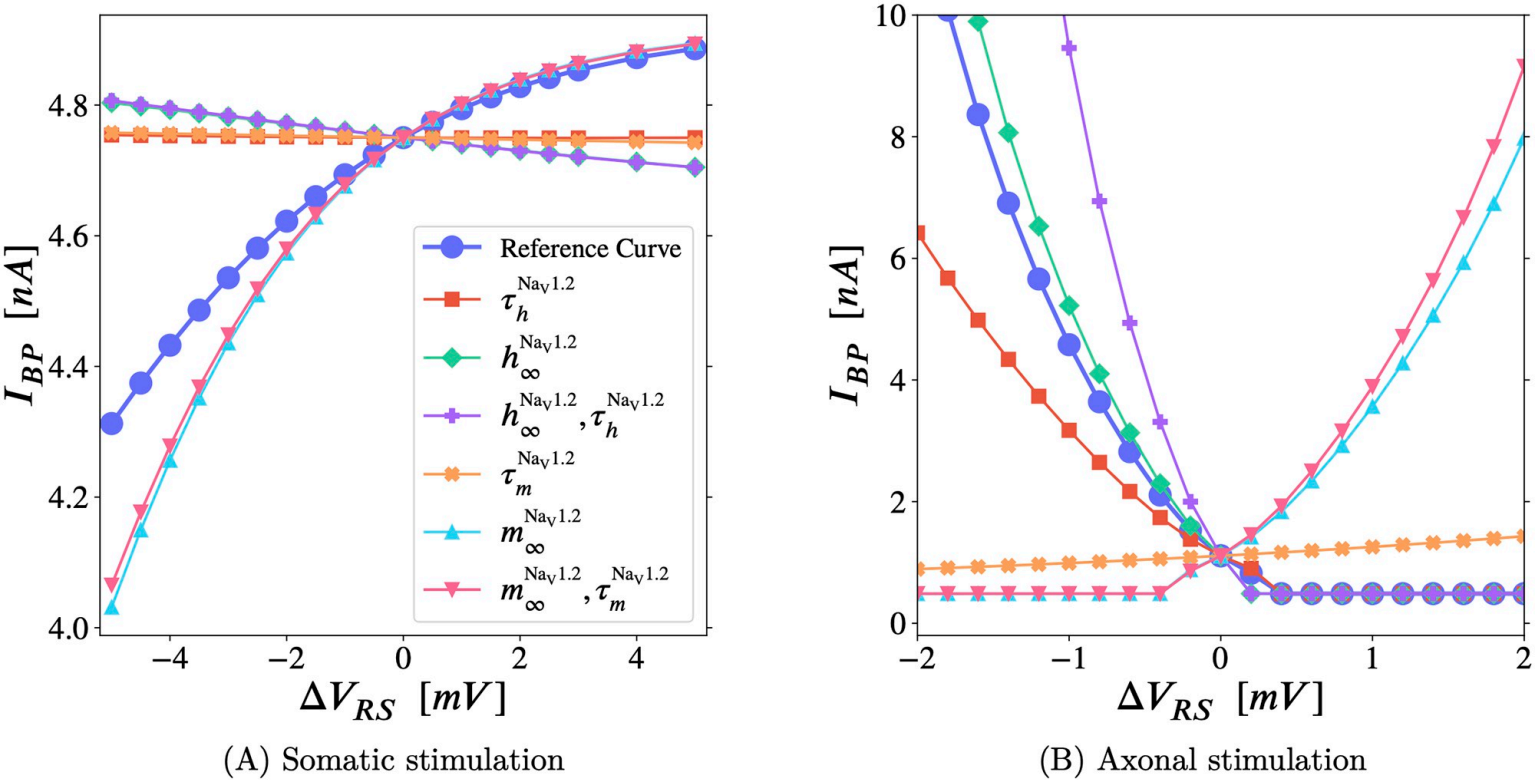
Sensitivity analysis of the backpropagation threshold to the *right-shift* of Na_V_1.2 gating properties. Along each curve, the gating properties named in the legend have their *right-shift* changed from *V*_RS_ to (*V*_RS_ + Δ*V*_RS_), and all the others are left unchanged (full definition and notation in Section F.2 in S1 Text). When Δ*V*_RS_ = 0, the *right-shift* is the reference value (or ‘nominal value’) of 13.0mV used for Na_V_1.2 in our simulations—see Section F.2 in S1 Text, *V*_RS_ indicated by small “→” in Fig P in S1 Text—around which we are performing this sensitivity analysis. The reference curve (legended ●) shows the net effect of *right-shifting* all Na_V_1.2 properties on *I*_*BP*_, via its slope. (It may be useful to imagine points on the reference curve as being pulled toward all the other curves that only change one property. The reference curve would then be the result of the combined pulls of those curves.) For each mode of stimulation, we identify the key gating properties through which *right-shift* controls backpropagation, by comparing the single property curves ($▲m_{\infty }^{{\text{Na}}_{\text{V}}1.2}$, $✖\tau_{m}^{{\text{Na}}_{\text{V}}1.2}$, etc.) to the reference curve (●). **(A) Somatic stimulation**: The reference curve has a positive slope (*right-shift* raises *I*_*BP*_), and it follows curves legended with $m_{\infty }^{{\text{Na}}_{\text{V}}1.2}$ near the nominal point (i.e. near Δ*V*_RS_ = 0). Hence, *I*_*BP*_ is governed by Na_V_ steady-state *activation* and is insensitive to the *right-shift* of all Na_V_ time constants. **(B) Axonal stimulation**: The reference curve has a negative slope (*right-shift* lowers *I*_*BP*_, i.e. promotes backpropagation), and it follows curves legended with $h_{\infty }^{{\text{Na}}_{\text{V}}1.2}$ or $\tau_{h}^{{\text{Na}}_{\text{V}}1.2}$. *I*_*BP*_ is then governed by proximal Na_V_ *availability*, owing to the *right-shift* of Na_V_1.2. Notably, with axonal stimulation, *I*_*BP*_ is also sensitive to the *right-shift* of $\tau_{h}^{{\text{Na}}_{\text{V}}1.2}(V)$, the—voltage-sensitive—*availability* time constant. Results are summarized in Table 1. The lines have been drawn to guide the eye. In both plots, *x* = 1.0. On the left *κ* = 0.7(to increase the slope, see Fig 2), and on the right, *κ* = 0.5.

In this section, we perform a type of sensitivity analysis with respect to the effects of the *right-shifted* Na_V_1.2 subtype. Fig 6 allows us to isolate the effects of *activation* *right-shift* versus *availability* *right-shift* on the backpropagation threshold.

Na_V_ subtypes are defined by their gating properties. Each Na_V_ distribution (Fig 1) produces a corresponding spatial profile of gating properties, including a profile of *right-shift*. (Gating properties are detailed in Section F in S1 Text, see Fig P in S1 Text.) In Fig 6, the AIS Na_V_s’ separation is fixed at *x* = 1. This spatial separation of Na_V_s concentrates *right-shift* in the proximal AIS, by concentrating Na_V_1.2 in that region (see Fig 1).

#### Modifying the *right-shift* of all gating properties of Na_V_1.2 in the AIS

Our model sets the nominal *right-shift* of Na_V_1.2 at $V_{\text{RS}}=13.0\text{mV}$ for compatibility with [15]. We use the parameter Δ*V*_RS_ to alter, in the AIS only, the *right-shift* of specific Na_V_1.2 gating properties. (For additional details, see Sections F.2 and F.3 in S1 Text.)

When a gating property (e.g. $m_{\infty }^{{\text{Na}}_{\text{V}}1.2}$) appears next to a curve in the legend of Fig 6, the voltage-dependence of said property is displaced by Δ*V*_RS_ (see, e.g., Eq 3). Likewise, if a gating property is *not* displayed in the legend, its voltage-dependence *isn’t* affected by Δ*V*_RS_.

For conceptual clarity, we call the variable *h*—which models the process by which Na_V_ channels are inactivated (or blocked) in Hodgkin-Huxley style kinetics [40]—the *availability*. For example, since the Na_V_1.2 conductance is given by $g_{{\text{Na}}_{\text{V}}1.2}\left(V,t\right)={\bar{g}}_{{\text{Na}}_{\text{V}}1.2}*{\left(m^{{\text{Na}}_{\text{V}}1.2}\right)}^{3}h^{{\text{Na}}_{\text{V}}1.2}$—where ${\bar{g}}_{{\text{Na}}_{\text{V}}1.2}$ is the maximal conductance and $m^{{\text{Na}}_{\text{V}}1.2}$ is the *activation*—$h^{{\text{Na}}_{\text{V}}1.2}$ is the proportion of those channels which are *not* inactivated. That is, $h^{{\text{Na}}_{\text{V}}1.2}(V,t)$ is the probability that a Na_V_1.2 channel selected at random is *available* to conduct sodium current. The inactivation is actually given by (1 − *h*) since, in such models, all channels are inactivated when *h* = 0 and all are available when *h* = 1.

Note that the *activation* $m^{{\text{Na}}_{\text{V}}1.2}(V,t)$ and *availability* $h^{{\text{Na}}_{\text{V}}1.2}(V,t)$ of real Na_V_1.2 channels are *right-shifted* by similar amounts when compared to the corresponding gating properties of Na_V_1.6 [13, 15, 17, 21, 22]. (Although certain receptors can temporarily *right-shift* *activation* without shifting steady-state *availability* [41].) In our simulations, we can simultaneously decrease (or increase) the *right-shift* of $m^{{\text{Na}}_{\text{V}}1.2}$ and $h^{{\text{Na}}_{\text{V}}1.2}$ together, making the Na_V_1.2 channels more (or less) similar to the Na_V_1.6 channels in the AIS (respectively). This produces the ‘reference curves’ legended ‘●’ in Fig 6A and 6B. In those curves, Δ*V*_RS_ shifts the voltage-dependence of every Na_V_1.2 gating property.

That is, as functions of membrane potential, the steady states and time constants in the curves marked ‘●’ are defined

$$●\leftarrow \{\begin{array}{l} m_{\infty }^{{\text{Na}}_{\text{V}}1.2}=m_{\infty }^{{\text{Na}}_{\text{V}}1.2}\left(V-\Delta V_{\text{RS}}\right)\text{,}\\ \tau_{m}^{{\text{Na}}_{\text{V}}1.2}=\tau_{m}^{{\text{Na}}_{\text{V}}1.2}\left(V-\Delta V_{\text{RS}}\right)\text{,}\\ h_{\infty }^{{\text{Na}}_{\text{V}}1.2}=h_{\infty }^{{\text{Na}}_{\text{V}}1.2}\left(V-\Delta V_{\text{RS}}\right)\text{,}\\ \tau_{h}^{{\text{Na}}_{\text{V}}1.2}=\tau_{h}^{{\text{Na}}_{\text{V}}1.2}\left(V-\Delta V_{\text{RS}}\right)\text{,} \end{array}$$ (2)

in the AIS.

Fig 6 connects local gating properties in the AIS, and their influence on the backpropagation threshold under somatic and axonal stimulation, to the effects of altering the Na_V_ distribution (seen above in Figs 2 and 4, respectively). For example, making Δ*V*_RS_ negative will *left-shift* the voltage-gated sodium current in the proximal AIS, which is analogous to adding more proximal Na_V_1.6. However, this is merely an analogy: With *x* = 1 and *κ* ≳ 0.8, the proximal AIS has only Na_V_1.2 channels, and positive values of Δ*V*_RS_ will *right-shift* the sodium current in that area beyond what is attainable by changing the local mix of Na_V_ subtypes.

The reference curve (●) in Fig 6A shows that *right-shifting* Na_V_s in the AIS *increases* *I*_*BP*_ for somatic stimulation. And the reference curve in Fig 6B confirms that proximal *right-shift* from Na_V_1.2 *lowers* *I*_*BP*_ for axonal stimulation.

#### Modifying the *right-shift* of selected gating properties of Na_V_1.2 in the AIS

Since Δ*V*_RS_ only affects Na_V_1.2 channels *within the AIS* (example provided in Fig Qii in S1 Text), we can determine which *right-shifted* gating properties drive the changes to *I*_*BP*_ that occur when the Na_V_ distribution is altered. To make said observation, in Fig 6 we also “shift-clamp” selected gating properties: We ignore the experimental fact that Na_V_ *activation* and *availability* tend to *right-shift* in unison [17, 21, 22, 42, 43], and that the steady-state of a gating variable and its voltage-sensitive time constant *right-shift* together as well [40]. Rather, we isolate the effects of individual Na_V_1.2 gating properties in our simulations by shifting some of them while leaving others alone.

We can apply Δ*V*_RS_ to Na_V_1.2 *availability* $h^{{\text{Na}}_{\text{V}}1.2}(V,t)$ without affecting the voltage dependence of the same channels’ *activation* $m^{{\text{Na}}_{\text{V}}1.2}(V,t)$ in our model. This is done via the steady-state *availability* $h_{\infty }^{{\text{Na}}_{\text{V}}1.2}\left(V-\Delta V_{\text{RS}}\right)$ and its voltage-dependent time constant $\tau_{h}^{{\text{Na}}_{\text{V}}1.2}\left(V-\Delta V_{\text{RS}}\right)$, while leaving the corresponding *activation* variables—$m_{\infty }^{{\text{Na}}_{\text{V}}1.2}\left(V\right)$ and $\tau_{m}^{{\text{Na}}_{\text{V}}1.2}\left(V\right)$—unchanged. The curve legended “$✚h_{\infty }^{{\text{Na}}_{\text{V}}1.2},\tau_{h}^{{\text{Na}}_{\text{V}}1.2}$” does just that, and likewise, in the “$▼m_{\infty }^{{\text{Na}}_{\text{V}}1.2},\tau_{m}^{{\text{Na}}_{\text{V}}1.2}$” curve, the Na_V_1.2 *activation* is shifted by Δ*V*_RS_ without affecting *availability*.

Further, we apply Δ*V*_RS_ to $\tau_{h}^{{\text{Na}}_{\text{V}}1.2}\left(V\right)$ without modifying any other gating properties—including $h_{\infty }^{{\text{Na}}_{\text{V}}1.2}\left(V\right)$—in the curve legended “$■\tau_{h}^{{\text{Na}}_{\text{V}}1.2}$”. We do the same for $h_{\infty }^{{\text{Na}}_{\text{V}}1.2}\left(V\right)$, $\tau_{m}^{{\text{Na}}_{\text{V}}1.2}\left(V\right)$ and $m_{\infty }^{{\text{Na}}_{\text{V}}1.2}\left(V\right)$, in the curves legended “$◆h_{\infty }^{{\text{Na}}_{\text{V}}1.2}$”, “$✖\tau_{m}^{{\text{Na}}_{\text{V}}1.2}$”, and “$▲m_{\infty }^{{\text{Na}}_{\text{V}}1.2}$” respectively. Fig 6 computes the new backpropagation threshold *I*_*BP*_(Δ*V*_RS_) under the aforementioned transformations. (For mathematical details, see Shift-Clamping and the Hodgkin-Huxley model in Materials and methods, and Section F.2 in S1 Text.)

An example transformation is given below by Eq 3, in which only $\tau_{h}^{{\text{Na}}_{\text{V}}1.2}\left(V\right)$ has its *right-shift* modified by Δ*V*_RS_ (see Fig Qii in S1 Text). The other three Na_V_1.2 variables have the nominal *right-shift* of 13mV. The *I*_*BP*_(Δ*V*_RS_) curves in Fig 6 that correspond to Eq 3 are legended $■\tau_{h}^{{\text{Na}}_{\text{V}}1.2}$:

$$■\tau_{h}^{{\text{Na}}_{\text{V}}1.2}\leftarrow \{\begin{array}{l} \tau_{h}^{{\text{Na}}_{\text{V}}1.2}=\tau_{h}^{{\text{Na}}_{\text{V}}1.2}\left(V-\Delta V_{\text{RS}}\right)\\ h_{\infty }^{{\text{Na}}_{\text{V}}1.2}=h_{\infty }^{{\text{Na}}_{\text{V}}1.2}\left(V\right)\\ \tau_{m}^{{\text{Na}}_{\text{V}}1.2}=\tau_{m}^{{\text{Na}}_{\text{V}}1.2}\left(V\right)\\ m_{\infty }^{{\text{Na}}_{\text{V}}1.2}=m_{\infty }^{{\text{Na}}_{\text{V}}1.2}\left(V\right)\text{.} \end{array}$$ (3)

(Fig Q in S1 Text visualizes the impact of Eq 3 on gating properties as a function of position at *V*_rest_.)

To unpack these additional curves, we begin at the coordinate we will call ‘the nominal point’ in each plot of Fig 6, which is the backpropagation threshold at Δ*V*_RS_ = 0, where all curves must intersect by definition. Starting at the nominal point, as one moves leftward along a given curve (Δ*V*_RS_ < 0), the gating properties indicated in the legend are *left-shifted* (e.g. Eq 3), and the other gating properties are left alone. Likewise, travelling away from the nominal point to the right (Δ*V*_RS_ > 0) will *right-shift* the indicated properties, relative to their nominal kinetics (Fig P in S1 Text).

Fig 6B reveals that, with axonal stimulation, the *right-shifted* *availability* ($h^{{\text{Na}}_{\text{V}}1.2}$) drives *I*_*BP*_. Specifically, the curves legended $◆h_{\infty }^{{\text{Na}}_{\text{V}}1.2}$, $■\tau_{h}^{{\text{Na}}_{\text{V}}1.2}$, and $✚h_{\infty }^{{\text{Na}}_{\text{V}}1.2},\tau_{h}^{{\text{Na}}_{\text{V}}1.2}$ show how $h_{\infty }^{{\text{Na}}_{\text{V}}1.2}$ (the *availability* at steady-state, as a function of membrane potential) and $\tau_{h}^{{\text{Na}}_{\text{V}}1.2}$ (the voltage-dependent time constant of *availability*) work together to promote backpropagation: They drive the reference curve (●) downward as Δ*V*_RS_ increases, in spite of the higher *activation* threshold. In other words: The threshold-lowering effects that result from *right-shifting* the *availability* overpower the opposing influence of *right-shifted* *activation*—on its own the latter would raise the threshold (see the curves: $▲m_{\infty }^{{\text{Na}}_{\text{V}}1.2}$ and $▼m_{\infty }^{{\text{Na}}_{\text{V}}1.2},\tau_{m}^{{\text{Na}}_{\text{V}}1.2}$ in Fig 6B).

Further, removing the *right-shift* from $\tau_{h}^{{\text{Na}}_{\text{V}}1.2}$ stops backpropagation: In the $■\tau_{h}^{{\text{Na}}_{\text{V}}1.2}$ curve of Fig 6B, all gating properties other than $\tau_{h}^{{\text{Na}}_{\text{V}}1.2}$, including $h_{\infty }^{{\text{Na}}_{\text{V}}1.2}$, retain their nominal *right-shift*, yet backpropagation ceases (according to our strict BAP criterion, see Section B in S1 Text) for axonal stimulation when $\Delta V_{\text{RS}}≲-2\text{mV}$.

For somatic stimulation, the *right-shifted activation* ($m^{{\text{Na}}_{\text{V}}1.2}$) drives *I*_*BP*_. Travelling from right to left in Fig 6A, the most significant decrease in threshold occurs in the curves legended $▲m_{\infty }^{{\text{Na}}_{\text{V}}1.2}$ and $▼m_{\infty }^{{\text{Na}}_{\text{V}}1.2},\tau_{m}^{{\text{Na}}_{\text{V}}1.2}$ as the nominal *right-shift* is removed ($\Delta V_{\text{RS}}\to -13.0\text{mV}\Rightarrow V_{\text{RS}}+\Delta V_{\text{RS}}\to 0$, see Fig P in S1 Text). The $▼m_{\infty }^{{\text{Na}}_{\text{V}}1.2},\tau_{m}^{{\text{Na}}_{\text{V}}1.2}$ curve differs negligibly from the $▲m_{\infty }^{{\text{Na}}_{\text{V}}1.2}$ curve, showing that $m_{\infty }^{{\text{Na}}_{\text{V}}1.2}$ *right-shift* dominates in raising the threshold near the nominal point, and the *right-shift* of $\tau_{m}^{{\text{Na}}_{\text{V}}1.2}$ matters little.

Our Δ*V*_RS_ results from Fig 6 are summarized in Table 1.

**Table 1:** Summary of sensitivity analysis: Impact of *right-shifted* Na_V_1.2 gating properties in the AIS on backpropagation threshold (see Fig 6).

Type of Stimulation	● reference curve	Increasing Δ*V*_RS_ of *availability*			Increasing Δ*V*_RS_ of *activation*		
		$■\tau_{h}^{{\text{Na}}_{\text{V}}1.2}$	$◆h_{\infty }^{{\text{Na}}_{\text{V}}1.2}$	$✚h_{\infty }^{{\text{Na}}_{\text{V}}1.2},\tau_{h}^{{\text{Na}}_{\text{V}}1.2}$	$✖\tau_{m}^{{\text{Na}}_{\text{V}}1.2}$	$▲m_{\infty }^{{\text{Na}}_{\text{V}}1.2}$	$▼m_{\infty }^{{\text{Na}}_{\text{V}}1.2},\tau_{m}^{{\text{Na}}_{\text{V}}1.2}$
Axonal	↓	↓	↓	↓	↑(slight)	↑	↑
Somatic	↑	×	↓(slight)	↓(slight)	×	↑	↑

Arrows (↓,↑) indicate the sign of the **slopes** of backpropagation threshold curves in Fig 6A (somatic stimulation) and Fig 6B (axonal stimulation). A downward arrow (↓) indicates that the backpropagation threshold *I*_*BP*_ decreases with increasing *right-shift*, applied to the gating properties specified above it. Likewise, an upward arrow (↑) indicates that the threshold increases when the specified combination of Na_V_1.2 variables is *right-shifted*. In cells marked “×” the effect of *right-shift* was negligible.

### Generalization to Hay-based model and modified Hu-based model

To demonstrate that our primary result—the separation of Na_V_1.2 and Na_V_1.6 into the proximal and distal AIS, respectively, promotes backpropagation with axonal stimulation but can *increase or decrease I*_*BP*_ with somatic stimulation—is not an artifact of our implementation of the model from Hu et al. (2009) [15] used thus far, we have inserted Na_V_ distributions analogous to Fig 1 into the model of Hay et al. (2011) [29] below. (Cell morphology in Fig B in S1 Text.)

(Also, in Section D in S1 Text, we include a modified version of the Hu-based model, with increased dendritic excitability and a less excitable soma. It has robust backpropagation in the entire dendritic tree without attenuation. Despite its 10× higher dendritic excitability, 3× lower somatic excitability, and qualitatively different backpropagation pattern, the results reported above are reproduced there as well.)

We replaced the single population of Na_V_s in the Hay model’s 60μm AIS with two Na_V_ subtypes, based on their original Na_V_ kinetics: One population *left-shifted* by 6mV and the other *right-shifted* by 6mV, relative to the original *V*_1/2_, to represent Na_V_1.6 and Na_V_1.2 respectively. Our manipulations of the Na_V_ channels’ distribution (varying *κ* and *x*) did not change the total Na_V_ density in the AIS, which was kept identical to their model (https://modeldb.science/139653). Further, we attached an additional 400μm-long section of passive cable to the end of the AIS, where their axon originally stopped, to allow the AP to exit the AIS orthodromically as well as antidromically, as is the case in real neurons, in order to make AP generation in the Hay model more realistic. This was necessary to recover our qualitative results.

Our intention was to modify the Hay model as little as was necessary, since its parameters are tailored to a specific neuron and morphology—they will not necessarily transfer well even between specimens of the same cell type (see Hay et al. (2011) [29]). Presumably, the tuning may also be sensitive to the excitability of newly attached compartments.

We note that Hay et al. optimized their models to fit experimentally observed somatic and dendritic spiking patterns, including BAC firing, but their focus was not on action potential initiation. The models that best fit their data had AP initiation in the soma rather than the AIS, but they provide an additional model where APs were constrained to initiate in a 60μm section named “axon”, which had been set aside due to excessive BAP attenuation (see [29]). Since we required a parameter tuning with AP initiation in the AIS, the latter model was the necessary choice, despite its unrealistically strong attenuation of the backpropagated AP.

**Fig 7:**
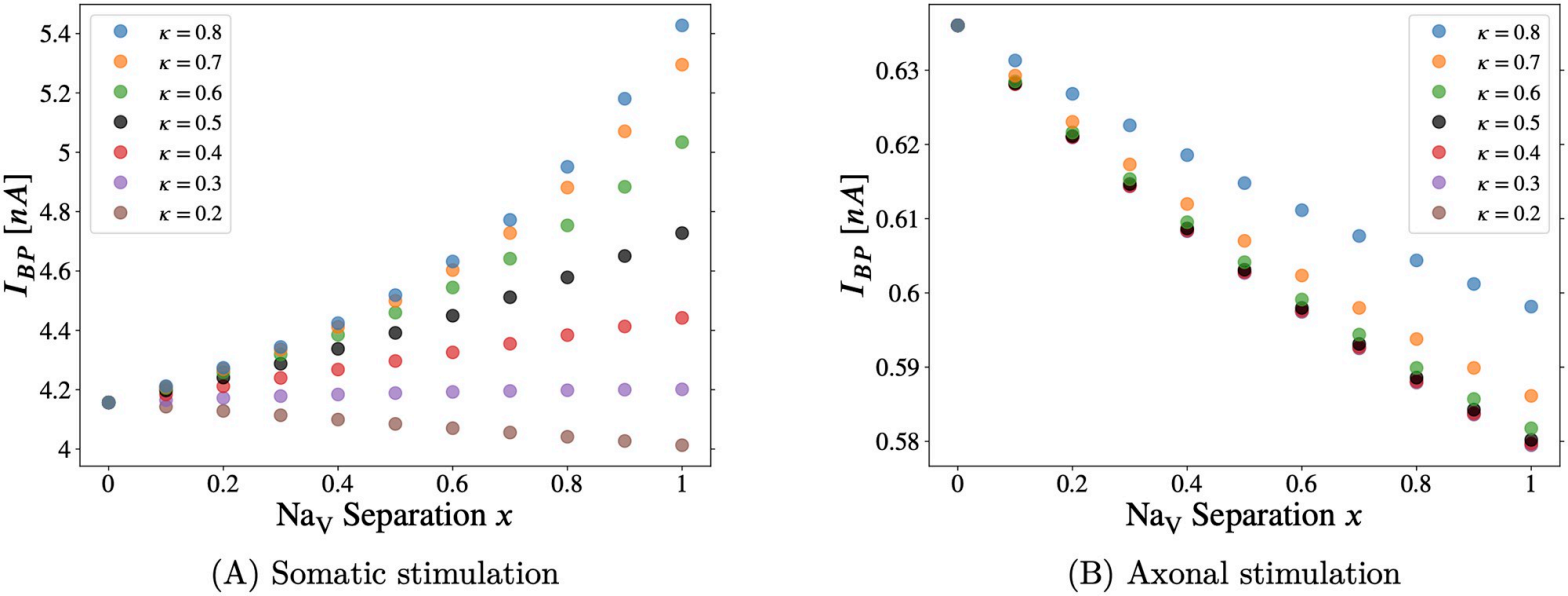
Backpropagation threshold in the Hay model: Combined effect on the backpropagation threshold (*I*_*BP*_, defined below) of varying crossover location (*κ*) and Na_V_ separation (*x*) in the axon initial segment. Varying the separation parameter “*x*” from *x* = 0 to *x* = 1, the distribution of Na_V_ channels goes from flat (homogeneous) to separated, the latter approximating the distribution observed in developing pyramidal neurons (see Fig 1A, [20]). Note that curves for all values of *κ* converge to a single point at *x* = 0, since *κ* can have no effect when the two Na_V_ subtypes are uniformly distributed along the AIS. Apical dendrite backpropagation criterion =-70.0mV—see caption of Fig E in S1 Text. In the Hay model, the forward propagation threshold *I*_*FP*_ is not defined: There is no nowhere for saltatory conduction to occur, as there is no excitable axon beyond the AIS [29].

In Fig 7 we register backpropagation in the Hay model [29] as a depolarization of several mV in the apical dendrites, following current injection. Identical results using a somatic backpropagation measurement criterion are included as Fig F in S1 Text. The threshold was set at -70mV when measuring the depolarization near the bifurcation of the main apical dendrite, where $V_{\text{rest}}≅-74.1\text{mV}$. See Fig E in S1 Text.

Note the qualitative agreement between the Hay-based model implemented and the Hu-based model above (and the modified Hu-based model in the Supporting Information (Section D in S1 Text)). In Fig 7A, we simulate backpropagation following somatic stimulation. As above in Fig 2 (and Fig H in S1 Text), concentrating Na_V_1.2 in the proximal AIS tends to raise the backpropagation threshold, and increasing the proportion of total sodium conductance in the AIS allocated to Na_V_1.6 lowers *I*_*BP*_.

In Fig 7B, we simulate backpropagation following axonal stimulation. As above in Figs 4 and 5 (and Fig J in S1 Text), the separated Na_V_ distribution (*x* → 1) lowers the threshold in the Hay model. Quantitatively, Fig 7B is closer to Fig 5, suggesting that the concentration of low-threshold Na_V_1.6 at the distal AIS, rather than the concentration of Na_V_1.2 at the proximal AIS, promotes backpropagation. What is important to keep in mind is that, in both models, concentrating Na_V_1.2 in the proximal AIS only lowered *I*_*BP*_ in the case of depolarizing axonal current injection.

### Rescaling the Na_V_1.2 density profile by a uniform factor in the AIS

**Fig 8:**
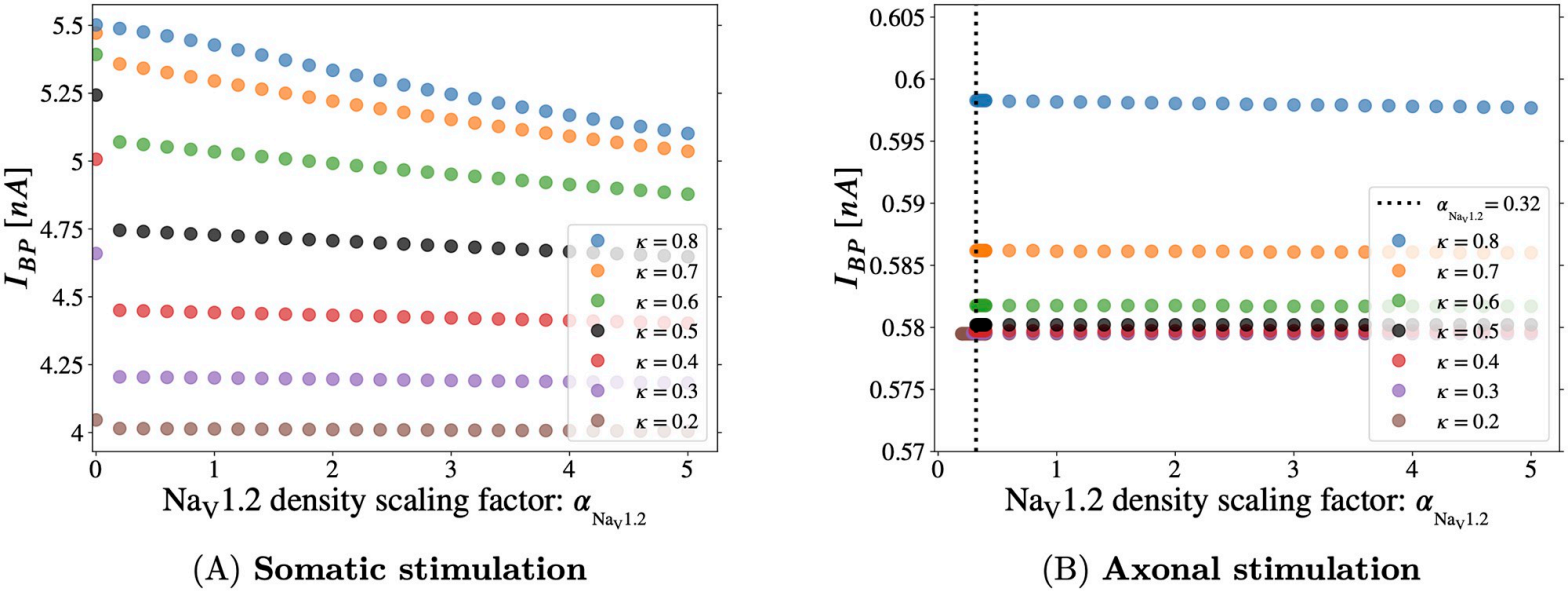
Scaling the Na_V_1.2 density profile in the AIS of the Hay-based model [29], with somatic vs. axonal stimulation. The backpropagation threshold is computed while the local Na_V_1.2 density is rescaled by $\alpha_{{\text{Na}}_{\text{V}}1.2}$ at every AIS segment. Here we have set *x* = 1 so that the Na_V_1.2 and Na_V_1.6 density profiles are separated, guaranteeing that the proximal AIS is exclusively populated with Na_V_1.2—see Fig 1. With somatic stimulation **(A)**, backpropagation persists even when Na_V_1.2 is completely removed from the AIS ($\alpha_{{\text{Na}}_{\text{V}}1.2}=0$). However, with axonal stimulation **(B)**, backpropagation ends abruptly near $\alpha_{{\text{Na}}_{\text{V}}1.2}≲0.32$. Yet again, the importance of the proximal Na_V_1.2 subtype and its qualitative effects on excitability depend heavily on the mode of stimulation. Density of data points is increased near the vertical dashed line to detect backpropagation cutoff.

In this section, we rescale the Na_V_1.2 density profile in the Hay-based model via the maximal conductance ${\bar{g}}_{{\text{Na}}_{\text{V}}1.2}$. At each segment of the AIS, ${\bar{g}}_{{\text{Na}}_{\text{V}}1.2}$ is multiplied by a positive number which we call the Na_V_1.2 scaling factor, denoted $\alpha_{{\text{Na}}_{\text{V}}1.2}$. That is, at each point *s* in the AIS,

$${\bar{g}}_{{\text{Na}}_{\text{V}}1.2}\left(s\right)\to \alpha_{{\text{Na}}_{\text{V}}1.2}{\bar{g}}_{{\text{Na}}_{\text{V}}1.2}\left(s\right)\text{,}$$ (4)

with $\alpha_{{\text{Na}}_{\text{V}}1.2}⩾0$. In Fig 8A the backpropagation threshold is computed with somatic current injection (see Fig E in S1 Text). We observe that reducing the density of Na_V_1.2, without adding any compensatory Na_V_1.6 density, increases the threshold as expected—the slope is most visible on the *κ* = 0.8 line of Fig 8A, wherein the majority of the AIS (except the most distal region, see Fig 1B) contains Na_V_1.2 and is therefore affected by $\alpha_{{\text{Na}}_{\text{V}}1.2}$. Even when Na_V_1.2 is completely removed from the AIS, and consequently the proximal AIS contains no Na_V_ channels, backpropagation is possible with somatic stimulation.

In Fig 8B it is interesting to see, yet again, the sharp qualitative difference in the role of the Na_V_1.2 subtype with axonal versus somatic stimulation. As noted above, backpropagation is present in Fig 8A when the AIS Na_V_1.2 channels are disabled entirely ($\alpha_{{\text{Na}}_{\text{V}}1.2}=0$). With axonal stimulation however (Fig 8B), the effect of $\alpha_{{\text{Na}}_{\text{V}}1.2}$ was abrupt and binary, akin to a Heaviside function. *I*_*BP*_ was nearly flat, except the Hay neuron did not produce a BAP when $\alpha_{{\text{Na}}_{\text{V}}1.2}≲0.32$—some nonzero Na_V_ density was required in the proximal AIS for backpropagation.

With somatic stimulation, the neuron is primed for backpropagation by the electrode current, which depolarizes the soma before the AP arrives from the AIS. As $\alpha_{{\text{Na}}_{\text{V}}1.2}\to 0$, increased somatic stimulation can compensate for the resulting loss of depolarizing Na_V_ current from the proximal AIS.

With axonal stimulation, there is no direct pre-charging of the soma by the injected current pulse. When $\alpha_{{\text{Na}}_{\text{V}}1.2}$ decreases the density of channels at the proximal AIS, the electrode cannot compensate for the lost Na_V_1.2 current, due to its electrical isolation from the soma. The abrupt BAP cutoff reflects the all-or-none nature of action potentials. The lack of slope in *I*_*BP*_ owes to the fact that the axonal current pulse depolarizes the most distal end of the AIS, which sets the threshold in this case [15] (provided the proximal Na_V_ density is sufficient, see above). The distal AIS is populated exclusively by Na_V_1.6 channels, and hence it is unaffected by $\alpha_{{\text{Na}}_{\text{V}}1.2}$.

## Discussion

In early development, pyramidal neurons concentrate Na_V_1.2 in the proximal AIS, and Na_V_1.6 in the distal AIS. As these cells mature, Na_V_1.6 invades the proximal AIS, and the two Na_V_ subtypes lose their separated distribution [20]. We have investigated the effects of Na_V_ separation in the axon initial segment on the initiation and backpropagation of action potentials in three different pyramidal neuron models. In spite of their different parameters, axonal and dendritic morphology, and biophysics, all three models (see: Somatic stimulation, Axonal stimulation, Modifying the *right-shift* of Na_V_1.2 gating properties in the AIS, Generalization to Hay-based model and modified Hu-based model, and Section D in S1 Text) indicated that the effects of the separated Na_V_ distribution depend on whether stimulation is orthodromic (e.g. somatodendritic input) or antidromic (e.g. axonal stimulation).

With somatic stimulation, the greater the proportion of Na_V_1.2 in the AIS, relative to Na_V_1.6, the less excitable the cell becomes (increased *I*_*BP*_). Our somatic current injection results are contrary to past modelling which used axonal stimulation [15], although they are consistent with more recent experimental results that used somatic stimulation [18]. The threshold-raising effect of proximal Na_V_1.2 is confirmed by repeating the simulations with a model cell in which the AIS has been flipped longitudinally (Fig 3), placing Na_V_1.6 proximally and Na_V_1.2 distally in the AIS.

Our results using axonal stimulation agree qualitatively with and expand upon past modelling efforts [15]: In all three models, with axonal current injection, excitability is greatest (lowest *I*_*BP*_) when Na_V_ subtypes are separated in the AIS (‘*x*-shaped distribution’). Further, in the Hu-based models (Fig 4, Fig J in S1 Text), increasing the total proportion of Na_V_1.2 in the AIS—by moving the Na_V_ crossover *κ* distally—promotes backpropagation as well. In the Hay-based model, removing Na_V_s from the proximal AIS halted backpropagation. We also find that increased distal Na_V_1.6 concentration (which results from the separated distribution) lowers the AP threshold (Fig 5).

Testing both modes of stimulation can contribute to resolving inconsistencies between experiments such as [18] and [15], where stimulation was orthodromic in the former and antidromic in the latter. In [18], AP initiation was observed in pyramidal neurons which were engineered to be Na_V_1.6-deficient. In those neurons, the AIS was populated entirely with Na_V_1.2, however they still found that the AIS Na_V_ current was *left-shifted* relative to the somatic current. From this and other observations, the authors in [18] suggest that the distribution of Na_V_ subtypes is not so important in shifting the local voltage-gated Na^+^ current.

We note that, compared to control neurons, the Na_V_1.6-deficient neurons’ AIS Na_V_ current was *right-shifted*, and the orthodromic AP threshold (amplitude of a 2ms current pulse [18]) was nearly doubled. This is consistent with our results and the modelling assumption that *right-shift* is associated with Na_V_1.2 in the AIS—the model is agnostic about the molecular details. The decrease in excitability reported in [18] may have been even larger had they used more mature neurons. Their neurons were obtained from 4–5 week old mice, at which point the AIS will still be largely populated with Na_V_1.2, whereas in wild type mice Na_V_1.6 replaces much of the Na_V_1.2 by 90 days [20, 30]. Our results indicate that with axonal stimulation, Na_V_1.6-deficient cells may have a lower backpropagation threshold than the wild type.

The loss of the separated Na_V_ distribution in the AIS at later developmental stages, accompanied by the proximal localization of Na_V_1.6, may enhance excitability to healthy orthodromic stimulation while protecting against the backpropagation of ectopic activity from damaged axons into the soma and dendrites. Further, research into the genetic causes of autism spectrum disorder has revealed that Na_V_1.2 knockout can enhance pyramidal cells’ tendency to send action potentials and simultaneously reduce backpropagation (somatodendritic hypoexcitability) [19]. Whereas [19] reported an interplay between Na_V_1.2 and K_V_, in contrast, our results are explained by the spatial distribution of Na_V_ *right-shift* within the AIS (Table 1, Fig 6A). Indeed, the reduced excitability resulting from AIS Na_V_1.2 owes to the asymmetric impact of *availability* on backpropagation in axonal versus somatic stimulation (Fig 6).

Although *right-shifting* Na_V_1.2 steady-state *availability*, $h_{\infty }^{{\text{Na}}_{\text{V}}1.2}\left(V\right)$, in the AIS is necessary to promote backpropagation (i.e. decrease *I*_*BP*_) when stimulation is axonal, it is not sufficient on its own. Our modelling shows that the voltage-sensitive time constant of *availability*, $\tau_{h}^{{\text{Na}}_{\text{V}}1.2}\left(V\right)$, must be *right-shifted* as well (Fig 6B, $■\tau_{h}^{{\text{Na}}_{\text{V}}1.2}$ curve).

It is straightforward to explain why increasing Δ*V*_RS_ lowers *I*_*BP*_ in the $◆h_{\infty }^{{\text{Na}}_{\text{V}}1.2}$ curves of Fig 6A and 6B: *Right-shifting* steady-state Na_V_1.2 *availability* increases Na^+^ conductance at all voltages because $h_{\infty }^{{\text{Na}}_{\text{V}}1.2}\left(V\right)$ is monotonically decreasing (Fig Pi in S1 Text). However, without sensitivity analysis, it was *not* obvious that removing the nominal *right-shift* from the voltage-sensitive time constant $\tau_{h}^{{\text{Na}}_{\text{V}}1.2}$—without modifying steady-state *activation* ($m_{\infty }^{{\text{Na}}_{\text{V}}1.2}$) or *availability* ($h_{\infty }^{{\text{Na}}_{\text{V}}1.2}$)—would on its own be sufficient to eliminate the *I*_*BP*_-lowering effects of Na_V_1.2 for axonal stimulation. This effect is demonstrated in the curve legended $■\tau_{h}^{{\text{Na}}_{\text{V}}1.2}$ of Fig 6B. It follows that the *x*-distribution’s tendency to promote backpropagation is not merely a result of increased steady-state *availability* of proximal AIS Na_V_s, but is a dynamic effect—dependent on the *right-shift* of $\tau_{h}^{{\text{Na}}_{\text{V}}1.2}\left(V\right)$ as well.

From Fig Pii in S1 Text, we can see that *right-shifting* Na_V_1.2 slows down the *inactivation* process via its time constant—which is the voltage-sensitive time constant of *availability*. The membrane potential traverses the $\tau_{h}^{{\text{Na}}_{\text{V}}1.2}\left(V\right)$ curve during an AP. *Right-shifting* $\tau_{h}^{{\text{Na}}_{\text{V}}1.2}\left(V\right)$ moves the maximum value of the time constant to depolarized voltages, and slows down *inactivation* so that more channels are *available* to assist with backpropagation.

There is an interplay between cable properties and the distribution of Na_V_s in determining the site of AP initiation [44]. Electrical isolation of the initiation site may amplify the effect of concentrating Na_V_1.6 in the distal AIS. Via fluorescence imaging of intracellular Na^+^ concentration following single action potentials, [35] located the greatest Na^+^ influx at the middle of the AIS, whereas the distal AIS (initiation site) had only 1/4 of this maximum. They inferred that the density of Na_V_ channels decreases toward the initiation site, and thus Na_V_ density does not determine the precise location where APs begin.

Although [35] did not require Na_V_1.6 accumulation at the distal AIS to explain the distal location of the initiation site, the authors suggest that local Na_V_ density can have a large effect on neuronal excitability. Temperature may also play a role in local AIS Na^+^ influx measurements due to the spatial separation of Na_V_ subtypes. The pyramidal neurons in [35] and [18] were cooled to ≅ 21°, and Na_V_1.2 and Na_V_1.6 differ in their responses to temperature changes [17]. Thus, a deeper exploration of the effects of temperature on AP initiation is warranted.

The temporal resolution of Na^+^ influx measurements continues to improve: [45] achieved a resolution of 0.1ms imaging pyramidal cells in mouse brain slices. Another order of magnitude improvement may be sufficient to discern the local contributions of Na_V_ subtypes to AP initiation. The qualitative dependence of the backpropagation threshold on the somatic-versus-axonal mode of stimulation is compatible with distal AP generation as found in [15, 35] and in our work, but does not seem to rely crucially on the precise determinants of AP onset position; it relies rather on the *activation* and *availability* properties, and the kinetics of the latter.

The effect of realistic synaptic input is a broad interesting question that is beyond the scope of this study. Furthermore, including it at this point would defeat the purpose of comparing axonal versus somatic stimulation, since antidromic axonal input will be in the form of a brief pulse (no excitatory synaptic input typically occurs onto axons of pyramidal neurons, and when it does it generates an AP). Because our simulations compare orthodromic stimulation to antidromic stimulation, the shape of the injected current must be kept identical in each mode to isolate the effect of the Na_V_ density profiles on *I*_*BP*_.

We would expect that fast glutamatergic input near the soma, or propagating to the soma from sufficiently synchronized dendritic synaptic inputs, would yield qualitatively similar results as reported here. There may be qualitative changes if the synaptic input has a slower rise time, e.g. for synapses with a large NMDA component.

Since the Na_V_ distribution changes throughout development, a further investigation—beyond the scope of this paper, as we will explain—would be to understand how accompanying developmental changes in morphological complexity and voltage-gated channel density elsewhere in the neuron [46] interact with developmental plasticity in the AIS. This would require new parameter sets at each iteration of the morphological complexity. Since Hay et al. [29] had to fit each morphology’s parameter set to match firing patterns observed in real neurons, that procedure would need to be repeated. If sufficient experimental data are not available to perform the fitting at each iteration, new electrophysiological experiments would be necessary at the corresponding developmental stages. That endeavour is beyond the scope of the present study. Also, there is experimental evidence that AIS plasticity is not limited to development [6]. Our strategy was to restrict our investigation to the effects of varying the heterogeneous distribution of Na_V_ subtypes in the AIS on backpropagation threshold, with different modes of stimulation. Note, that the changing Na_V_ distributions we simulate are not strictly intended to replicate observed plasticity. Even if the Na_V_ distribution in the AIS of real neurons were static, modelling the hypothetical distributions would nonetheless assist in understanding its function via the resulting changes to cellular excitability.

Our model neurons were kept identical in all results presented above; only the AIS was altered. Our results therefore can only be explained by the distribution of Na_V_ subtypes (or, the distribution of *right-shifted* Na_V_ gating properties) within the AIS. Given the *τ*_*h*_-dependence of the antidromic backpropagation threshold in Modifying the *right-shift* of Na_V_1.2 gating properties in the AIS, and the differential temperature sensitivity of Na_V_1.2 versus Na_V_1.6 [17], there is good reason to expect that the effects of Na_V_ separation predicted here will be temperature-dependent.

In summary, we have simulated a range of hypothetical Na_V_ distributions in the axon initial segment of three 3D-reconstructed biophysical pyramidal cell models, including two distinct morphologies and three different parameter tunings. Our modelling shows that the spatial profile of Na_V_1.2 and Na_V_1.6 in the AIS and the kinetics of their *availability* and *activation* are important determinants of excitability and the backpropagation threshold. We predict that the separation of Na_V_ subtypes observed in early development has an asymmetrical effect on excitability which depends on whether the neuron is stimulated orthodromically or antidromically. With orthodromic stimulation, Na_V_ separation impedes backpropagation and reduces excitability unless the crossover is brought close to the soma. Backpropagation and excitability are both enhanced by Na_V_ separation when stimulation is antidromic. Maintaining a static Na_V_ distribution, we altered the *right-shift* of selected Na_V_1.2 gating properties. This revealed that steady-state *activation right-shift* controls the orthodromic backpropagation threshold, and dynamic *availability right-shift* is necessary to explain the antidromic threshold. Furthermore, given that learning is linked to backpropagation, the evolving separation of the Na_V_ subtypes may impact synaptic weight modification across developmental stages.

## Materials and methods

The pyramidal cell models (Figs A and B in S1 Text) were implemented in NEURON 8.0 [47] via Python. For cell geometry, local membrane properties, additional simulations, and a variety of calculations, clarifications, and definitions, see S1 Text, which has its own table of contents.

Our Hay-based model is biophysically and morphologically identical to the original [29], aside from the modified *right-shift* in axonal Na_V_ channels that we introduced to create Na_V_1.2 and Na_V_1.6 variants in the AIS, and an additional passive section attached to the end of the axon. Our implementation of the Hay model (https://modeldb.science/139653) is detailed in Generalization to Hay-based model and modified Hu-based model.

Our Hu-based model [15] uses the same reconstructed morphology as the original model (https://modeldb.science/123897), which is a Layer 5 pyramidal neuron from cat visual cortex, modified from [26] (see SI, Section A in S1 Text). We added explicit intracellular and extracellular concentrations of sodium, potassium, and chloride ions. Because of this change, the Nernst potentials $E_{{\text{Na}}^{+}}$, $E_{{\text{K}}^{+}}$, $E_{{\text{Cl}}^{-}}$ are calculated locally from each compartment’s specific ionic concentrations, which respond to transmembrane currents. The Na_V_1.2, Na_V_1.6, and K_V_ kinetics from [15] are included as well.

We also included active transport via a Na^+^/K^+^-pump current, to maintain the transmembrane concentration gradients of Na^+^ and K^+^. In our Hu-based model, all ions are subject to longitudinal diffusion, both intra- and extracellular, implemented using NEURON’s RxD facility [48, 49]. The cell maintains a resting potential $V_{\text{rest}}≅-70\text{mV}$ at steady-state, and restores this state following stimulation. The biophysics that governs local ion concentrations (and Nernst potentials) in the Hu-based model is summarized in Biophysics, Hu-based model.

### AIS—Na_V_ density profiles

In all of the models presented in this study, the density profiles of Na_V_1.2 and Na_V_1.6 are left- and right-handed sigmoidal functions (respectively) of normalized length *s* along the AIS. The proximal end of the AIS is located at *s* = 0, and the distal end is located at *s* = 1. The channel densities are expressed as maximal conductances ${\bar{g}}_{{\text{Na}}_{\text{V}}1.6}$ (s) and ${\bar{g}}_{{\text{Na}}_{\text{V}}1.2}$ (s), where the total maximal Na_V_ conductance ${\bar{g}}_{{\text{Na}}_{\text{V}}}$ is constant along the AIS:

$${\bar{g}}_{{\text{Na}}_{\text{V}}}={\bar{g}}_{{\text{Na}}_{\text{V}}1.2}(s)+{\bar{g}}_{{\text{Na}}_{\text{V}}1.6}\left(s\right)=\text{const}.$$ (5)

The density profiles are given by

$$\{\begin{array}{ll} {\bar{g}}_{{\text{Na}}_{\text{V}}1.2}(s) & =\frac{{\bar{g}}_{{\text{Na}}_{\text{V}}}}{2}(1-x\cdot \text{tanh}(\sigma (s-\kappa ))),\\ {\bar{g}}_{{\text{Na}}_{\text{V}}1.6}\left(s\right) & ={\bar{g}}_{{\text{Na}}_{\text{V}}}-{\bar{g}}_{{\text{Na}}_{\text{V}}1.2}(s)\\ & =\frac{{\bar{g}}_{{\text{Na}}_{\text{V}}}}{2}(1+x\cdot \text{tanh}(\sigma (s-\kappa ))). \end{array}$$ (6)

We chose the hyperbolic tangent function tanh(*s*), but other sigmoidal functions would do just as well. The parameter *x* controls the separation of the Na_V_ distribution, that is, how separated the two Na_V_ subtypes are. When *x* = 0, the distribution becomes flat—Na_V_1.2 and Na_V_1.6 are mixed uniformly along the AIS. When *x* = 1, the proximal end of the AIS contains only Na_V_1.2, and the distal end of the AIS contains only Na_V_1.6. The parameter *σ* is the reciprocal of the ‘transition width’ of the AIS Na_V_ distributions normalized by the AIS length. In all simulations shown here, *σ* = 10.0. Additional details are provided in Section E in S1 Text.

### Shift-Clamping and the Hodgkin-Huxley model

Here we provide additional details of the sensitivity analysis performed in Fig 6. In the Hodgkin-Huxley model [40] a gating variable *u* evolves according to its voltage-dependent forward and backward transition rates *α*_*u*_(*V*) and *β*_*u*_(*V*) as

$$\begin{array}{c} \frac{du}{dt}=\alpha_{u}\left(V\right)[1-u]-\beta_{u}\left(V\right)u\text{,} \end{array}$$ (7)

where *u* could be Na_V_ *activation m* or *availability h*, or K_V_ *activation n*, etc. This can be rewritten using the steady-state *u*_∞_(*V*) and voltage-dependent time constant *τ*_*u*_(*V*) of the gating variable

$$\begin{array}{c} \frac{du}{dt}=\frac{u_{\infty }\left(V\right)-u}{\tau_{u}\left(V\right)}, \end{array}$$ (8)

where *u*_∞_ and *τ*_*u*_ are computed from *α*_*u*_ and *β*_*u*_ via

$$\begin{array}{c} u_{\infty }\left(V\right)=\frac{\alpha_{u}\left(V\right)}{\alpha_{u}\left(V\right)+\beta_{u}\left(V\right)}\;\text{and}\;\tau_{u}\left(V\right)=\frac{1}{\alpha_{u}\left(V\right)+\beta_{u}\left(V\right)}\;\;\;\text{.} \end{array}$$ (9)

When shifting the voltage-dependence of *u*_∞_ by Δ*V*_RS_ (see Modifying the *right-shift* of Na_V_1.2 gating properties in the AIS), it is natural to assume that one should apply the same shift to *τ*_*u*_ given Eq 9, since *u*_∞_ and *τ*_*u*_ are both functions of *α*_*u*_(*V*) and *β*_*u*_(*V*) in such models. However, our simulations can shift *u*_∞_(*V*) or *τ*_*u*_(*V*) independently of one another: e.g. $\tau_{u}^{\prime }\left(V\right)=\tau_{u}\left(V-\Delta V_{\text{RS}}\right)$, $u_{\infty }^{\prime }\left(V\right)=u_{\infty }\left(V\right)$. The forward and backward rates become

$$\begin{array}{c} \alpha_{u}^{\prime }=\frac{u_{\infty }^{\prime }\left(V\right)}{\tau_{u}^{\prime }\left(V\right)}\;\text{and}\;\beta_{u}^{\prime }=\frac{1-u_{\infty }^{\prime }\left(V\right)}{\tau_{u}^{\prime }\left(V\right)}\text{.} \end{array}$$ (10)

Putting this to use, one can modify the *right-shift* of combinations of

$$\begin{array}{c} \{\tau_{h}^{{\text{Na}}_{\text{V}}1.2}\;\;\left(V\right),\;h_{\infty }^{{\text{Na}}_{\text{V}}1.2}\;\;\left(V\right),\;\tau_{m}^{{\text{Na}}_{\text{V}}1.2}\;\;\left(V\right),\;m_{\infty }^{{\text{Na}}_{\text{V}}1.2}\;\;\left(V\right)\}\text{,} \end{array}$$ (11)

by adding “−Δ*V*_RS_” to the argument of the selected variables’ *u*_∞_(*V*)s or *τ*_*u*_(*V*)s.

### Biophysics, Hu-based model

Action potentials propagate via the cable equation

$$\begin{array}{c} C\frac{\partial V}{\partial t}=\frac{d}{4R_{a}}\frac{\partial^{2}V}{\partial s^{2}}-I_{\text{membrane}}\text{,} \end{array}$$ (12)

where *V* is the membrane potential, *C* is the specific membrane capacitance, *d* is the neurite diameter, *R*_*a*_ is the axial resistance, *s* is the position along the axis of the cable, and *I*_membrane_ is the total transmembrane current density of all ion species in the model.

Here we describe the currents in our Hu-based model. (The changes we made to the Hay model are described in Generalization to Hay-based model and modified Hu-based model.) In the Hu-based model, we added explicit intracellular and extracellular concentrations of sodium, potassium, and chloride ions at each compartment. We denote the intracellular/extracellular concentration of a given ionic species “Z” as [Z]_in_, [Z]_out_ respectively. These concentrations depend on the spatial coordinate—i.e. [Z]_in_ = [Z]_in_(*s*)—but that is not written explicitly, to simplify the notation. The Nernst potentials (reversal potentials) $E_{{\text{Na}}^{+}}$, $E_{{\text{K}}^{+}}$, $E_{{\text{Cl}}^{-}}$ of Na^+^, K^+^, and Cl^-^ are not fixed parameters but are instead determined by the intracellular and extracellular concentrations of those ions:

$$\begin{array}{c} E_{\text{Z}}\left(s\right)=-\frac{kT}{q_{\text{z}}}\text{ln}\left(\frac{{\left[\text{Z}\right]}_{\text{in}}}{{\left[\text{Z}\right]}_{\text{out}}}\right)\text{.} \end{array}$$ (13)

Transmembrane concentration gradients of Na^+^ and K^+^ are governed by active transport (Na^+^/K^+^-pump) and longitudinal diffusion. At each time step, ionic concentrations all over the cell are updated using transmembrane currents (Eq 16) and Fick’s law. At the *j*^th^ compartment this gives:

$$\begin{array}{c} \left\{ \begin{array}{c} \frac{\partial }{\partial t}{\left[\text{Z}\right]}_{\text{in}}^{j}=-(\frac{A^{j}}{FVol_{\text{in}}^{j}})I_{\text{Z}}^{j}+D_{\text{Z}}\nabla^{2}{\left[\text{Z}\right]}_{\text{in}}^{j}\\ \frac{\partial }{\partial t}{\left[\text{Z}\right]}_{\text{out}}^{j}=(\frac{A^{j}}{FVol_{\text{out}}^{j}})I_{\text{Z}}^{j}+D_{\text{Z}}\nabla^{2}{\left[\text{Z}\right]}_{\text{out}}^{j}\text{,} \end{array}\right. \end{array}$$ (14)

where $I_{\text{Z}}^{j}$ is the transmembrane current density of ion species Z at compartment *j*, with Z = Cl^-^, K^+^, Na^+^. *D*_Z_ denotes the diffusion coefficient of ion Z. *A*^*j*^ and $Vol_{\text{in}(\text{out})}^{j}$ are (respectively) the membrane area and intracellular/extracellular volume at the *j*^th^ compartment. *F* is the Faraday constant. The total transmembrane current density at the *j*^th^ compartment is

$$I_{\text{membrane}}^{j}=I_{{\text{Cl}}^{-}}^{j}+I_{{\text{K}}^{+}}^{j}+I_{{\text{Na}}^{+}}^{j}.$$ (15)

Omitting the compartment index *j*, the specific transmembrane currents are

$$I_{\text{membrane}}\leftarrow \{\begin{array}{l} I_{{\text{Cl}}^{-}}=g_{{\text{Cl}}^{-}}(V-E_{{\text{Cl}}^{-}})\\ I_{{\text{K}}^{+}}=(g_{{\text{K}}_{\text{V}}}+g_{\text{K},\text{leak}})(V-E_{{\text{K}}^{+}})-2I_{pump}\\ I_{{\text{Na}}^{+}}=(g_{{\text{Na}}_{\text{V}}}+g_{\text{Na},\text{leak}})(V-E_{{\text{Na}}^{+}})+3I_{pump}. \end{array}$$ (16)

$g_{{\text{Cl}}^{-}},g_{\text{K},\text{leak}}$, and $g_{\text{Na},\text{leak}}$ are passive leak conductances whereas $g_{{\text{K}}_{\text{V}}}$ and $g_{{\text{Na}}_{\text{V}}}$ have voltage-gated Hodgkin-Huxley (HH)-style kinetics (Eq S6 in S1 Text). Since channels are nonuniformly distributed along the cell membrane, conductances vary with location. *I*_*pump*_ is the net current produced by the Na^+^/K^+^-pump as a function of [K^+^]_out_ and [Na^+^]_in_,

$$I_{pump}=I_{maxpump}(1+\frac{K_{M_{{\text{K}}^{+}}}}{{\left[{\text{K}}^{+}\right]}_{\text{out}}}{)}^{-2}(1+\frac{K_{M_{{\text{Na}}^{+}}}}{{\left[{\text{Na}}^{+}\right]}_{\text{in}}}{)}^{-3},$$ (17)

where *I*_*maxpump*_ controls the maximal pump current, $K_{M_{{\text{K}}^{+}}}$ and $K_{M_{{\text{Na}}^{+}}}$ are Michaelis-Menten kinetic constants, and the Na^+^ and K^+^ currents flowing through the pump are $I_{{\text{Na}}^{+},pump}=3I_{pump}$ and $I_{{\text{K}}^{+},pump}=-2I_{pump}$. (Calcium dynamics are omitted in this section since Hu et al. [15] did not include the dendritic calcium spike initiation zone—see [39]. In Generalization to Hay-based model and modified Hu-based model, we include the Hay model, which features state-of-the-art calcium dynamics.).

## Supporting information

S1 Text For cell geometry, local membrane properties, additional simulations, and a variety of calculations, clarifications, and definitions, see this file. (PDF)
