# Supplementary material for: Impact on backpropagation of the spatial heterogeneity of sodium channel kinetics in the axon initial segment
Source: PLoS Comput Biol. 2024 Mar 15;20(3):e1011846. doi: 10.1371/journal.pcbi.1011846 (PMC10942053; doi:10.1371/journal.pcbi.1011846)
Supplement: S1 Text — (PDF) [file pcbi.1011846.s001.pdf]

S1 Text: Supporting Information

Contents

A Model: pyramidal cell geometry . . . 2

B Spikes and backpropagation in the Hu-based model . . . 4

C Spikes and backpropagation in the Hay-based model . . . 6

C.1 Identical results with somatic criterion . . . 7

D Modified version of Hu-based model . . . 8

D.1 Additional simulations . . . 14

E AIS - technical details . . . 18

F Voltage-gated channels . . . 19

F.1 Defining V_{RS}: the *right-shift* of $\mathrm{Na_V}1.2$ . . . 21

F.2 Notation: V_{RS}, ΔV_{RS} . . . 22

F.3 Space plots of $\mathrm{Na_V}$ kinetics along the AIS — steady-state . . . 23

G Transformed backpropagation threshold data . . . 25

H Diffusion coefficients . . . 26

I Tables of parameters . . . 27

A Model: pyramidal cell geometry

Fig A displays the first of the two neurons used in this paper. The dendritic morphology is a digital reconstruction of a Layer 5 pyramidal neuron from cat visual cortex, modified from [26]. These neurons have dendrites roughly as tall as the thickness of the cortex (several mm) and axon initial segment length similar to the width of a human hair (tens of μm [9]).

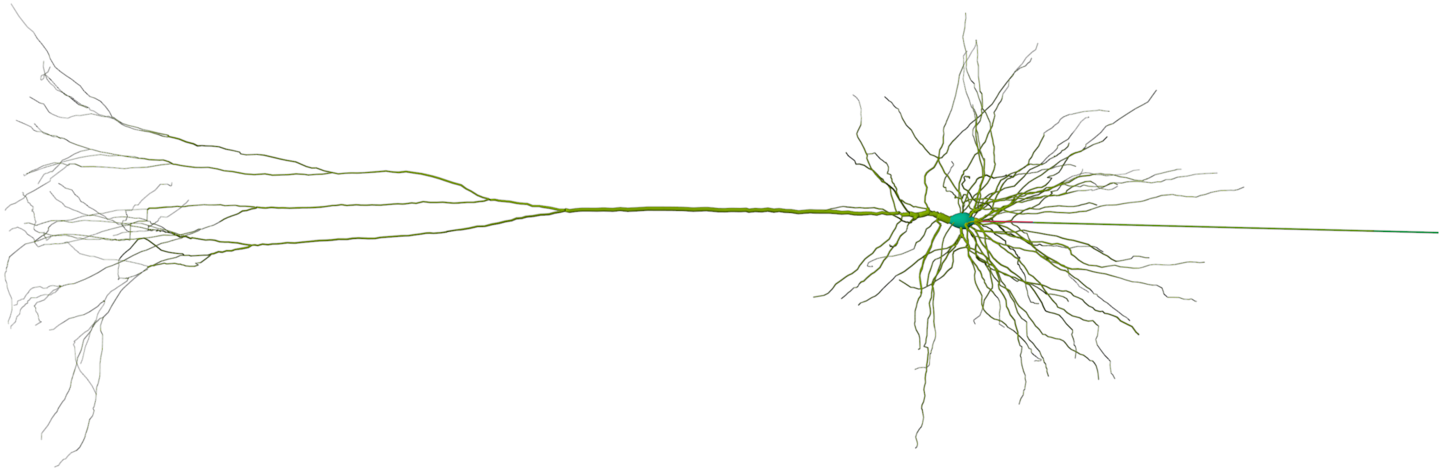

Fig A: **Geometry from the Hu model [15] (https://modeldb.science/123897): Layer 5 pyramidal neuron from cat visual cortex.** Somatodendritic morphology is digitally reconstructed from a real cell; hillock, AIS, and axon are added. Image created using blenderNEURON: blenderNEURON.org, blender.org.

The same reconstructed cell geometry was used in [15]. A cylindrical axon similar to that used in [15] was attached to the reconstructed soma by a 10μm long tapered hillock. The axon proper includes fifteen nodes of Ranvier (gray dots on the axon in Fig C) separated by myelinated 100μm internodes. The axon initial segment length was set to $\ell = 25.0$μm, consistent with [10]. Voltage-gated channels are present all over the cell. Channel densities and passive leaks are given as conductances. The local conductances vary with position —representative values have been tabulated in Section I.

In past modelling, there is sometimes a long section of bare axon following the AIS, or myelination may begin immediately at the end of the AIS. To ensure our results did not depend on this morphological feature, we ran simulations (axonal and somatic stimulation) with and without a 400μm section of bare axon separating the AIS from the first internode. The effects of Na_V distribution, summarized in Figures Fig H and Fig J, were qualitatively similar and similar in magnitude with and without the bare axon.

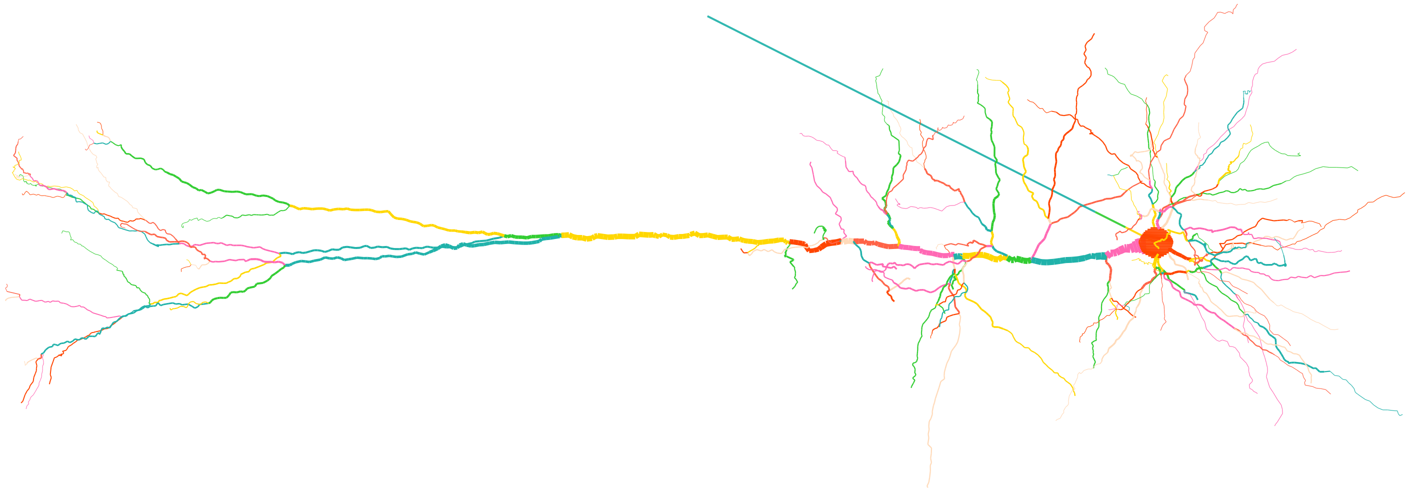

Fig B: **Morphology of the rat layer-5b neocortical pyramidal neuron in Hay et al. [29].** In the Hay model (https://modeldb.science/139653), the AIS consists of two straight NEURON Sections called "axon[0]" and "axon[1]", each 30µm in length, making the AIS 60µm long. The diagonal segment leading directly to the soma on the right is a 400µm long passive cable attached to the end of the AIS.

B Spikes and backpropagation in the Hu-based model

When pyramidal cells spike, the action potential can travel backward into the dendrites. This phenomenon is called backpropagation. For example, Fig C shows the response of the neuron following a somatic current pulse just above (left) and just below (right) the backpropagation threshold I_{BP}. Peak voltage is recorded across the entire cell model. The peak voltage at the tips of the dendrites is indicated in orange stars ★ and has a higher amplitude, consistent with the sealed-end effect. Backpropagation was deemed to have occurred if all apical dendritic tips exceeded -63.0mV (i.e. a depolarization of 7.0mV above V_{rest}) following stimulation.

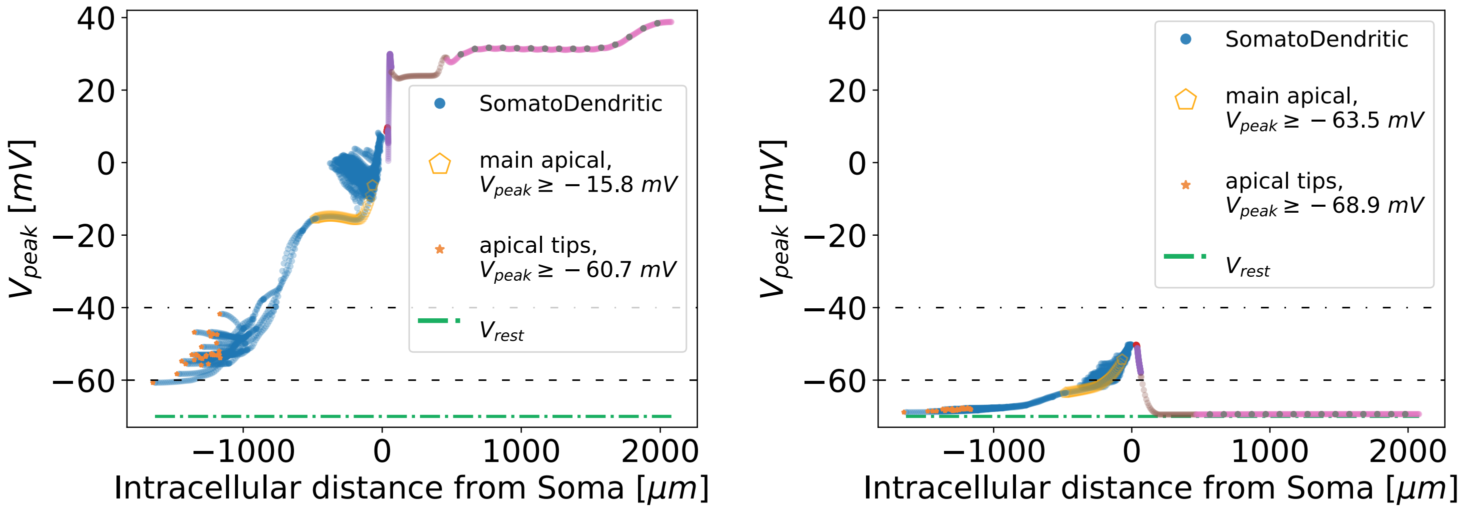

(i) Somatic stimulation, $I \gtrsim I_{BP} = I_{FP}$

(ii) Somatic stimulation, $I < I_{BP} = I_{FP}$

Fig C: **Backpropagation with somatic stimulation.** Each data point maps to a location in the reconstructed pyramidal neuron. On the abscissa, negative values indicate that a datapoint is located in the soma or dendrites, and positive values correspond to the hillock, AIS, and axon. (The correspondence between the abscissa and cell morphology is illustrated in Fig G.) ($x = 1, \kappa = 0.7$) This and similar figures are inspired by "Figure 4" of [12].

Note the qualitative change that occurs in the peak dendritic voltages of Fig Ci and Fig Di, when the cell is above threshold. A minuscule increase in the injected current amplitude has caused the entire main apical dendrite to depolarize well above -40mV, despite being mostly below -60mV when the current was slightly below this threshold. Note the attenuation in the peak voltage when comparing the basal dendrites to the apical tips. Also note the variety of different peak voltages in the dendrites, compared to the subthreshold condition. Our backpropagation criteria for the Hu-based model required this qualitative change since it was a robust feature of those simulations. Recall also that the purpose of the dendrites in

our modelling is to define a backpropagation threshold, not necessarily to mimic the detailed features of backpropagation. For our purposes, it is enough that the dendrites exhibit a threshold, and that threshold changes as a result of altering the distributions of $\mathrm{Na_V}$s in the AIS.

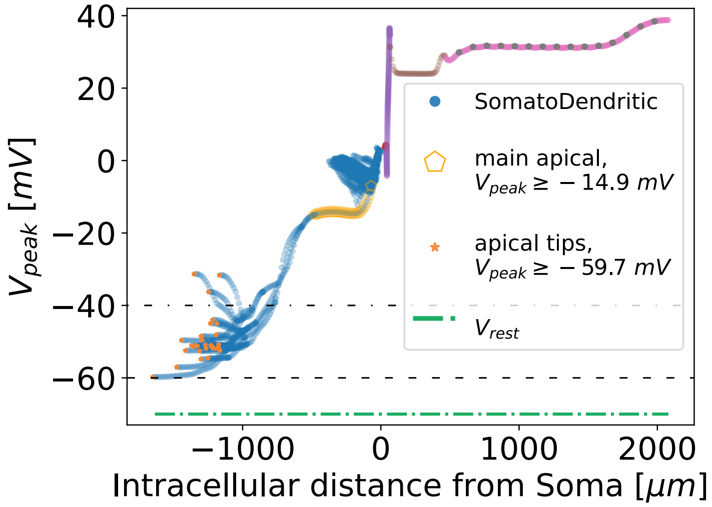

(i) Axonal stimulation, $I \gtrsim I_{BP} > I_{FP}$

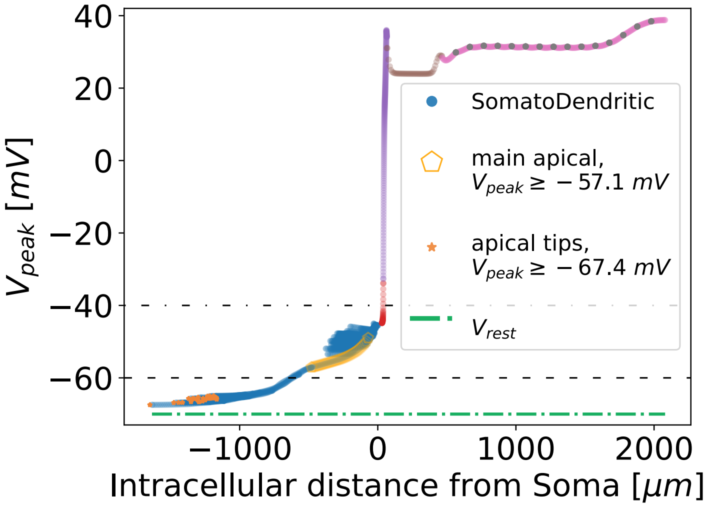

(ii) Axonal stimulation, $I_{BP} > I > I_{FP}$

Fig D: **Axonal stimulation in the Hu-based model: spikes with and without backpropagation —by our stringent criterion.** Current is injected just distal to the AIS. Each data point maps to a location in the reconstructed pyramidal neuron. (The correspondence between the abscissa and cell morphology is illustrated in Fig G.) In (i), an action potential (AP) has backpropagated. Note the variability of V_{peak} in the somatodendritic region of the neuron, and the significant attenuation as the wave travels into the distal dendrites. In (ii), an AP has occurred but the pattern of depolarization in the dendrites does not satisfy the backpropagation criterion we have used for this model. Note that the peak voltage in the soma and dendrites remains nearer to the resting potential $\approx -70\text{mV}$, never exceeding -40mV. Also note the lack of variability in the somatodendritic V_{peak}. The qualitative change in these two plots occurs sharply, just above $I_{BP} \approx 2.7\text{nA}$ $(x = 1, \kappa = 0.4)$. By some definitions, both of these scenarios would be considered backpropagation, since there is always a few mV of depolarization in the most distal dendrites. As this qualitative change is a robust feature of the Hu-based model, we have defined backpropagation in those simulations to require it. In defining a forward propagation threshold (I_{FP}), we call the scenario on the right, where an AP has been sent down the axon orthodromically from the stimulation site (i.e. to the right in this panel) “forward propagation” or “forwardprop” regardless of the amplitude of the somatodendritic depolarization. Antidromically stimulated APs that do not backpropagate have been observed in several neuron types [36, 37]. This and similar figures are inspired by “Figure 4” of [12].

C Spikes and backpropagation in the Hay-based model

The backpropagation criteria we used with the Hay model[29] are given in the caption of Fig E.

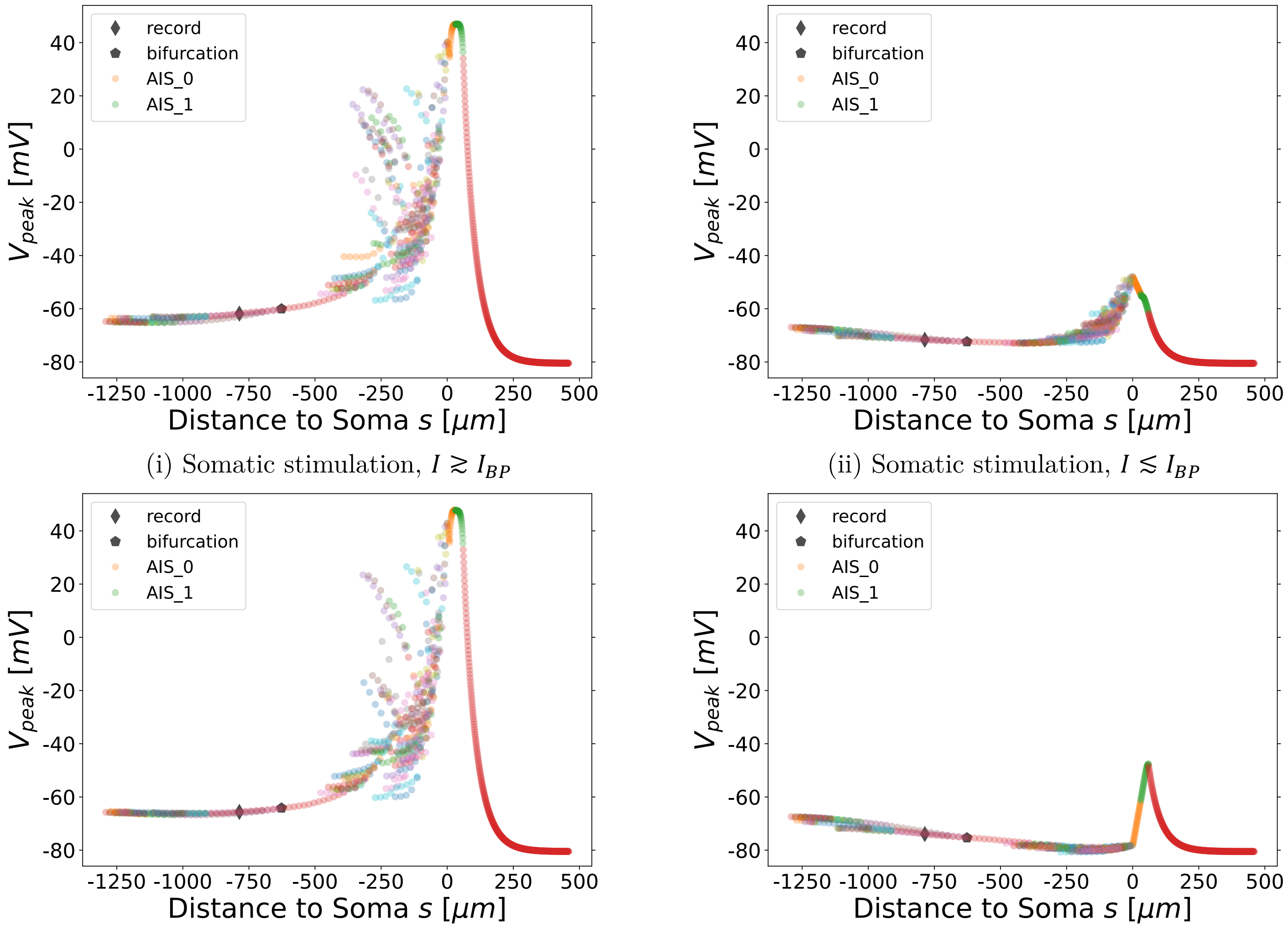

(i) Somatic stimulation, $I \gtrsim I_{BP}$

(ii) Somatic stimulation, $I \lesssim I_{BP}$

(iii) Axonal stimulation, $I \gtrsim I_{BP}$

(iv) Axonal stimulation, $I \lesssim I_{BP}$

Fig E: **Backpropagation criteria in the Hay-based model.** Each data point maps to a location in the reconstructed pyramidal neuron. On the abscissa, negative values indicate that a data point is located in the soma or dendrites, and positive values correspond to the AIS and passive cable. (The correspondence between the abscissa and cell morphology is illustrated in Fig G.) In (**i**) and (**iii**) backpropagation has occurred, whereas in (**ii**) and (**iv**), the stimulation is (just) below the BAP threshold. In the model, backpropagation was recorded either at the soma (neighbouring the left end of the section labelled "AIS_0", membrane potential exceeding 10mV), or in the apical dendrites (just distal to the main bifurcation of the main apical dendrite, membrane potential exceeding −70mV). The resting potential was ≅ −80.5mV at the soma, and ≅ −74.1mV at the apical recording site (see legend).

In Fig E, compare the depolarization of the distal AIS ('AIS_1' in the legend) in the Hay model [29] when current is injected somatically versus axonally, with the neuron just below I_{BP}: In the somatic case (Fig Eii) the distal AIS never reaches −60mV, while in the axonal case (Fig Eiv) it exceeds −50mV. Hay

et al. did not include an axon[29], and here the AIS is followed by a passive cable, rather than an excitable axon composed of myelinated internodes segmented by nodes of Ranvier. Hence a forward-propagation threshold I_{FP} is ill-defined in this model. Were the Hay model to include such an axon, the increased depolarization of the distal AIS in (Fig Eiv) compared to (Fig Eii) would be sufficient to cause an axonal AP in the former without doing so in the latter. Thus we argue, that the ability of the Hu-based model —with its excitable axon— to generate APs without meeting our criterion for backpropagation (Fig Dii), is an artifact of axonal stimulation under the conditions simulated here. Antidromically stimulated APs that do not backpropagate have been observed in several neuron types [36, 37].

C.1 Identical results with somatic criterion

In the main text (Fig 7) the dendritic backpropagation criterion was used, however, the somatic criterion produces the same result, as shown below. In Fig Fi and Fig Fii, backpropagation is registered if the somatic membrane potential exceeds 10.0mV. The threshold need not be this high, but it does not affect the results since the somatic depolarization is large.

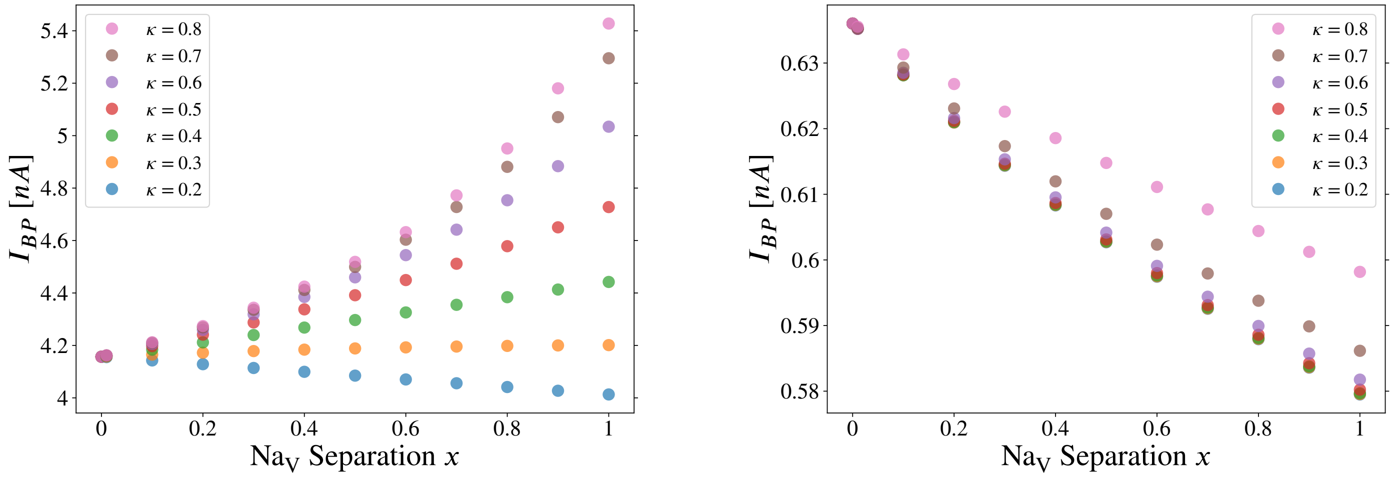

(i) Somatic stimulation (ii) Axonal stimulation

Fig F: **Backpropagation in the Hay model: Somatic backpropagation criterion.** To see the equivalency of the somatic criterion used here, with the apical dendritic backpropagation criterion used in the main text, compare with Fig 7. Here we plot the combined effect on the backpropagation threshold (I_{BP}, defined below) of varying crossover location (κ) and Na$_V$ separation (x) in the axon initial segment. Varying the separation parameter "x" from $x = 0$ to $x = 1$, the distribution of Na$_V$ channels goes from flat (homogeneous) to separated, the latter approximating the distribution observed in developing pyramidal neurons (see Fig 1A). Note that curves for all values of κ converge to a single point at $x = 0$, since κ can have no effect when the two Na$_V$ subtypes are uniformly distributed along the AIS. Somatic backpropagation criterion = 10.0mV —see caption of Fig E.

D Modified version of Hu-based model

In the version of our Hu-based model from the main text, the Na_V density in the dendrites was decreased by a factor of 10 from the original Hu model, to produce attenuation in the backpropagated action potential. Likewise, the somatic Na_V density was increased by a factor of 3. In the tuning presented here, the original somatic and dendritic Na_V densities of Hu et al. [15] are kept, and backpropagation is robust, without attenuation. A high amplitude, regenerative BAP infiltrates the entire dendritic tree.

We redefined the threshold criterion, such that backpropagation was deemed to have occurred if all apical dendritic tips exceeded $-10.0\mathrm{mV}$ following stimulation. The higher threshold value was appropriate to record backpropagation due to the robust BAP, which did not show attenuation (see Fig G). Although this tuning diverges from the qualitative features of BAPs in real pyramidal cells, at least for single action potentials [28, 29], the relationship between the backpropagation threshold and the Na_V distribution presented in Results was preserved again (see the figures below).

That is, despite the significant qualitative differences between the tuning of the Hu et al. [15] based model below (Fig G) which lacks BAP attenuation, the other Hu et al. based model above in the main text (Somatic stimulation, Axonal stimulation) which we modified for BAP attenuation, and the Hay et al. [29] based model above in Generalization to Hay-based model and modified Hu-based model, qualitative effects of modifying the Na_V distribution in the AIS are identical, and for somatic stimulation they are quantitatively similar as well. This can be clearly concluded by comparing Fig H, Fig 2, and Fig F.

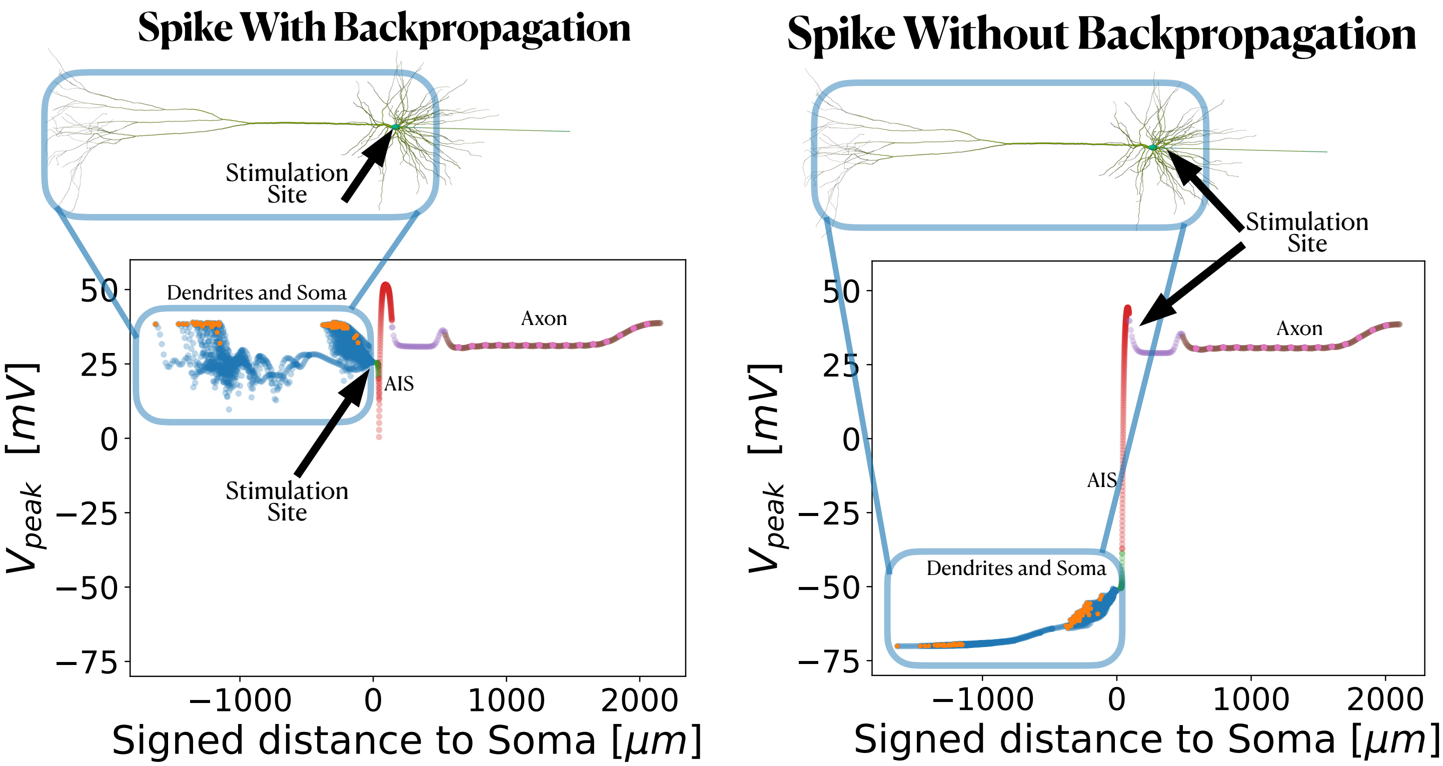

Fig G: Spikes with and without backpropagation: Each data point maps to a location in the reconstructed pyramidal neuron (compare with the cell morphology above the plot). On the abscissa, negative values indicate that a datapoint is located in the soma or dendrites, and positive values correspond to the hillock, AIS, and axon. Beginning on the right-hand side: an action potential (AP) has occurred following axonal stimulation (current injected just distal to the AIS). Note that the peak voltage in the soma and dendrites remains near the resting potential $\approx -70\text{mV}$, indicating that backpropagation did not occur. We call this scenario where an AP has been sent down the axon orthodromically from the stimulation site (i.e. to the right in this panel) “forward propagation” or “forwardprop” regardless of the amplitude of the somatodendritic depolarization. To the left is an AP that backpropagated: the entire cell spiked, in this case following somatic stimulation; backpropagation can also occur following axonal stimulation. The somatodendritic peak voltages are indicated by a blue box in each case. This and similar figures are inspired by “Figure 4” of [12].

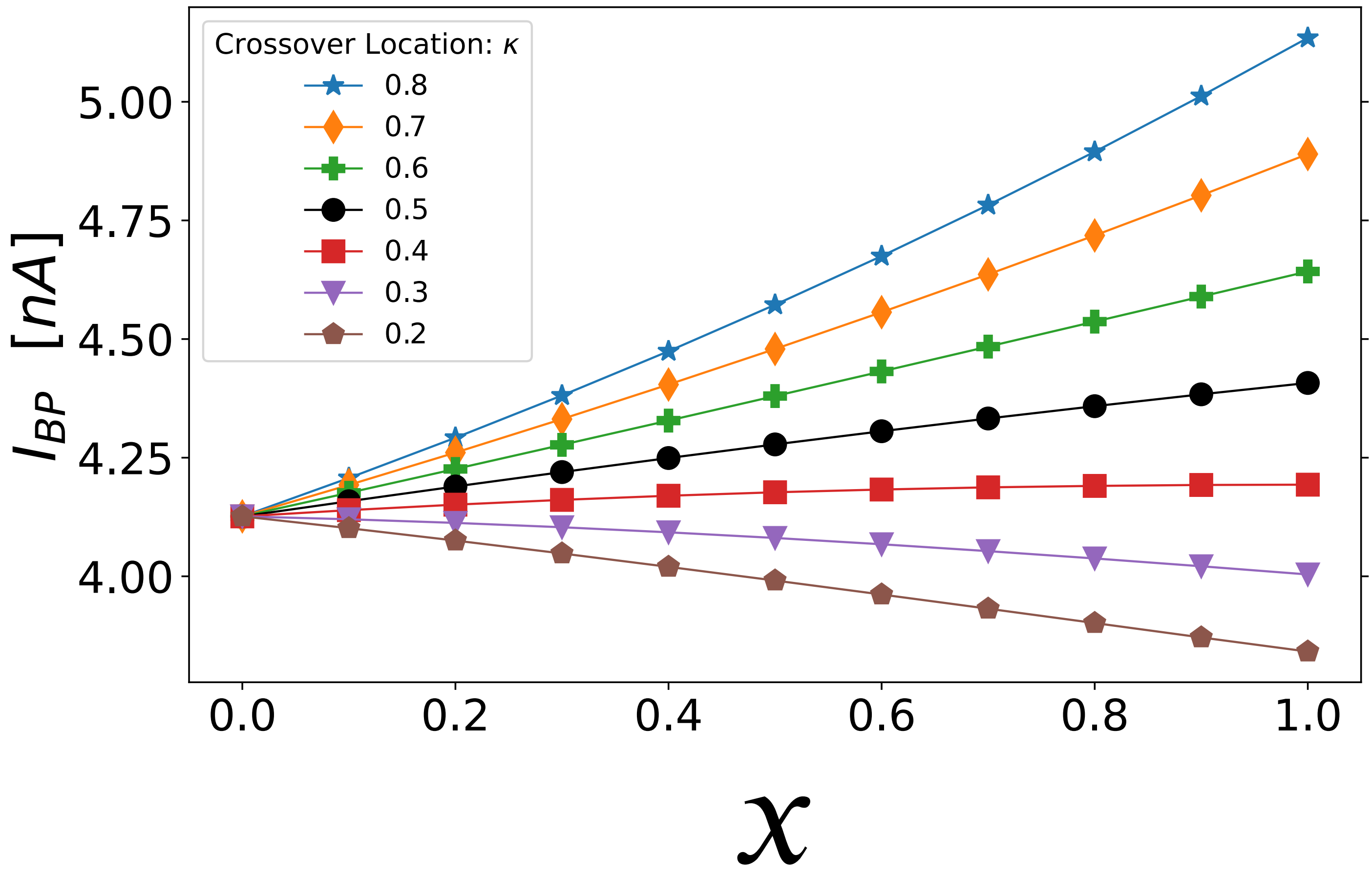

Fig H: Somatic Stimulation: Combined effect of varying crossover location (κ) and Na_V separation (x) in the axon initial segment. The threshold for forward AP propagation is the same as for backpropagation. Varying the separation parameter "x" from $x = 0$ to $x = 1$, the distribution of Na_V channels goes from flat (homogeneous) to separated, the latter approximating the distribution observed in developing pyramidal neurons (see Fig 1A). Note that curves for all values of κ converge to a single point at $x = 0$, since κ can have no effect when the two Na_V subtypes are uniformly distributed along the AIS. The lines have been drawn to guide the eye.

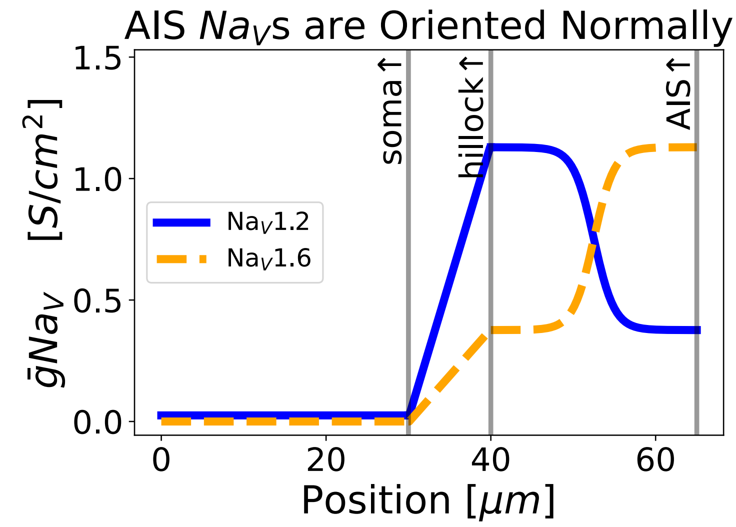

(i)

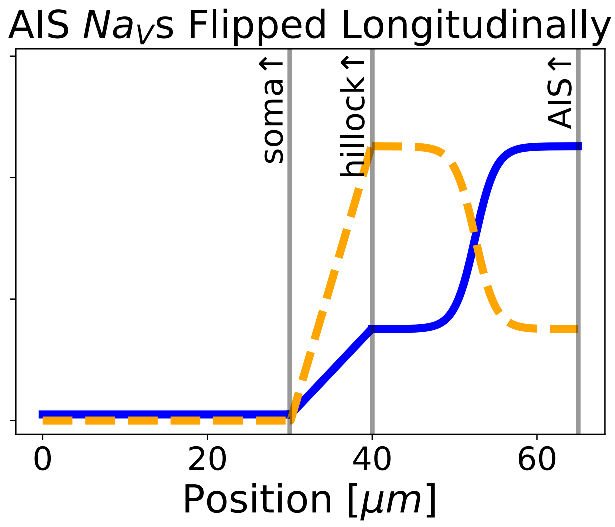

(ii)

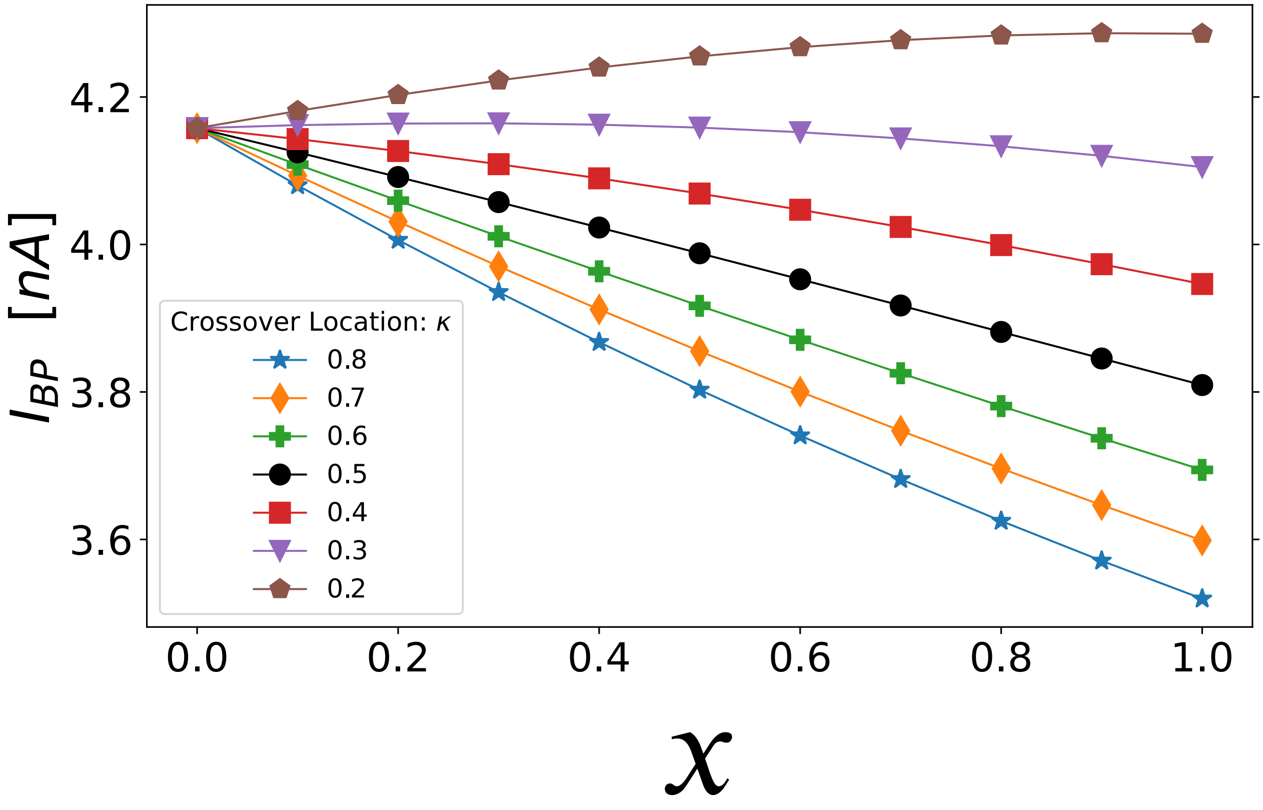

(iii)

Fig I: When the AIS Na_V distribution is flipped, setting $x = 1$ concentrates Na_V1.6 at the proximal AIS and Na_V1.2 at the distal AIS —the opposite of what is observed in many pyramidal cells [15, 16, 17]. (i) AIS with proper longitudinal placement of Na_Vs. (ii) AIS with a longitudinally flipped Na_V distribution. In both plots, $x = 0.5$ and $\kappa = 0.5$. (iii) Somatic stimulation with AIS Na_Vs flipped as in (ii): This result is close to a mirror image of Fig H. The lines have been drawn to guide the eye.

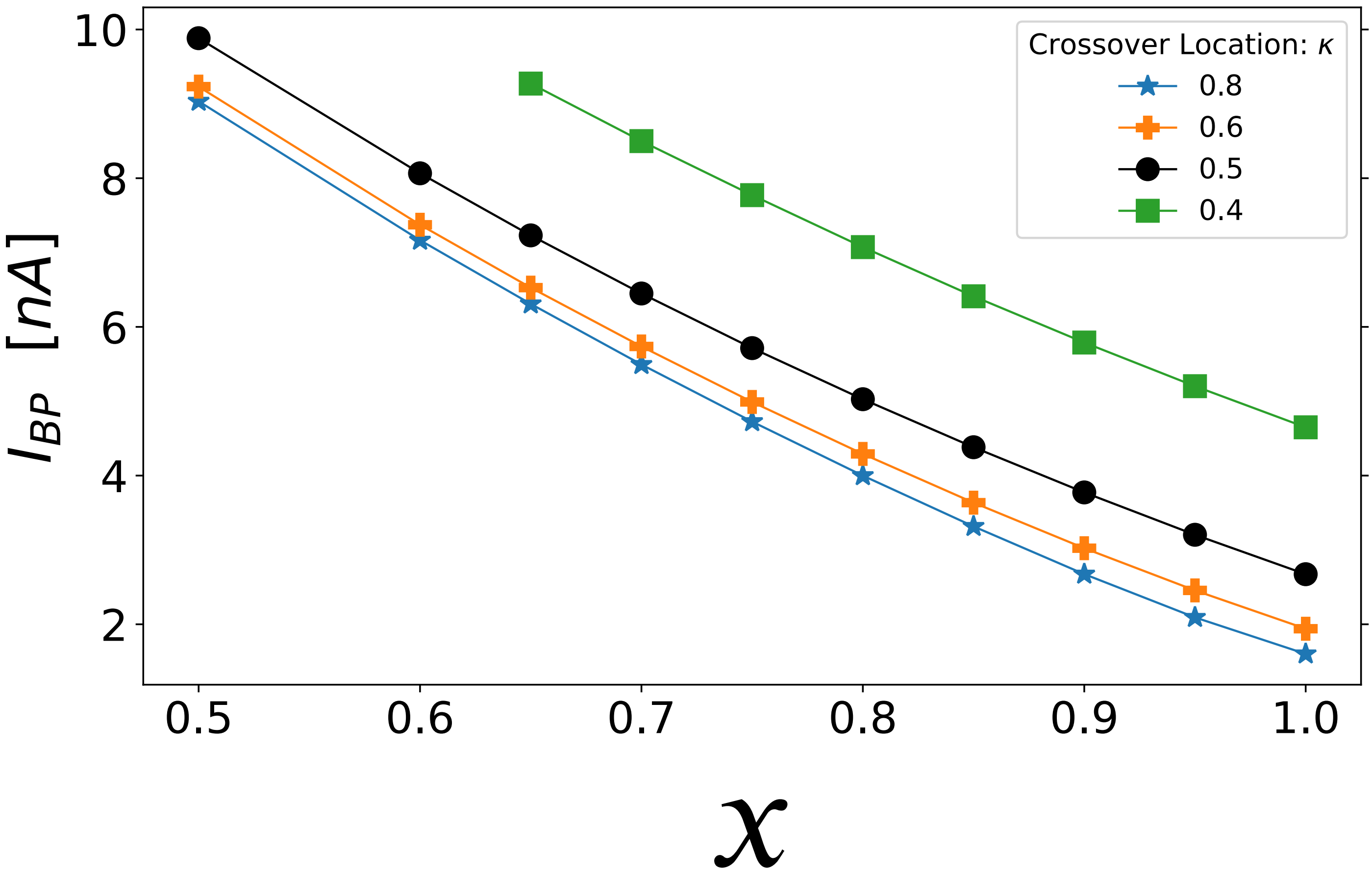

Fig J: Axonal Stimulation: Effect of varying crossover location (κ) and Na_V separation (x) in the AIS on the backpropagation threshold (see Fig 1). When computing the threshold, the stimulating current was limited to a maximum of $10\mathrm{nA}$, to prevent unphysiological local depolarization at the stimulation site. Due to the smaller diameter of the axon (relative to the soma), $10\mathrm{nA}$ is sufficient to depolarize the membrane potential to $\approx +80\mathrm{mV}$ at the stimulation site, whereas the resting potential is $V_{\mathrm{rest}} = -70\mathrm{mV}$. To achieve backpropagation within that constraint (following axonal stimulation), our model required some amount of proximal $Na_V1.2$, delivered through the combined effects of Na_V separation ($x \gtrsim 0.5$) and a sufficiently distal crossover position $\kappa \gtrsim 0.4$. Separating the two Na_V subtypes ($x \longrightarrow 1$) lowers the threshold, in agreement with the finding in [15] that proximal accumulation of $Na_V1.2$ promotes backpropagation, albeit due to different gating properties (Fig 6B). Increasing κ raises the proportion of $Na_V1.2$ (relative to $Na_V1.6$) in the AIS and lowers the backpropagation threshold as well. Threshold changes here are larger than for somatic stimulation (Fig H). The lines have been drawn to guide the eye.

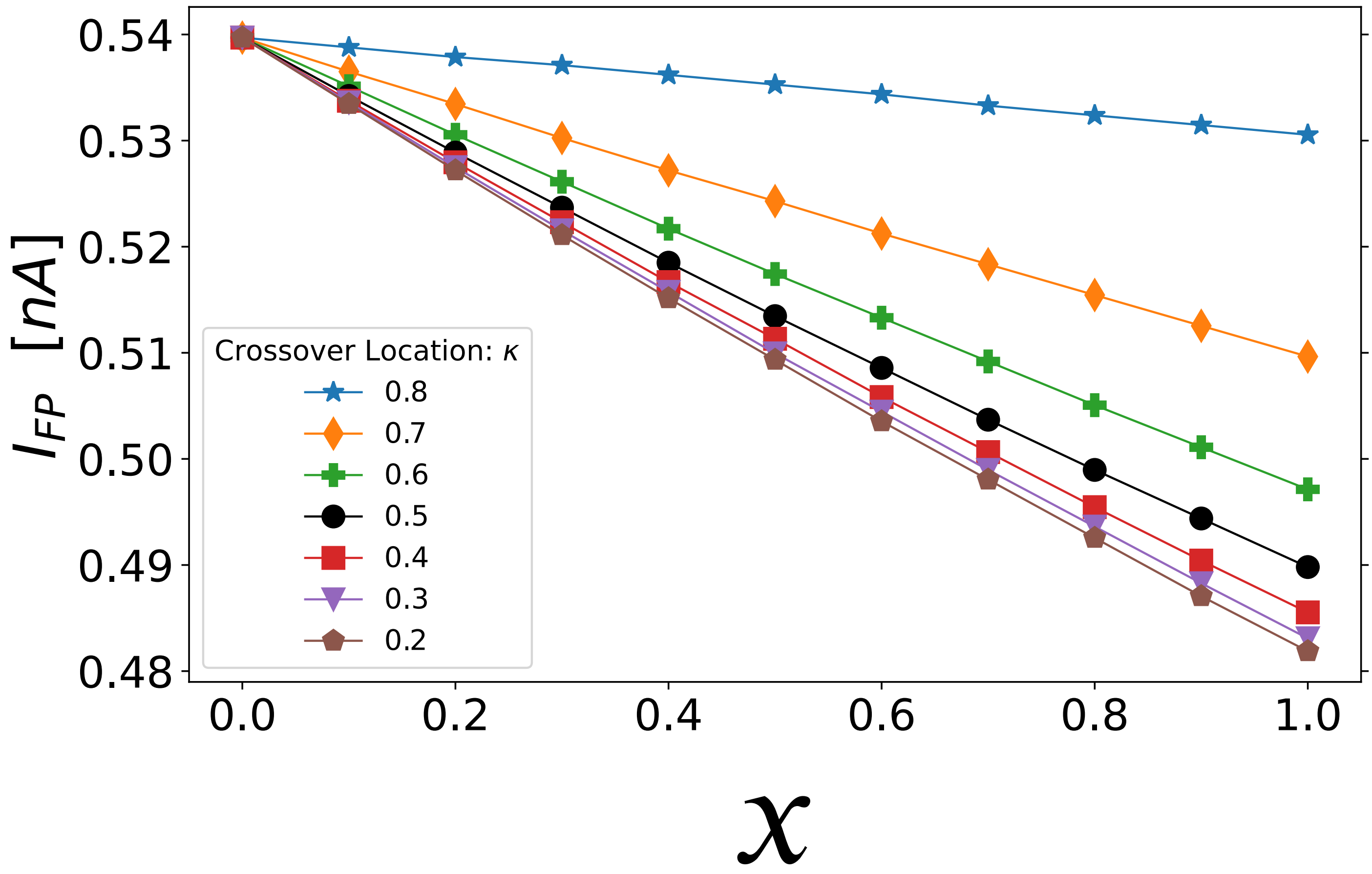

Fig K: Axonal Stimulation: Effect of x and κ on forward propagation threshold. The trend for all constant κ curves is that raising the proportion of total AIS $Na_V1.6$ (by reducing κ) or concentrating $Na_V1.6$ in the distal AIS (by increasing x) lowers the threshold to initiate forward propagating action potentials (see Fig 1). Note that this threshold current pulse is not sufficient to achieve backpropagation. The effect of Na_V separation is much smaller here than for the backpropagation threshold. The lines have been drawn to guide the eye.

D.1 Additional simulations

In the main text, we found that with a 25μm AIS, the slope of I_{BP} versus x for somatic stimulation became flat around $\kappa \approx 0.4$, which is 20μm away from the soma since $L_{\text{hillock}} = 10\mu\text{m}$ (Fig 2). In Fig L, with a 100μm AIS, the I_{BP} slope flattens around $\kappa \approx 0.1$, which again corresponds to a distance of roughly 20μm from the soma since the distance in μm to the crossover position is $\kappa \times \ell_{\text{AIS}}$. This suggests that the threshold-lowering effect of Na_V separation for small κ (Fig 2) results from the increased proximal density of Na_V1.6 when the crossover is brought near to the soma —and is not due to the proximal density of Na_V1.2, consistent with Fig M.

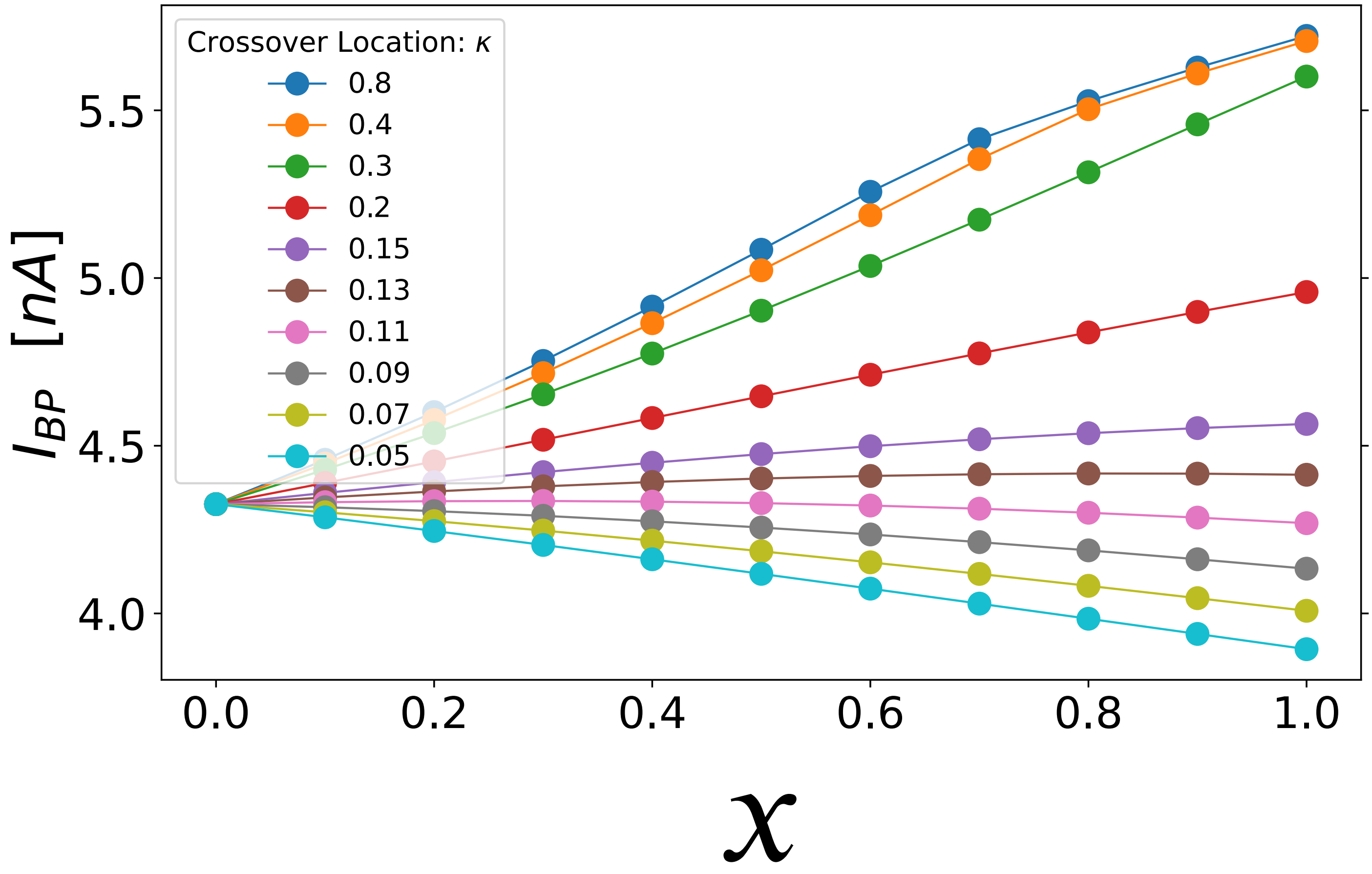

Fig L: Somatic Stimulation with AIS lengthened to 100μm (compare with Fig 2 in the main text where the AIS length was 25μm): Combined effect of varying crossover location (κ) and Na_V separation (x) in the axon initial segment. The distance in μm to the crossover position is $\kappa \times \ell_{\text{AIS}}$. The lines have been drawn to guide the eye.

Fig M demonstrates that increasing κ raises the backpropagation threshold when current is injected somatodendritically (orthodromic stimulation), even when the slopes in Fig 2 are negative. Increasing κ

means moving the Na_V crossover location away from the soma, which increases the proportion of $Na_V1.2$ (versus $Na_V1.6$) in the AIS; see Fig 1B.

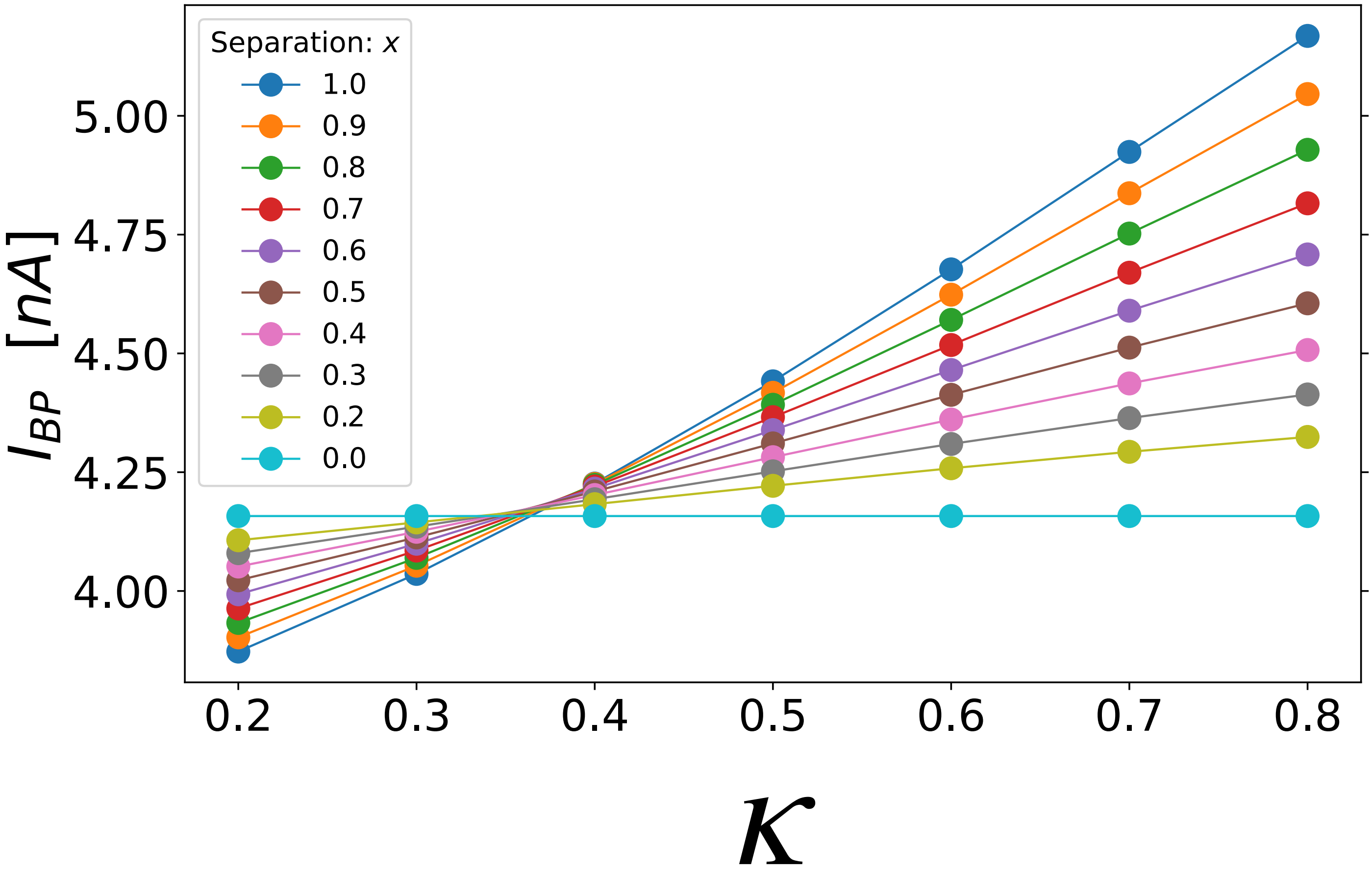

Fig M: Somatic Stimulation with nominal AIS length ($\ell_{\mathrm{AIS}} = 25.0\mu\mathrm{m}$). The combined effect of varying crossover location (κ) and Na_V separation (x) in the axon initial segment. Increasing κ raises the backpropagation threshold. Here the abscissa is the normalized crossover position κ, instead of Na_V separation (compare with Fig 2). In Fig 2, all curves converge at $x = 0$. Here, that intersection point is replaced by the $x = 0$ *line*. Notice that every $x > 0$ curve has a positive slope: the backpropagation threshold I_{BP} *increases* with κ. Increasing κ when $x > 0$ necessarily increases the ratio of $Na_V1.2$ conductance to $Na_V1.6$ conductance in the AIS (see Equation S2). It follows that concentrating $Na_V1.2$ in the proximal AIS raises the backpropagation threshold, for somatic stimulation. The lines have been drawn to guide the eye.

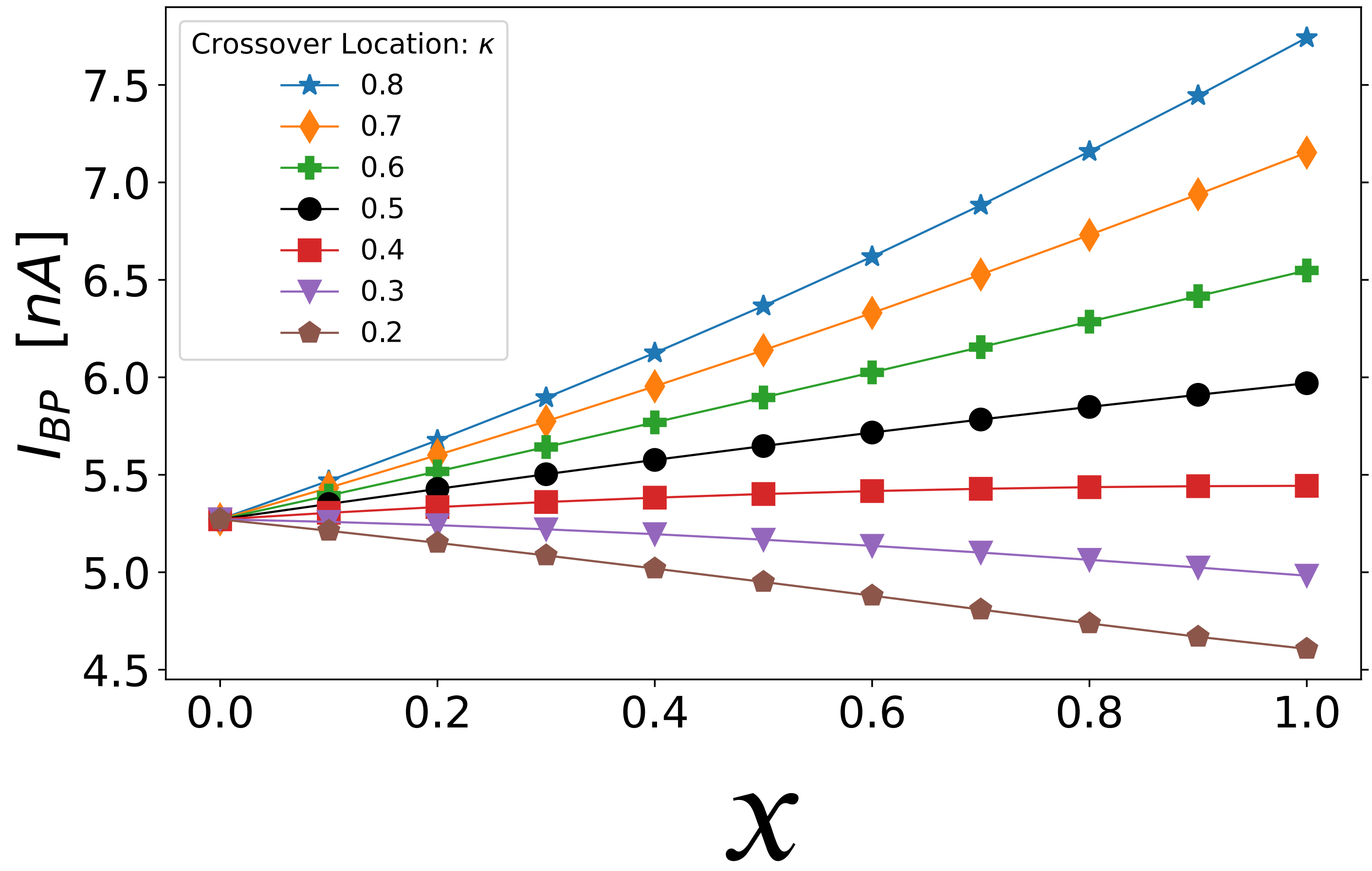

Fig N: Dendritic Stimulation with nominal AIS length ($\ell_{\text{AIS}} = 25.0\mu\text{m}$). Current injection at the main apical dendrite yields the same qualitative behaviour as for somatic stimulation. Compare with (Fig 2). Varying the separation parameter "x" from $x = 0$ to $x = 1$, the distribution of Na_V channels goes from flat (homogeneous) to separated, the latter approximating the distribution observed in developing pyramidal neurons (see Fig 1A). Note that curves for all values of κ converge to a single point at $x = 0$, since κ can have no effect when the two Na_V subtypes are uniformly distributed along the AIS. The lines have been drawn to guide the eye.

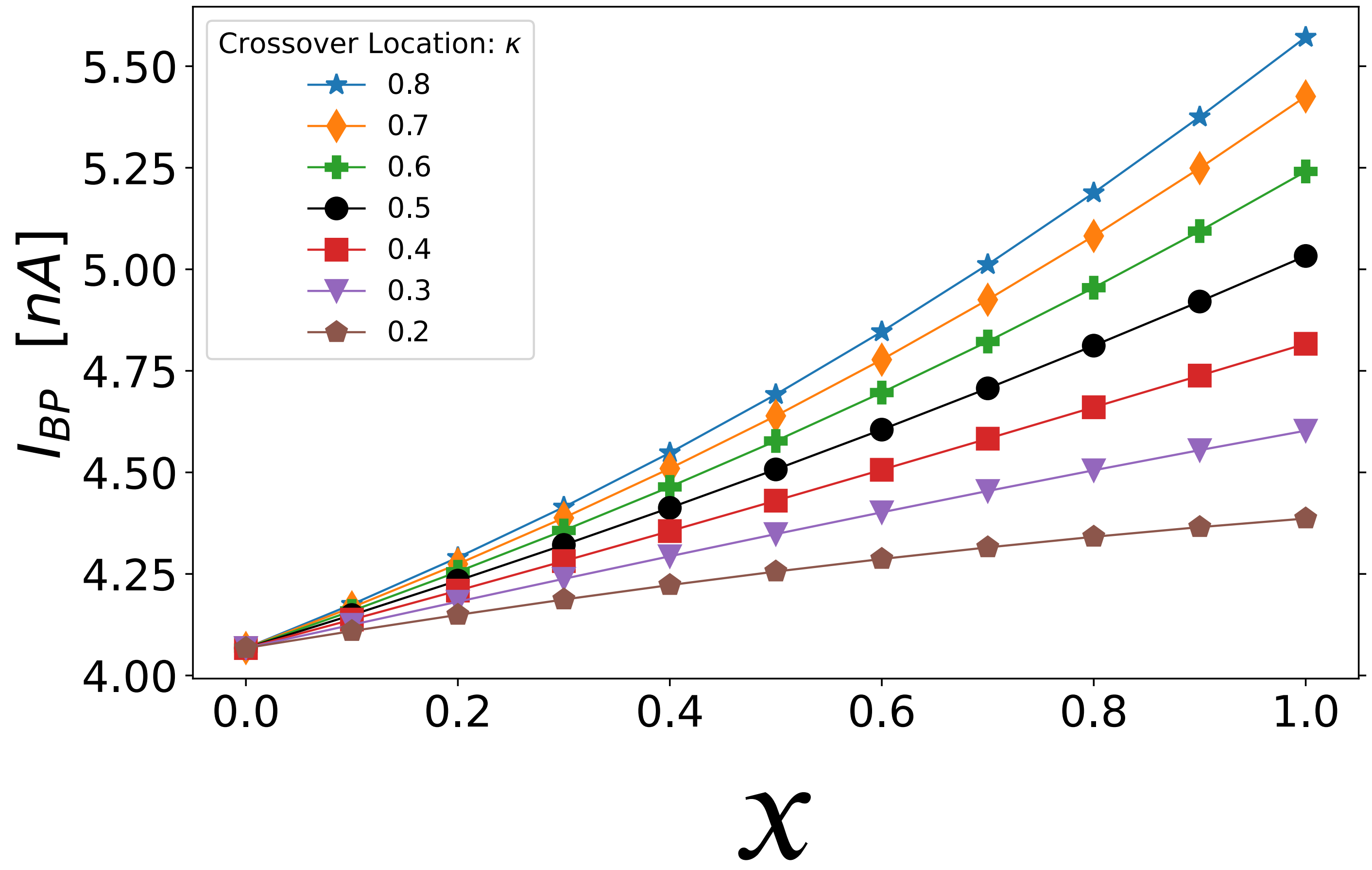

Fig O: Somatic Stimulation: lengthening the hillock to 30μm removes the negative slopes observed in Fig 2 and Fig L. In all other plots, we have used $L_{\text{hillock}} = 10\mu m$. AIS length is $\ell_{\text{AIS}} = 25.0\mu m$ as in all other plots unless indicated otherwise. The lines have been drawn to guide the eye.

E AIS - technical details

In all models that were used in this study, the total Na_V conductance of the AIS is proportional to the AIS length ℓ since its diameter is constant:

$$\bar{G}_{Na_V} = \bar{g}_{Na_V} \cdot (\text{Area of AIS}) \propto \bar{g}_{Na_V} \ell_{AIS}. \tag{S1}$$

As stated in the main text, the density profiles of $Na_V1.2$ and $Na_V1.6$ are left- and right-handed sigmoidal functions (respectively) of normalized length s along the AIS. The proximal end of the AIS is located at $s = 0$, and the distal end is located at $s = 1$. The channel densities are expressed as maximal conductances $\bar{g}_{Na_V1.2}$ (s) and $\bar{g}_{Na_V1.6}$ (s), where the total maximal Na_V conductance $\bar{g}_{Na_V}$ is constant along the AIS:

$$\bar{g}_{Na_V} = \bar{g}_{Na_V1.2}(s) + \bar{g}_{Na_V1.6}(s) = \mathit{const.} \tag{S2}$$

The density profiles are given by

$$\begin{cases} \bar{g}_{Na_V1.2}(s) & = \dfrac{\bar{g}_{Na_V}}{2}\Big(1 - x \cdot \tanh\big(\sigma(s-\kappa)\big)\Big), \\ \bar{g}_{Na_V1.6}(s) & = \bar{g}_{Na_V} - \bar{g}_{Na_V1.2}(s) \\ & = \dfrac{\bar{g}_{Na_V}}{2}\Big(1 + x \cdot \tanh\big(\sigma(s-\kappa)\big)\Big). \end{cases} \tag{S3}$$

We chose the hyperbolic tangent function $\tanh(s)$, but other sigmoidal functions would do just as well. The parameter x controls the separation of the Na_V distribution, that is, how separated the two Na_V subtypes are. When $x = 0$, the distribution becomes flat —$Na_V1.2$ and $Na_V1.6$ are mixed uniformly along the AIS. When $x = 1$, the proximal end of the AIS contains only $Na_V1.2$, and the distal end of the AIS contains only $Na_V1.6$. The parameter σ is the reciprocal of the 'transition width' of the AIS Na_V distributions normalized by the AIS length. In all simulations shown here, $\sigma = 10.0$.

The contribution of $Na_V1.2$ to the total voltage-gated sodium conductance of the AIS (Equation S1) is

$$\bar{G}_{Na_V1.2} \propto \int_0^{\ell_{AIS}} d\ell' \, \bar{g}_{Na_V1.2}(\ell') = \ell_{AIS} \int_0^1 ds \, \frac{\bar{g}_{Na_V}}{2}\Big(1 - x \cdot \tanh\big(\sigma(s-\kappa)\big)\Big), \tag{S4}$$

$$\implies \bar{G}_{\mathrm{Na_V}1.2} \propto 1 - x \cdot \underbrace{\left(\frac{\ln\Big(\cosh(\sigma) - \sinh(\sigma)\tanh(\sigma\kappa)\Big)}{\sigma} \right)}_{\text{“}M\text{”}}. \tag{S5}$$

The root of the term labeled “M” in Equation S5 is $\kappa = \frac{1}{2}$, and the slope of M is negative: $M(\kappa < 0.5) > 0$, $M(\kappa > 0.5) < 0$. Since $M(\kappa = 0.5) = 0$, the derivative of $\bar{G}_{\mathrm{Na_V}1.2}$ (and $\bar{G}_{\mathrm{Na_V}1.6}$) with respect to x is zero when the crossover is located in the middle of the AIS, which is the standard configuration for varying $\mathrm{Na_V}$ separation.

We call $\kappa = 0.5$ standard because, in this configuration, the effects on the backpropagation threshold due to varying x can not be due to changes in the total conductance of $\mathrm{Na_V}1.6$ or $\mathrm{Na_V}1.2$ in the AIS. In other words, the results of sweeping x from 0 to 1 with κ fixed at 0.5 in Fig 4, Fig 5, Fig 3, Fig 7, and Fig 2 are purely due to mixing and separating $\mathrm{Na_V}1.6$ from $\mathrm{Na_V}1.2$ in the AIS.

Since by definition $0 \leqslant \kappa \leqslant 1$ and $\sigma > 0$, the partial derivative of M with respect to κ is negative. It follows from Equation S5 that $\bar{G}_{\mathrm{Na_V}1.2}$ increases as the crossover position is moved distally. Likewise $\bar{G}_{\mathrm{Na_V}1.6}$ decreases with increasing κ.

F Voltage-gated channels

The voltage-gated sodium and potassium conductances $g_{\mathrm{Na_V}}(V,t)$ and $g_{\mathrm{K_V}}(V,t)$ in Equation 16 are modeled using HH-style kinetics [40] fitted to mammalian pyramidal cell data by [12], and then further adapted by [15] to include two $\mathrm{Na_V}$ variants (Equation S7). In the Hodgkin-Huxley model, the current density I_Z of ion species “Z” through voltage-gated channels of a given type is

$$I_{\mathrm{Z_V}} = \bar{g}_{\mathrm{Z_V}} m^p h^q (V - E_{\mathrm{Z}}), \tag{S6}$$

where $\bar{g}$ is the maximal conductance, $m(V,t)$ is the probability for an *activation* gate to be open, p is the number of *activation* gates per channel, $h(V,t)$ is the *availability* (probability that the channel is *not inactivated*), and q is the number of inactivation gates per channel. $\mathrm{Na_V}$ channels are modeled as having three *activation* gates ($p = 3$) and a single inactivation gate ($q = 1$) so that $g_{\mathrm{Na_V}} \propto m^3 h$. Likewise, $\mathrm{K_V}$ channels have a single *activation* gate and no inactivation ($g_{\mathrm{K_V}} \propto n$).

The gating variables m, h, and n evolve according to Equations 7, 8, and 9. Since the cell features two

Na_V subtypes ($Na_V1.2$ and $Na_V1.6$), the model computes two sets of sodium *activation* and *availability* variables. The current density I_{Na} through the Na_V channels is then

$$I_{Na_V} = \Big(\underbrace{\bar{g}_{Na_V1.2}(m^{Na_V1.2})^3 h^{Na_V1.2}}_{g_{Na_V1.2}} + \underbrace{\bar{g}_{Na_V1.6}(m^{Na_V1.6})^3 h^{Na_V1.6}}_{g_{Na_V1.6}} \Big)(V - E_{Na^+}), \quad \text{(S7)}$$

where $g_{Na_V1.2} + g_{Na_V1.6} = g_{Na_V}$ in Equation 16.

Fig P plots the voltage-dependent kinetics of $m^{Na_V1.2}$, $h^{Na_V1.2}$, $m^{Na_V1.6}$ and $h^{Na_V1.6}$. The steady-state *activation* and *availability* functions and their voltage-sensitive time constants were implemented using the parameters provided in [15] and in model code published by [26]. We further modified the channel model code to allow shift-clamping; see Modifying the *right-shift* of $Na_V1.2$ gating properties in the AIS and Shift-Clamping and the Hodgkin-Huxley model.

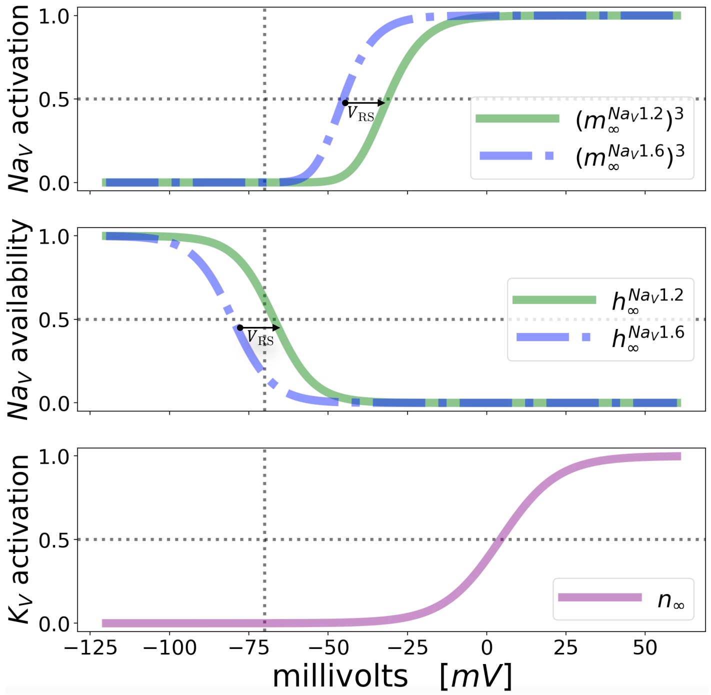

(i) Gating variables' steady-states

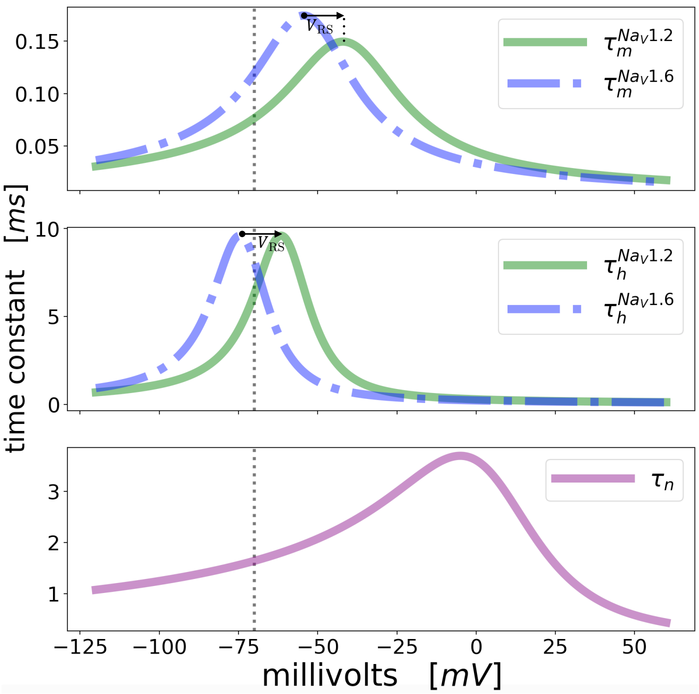

(ii) And their voltage-sensitive time constants

Fig P: Gating properties of the voltage-gated sodium and potassium channels that are implemented in Hu-based model. Dotted vertical lines indicate the resting potential V_{rest}. (i) Steady-state *activation* and *availability* curves for $Na_V1.2$ and $Na_V1.6$, and steady-state K_V *activation*. (ii) Voltage-sensitive time constants of $Na_V1.2$, $Na_V1.6$, and K_V. $Na_V1.2$ is *right-shifted* by an amount V_{RS} relative to $Na_V1.6$ (defined in Section F.1). In this model, V_{RS} = 13.0mV; also called the 'nominal *right-shift*' of $Na_V1.2$. V_{RS} is **indicated by arrows** (small •⟶) in the plots of Na_V steady-states (i) and time constants (ii) above.

V_{RS} is indicated by arrows (small •⟶) in the plots of Na_V steady-states (Fig Pi) and time constants

(Fig Pii).

The *right-shift* of $\mathrm{Na_V}1.2$ (defined mathematically in Section F.1) is easiest to observe in the top two plots of Fig Pi. V_{RS} is indicated by arrows (small $\bullet\!\!\longrightarrow$). Steady-state *activation* $\left(m_\infty^{\mathrm{Na_V}1.2}(V)\right)^3$ and *availability* $h_\infty^{\mathrm{Na_V}1.2}(V)$ curves of $\mathrm{Na_V}1.2$ are plotted as solid green lines, and dashed blue lines are the corresponding curves for $\mathrm{Na_V}1.6$. The voltage separating the two $\mathrm{Na_V}$ subtypes' *activation* curves is approximately the nominal *right-shift*, V_{RS}; however, it varies with position, since the gating variables of $\mathrm{Na_V}$ *activation* ($m^{\mathrm{Na_V}1.2}$, $m^{\mathrm{Na_V}1.6}$) have different slopes [15]. However, the *availability* curves are easier to compare: in this model, $h_\infty^{\mathrm{Na_V}1.2}(V)$ is shifted exactly $V_{\mathrm{RS}} = 13.0\mathrm{mV}$ to the right of $h_\infty^{\mathrm{Na_V}1.6}(V)$. The *right-shift* of $\mathrm{Na_V}1.2$ is also visible in the voltage-sensitive time constants (Fig Pii).

F.1 Defining V_{RS}: the *right-shift* of $\mathrm{Na_V}1.2$

V_{RS} is a parameter in this model representing the experimentally measured depolarizing shift in the voltage dependence of $\mathrm{Na_V}1.2$ *activation* and inactivation kinetics, relative to $\mathrm{Na_V}1.6$ kinetics. Because V_{RS} is empirical, it is fixed in this paper.

We define the $\mathrm{Na_V}1.6$ half-*activation* voltage $V_{1/2}^{\mathrm{Na_V}1.6}$ as the voltage at which a single $\mathrm{Na_V}1.6$ *activation* gate (randomly selected from an ensemble of gates held at $V = V_{1/2}^{\mathrm{Na_V}1.6}$) has a 50% chance of being in the open state:

$$m_\infty^{\mathrm{Na_V}1.6}(V_{1/2}^{\mathrm{Na_V}1.6}) \stackrel{\text{def}}{=} \frac{1}{2}.$$

Although the kinetics of $\mathrm{Na_V}1.2$ differ from $\mathrm{Na_V}1.6$ kinetics in ways *other than right-shift*, we can now use $V_{1/2}^{\mathrm{Na_V}1.6}$ to define V_{RS} as the voltage that satisfies

$$\boxed{m_\infty^{\mathrm{Na_V}1.2}(V_{1/2}^{\mathrm{Na_V}1.6} + V_{\mathrm{RS}}) = m_\infty^{\mathrm{Na_V}1.6}(V_{1/2}^{\mathrm{Na_V}1.6}) = \frac{1}{2}}. \tag{S8}$$

Note that $V_{1/2}^{\mathrm{Na_V}1.6}$ and V_{RS} are unique since m_∞ is monotonically increasing. Equation S8 also contains the half-*activation* voltage for $\mathrm{Na_V}1.2$,

$$\left(V_{1/2}^{\mathrm{Na_V}1.6} + V_{\mathrm{RS}}\right) = V_{1/2}^{\mathrm{Na_V}1.2}, \tag{S9}$$

which is depolarized or "*right-shifted*" by an amount V_{RS} relative to $\mathrm{Na_V}1.6$ (see Fig P).

To simulate alterations to $\mathrm{Na_V}1.2$ *right-shift* in our shift-clamping method, we use another parameter

called ΔV_{RS}, which is not based on experiment. Although the $\mathrm{Na_V}$1.2 *right-shift* is not a high precision measurement ($V_{\mathrm{RS}} \sim 10-15\mathrm{mV}$), the *parameter* V_{RS} is kept fixed in our model for conceptual purposes: we find it helpful to distinguish empirical parameters (like V_{RS}) from exploratory parameters that intentionally deviate from experiment (like ΔV_{RS}). In fact, we use ΔV_{RS} to selectively change the model's $\mathrm{Na_V}$ kinetics to explain the effects of $\mathrm{Na_V}$ distribution on excitability in terms of the lengthwise distribution of gating properties (see Modifying the *right-shift* of $\mathrm{Na_V}$1.2 gating properties in the AIS and Shift-Clamping and the Hodgkin-Huxley model).

F.2 Notation: V_{RS}, ΔV_{RS}

In our notation, V_{RS} is *not written explicitly* in the argument of $\mathrm{Na_V}$1.2 gating variables or their time constants. Instead, we write

$$\tau_h^{\mathrm{Na_V 1.6}}(V),\ \tau_h^{\mathrm{Na_V 1.2}}(V),\ m_\infty^{\mathrm{Na_V 1.6}}(V),\ m_\infty^{\mathrm{Na_V 1.2}}(V),\ \text{et cetera,}$$

and let the superscript "$\mathrm{Na_V 1.2}$" indicate that $\tau_h^{\mathrm{Na_V 1.2}}$ is *right-shifted* relative to $\tau_h^{\mathrm{Na_V 1.6}}$, from the fact that $\mathrm{Na_V}$1.2 channels are *right-shifted* in this model.

However, the parameter ΔV_{RS} *is* written explicitly in the argument when we model the effects of modifying the *right-shift* (Fig 6: shift-clamping). For example, when applying $\Delta V_{\mathrm{RS}} \neq 0$ to the *selected* gating properties $\tau_h^{\mathrm{Na_V 1.2}}$ and $m_\infty^{\mathrm{Na_V 1.2}}$, we would write

$$\begin{cases} \tau_h^{\mathrm{Na_V 1.2}} = \tau_h^{\mathrm{Na_V 1.2}}(V - \Delta V_{\mathrm{RS}}) \\ h_\infty^{\mathrm{Na_V 1.2}} = h_\infty^{\mathrm{Na_V 1.2}}(V) \\ \tau_m^{\mathrm{Na_V 1.2}} = \tau_m^{\mathrm{Na_V 1.2}}(V) \\ m_\infty^{\mathrm{Na_V 1.2}} = m_\infty^{\mathrm{Na_V 1.2}}(V - \Delta V_{\mathrm{RS}}). \end{cases} \tag{S10}$$

Using this notation, positive values of ΔV_{RS} will shift kinetic curves (gating properties) to the right in Fig P (depolarizing shift), and negative ΔV_{RS} produces a hyperpolarizing shift.

F.3 Space plots of $\mathrm{Na_V}$ kinetics along the AIS — steady-state

The distribution of $\mathrm{Na_V}1.2$ and $\mathrm{Na_V}1.6$ in the AIS creates a lengthwise distribution of gating properties. Proximal $\mathrm{Na_V}1.2$ increases local steady-state *availability*, owing to these channels' *right-shift* (V_{RS}). In Fig Qi we visualize this effect using $\mathcal{H}(s)$: the net *availability* of $\mathrm{Na_V}$s ($\mathcal{H}$) as a function of position (s), computed by weighting $h^{\mathrm{Na_V}1.2}$ and $h^{\mathrm{Na_V}1.6}$ according to their respective local $\mathrm{Na_V}$ channel densities:

$$\mathcal{H}(s,t) = \frac{\bar{g}_{\mathrm{Na_V}1.2}(s)h^{\mathrm{Na_V}1.2}(s,t) + \bar{g}_{\mathrm{Na_V}1.6}(s)h^{\mathrm{Na_V}1.6}(s,t)}{\bar{g}_{\mathrm{Na_V}1.2}(s) + \bar{g}_{\mathrm{Na_V}1.6}(s)}. \tag{S11}$$

An effective local $\mathrm{Na_V}$ time constant of *availability* ($\boldsymbol{\mathcal{T}}$) can be computed from $\tau_h^{\mathrm{Na_V}1.2}$ and $\tau_h^{\mathrm{Na_V}1.6}$ as

$$\boldsymbol{\mathcal{T}}(s,t) = \frac{\bar{g}_{\mathrm{Na_V}1.2}(s)\tau_h^{\mathrm{Na_V}1.2}(s,t) + \bar{g}_{\mathrm{Na_V}1.6}(s)\tau_h^{\mathrm{Na_V}1.6}(s,t)}{\bar{g}_{\mathrm{Na_V}1.2}(s) + \bar{g}_{\mathrm{Na_V}1.6}(s)}. \tag{S12}$$

Above, we have abbreviated

$$f(V(s,t),t) = f(s,t) \text{ for } f = h^{\mathrm{Na_V}1.2}, h^{\mathrm{Na_V}1.6}, \tau_h^{\mathrm{Na_V}1.2}, \tau_h^{\mathrm{Na_V}1.6}. \tag{S13}$$

In Fig Qii we have selectively disabled the *right-shift* of $\tau_h^{\mathrm{Na_V}1.2}$ by setting $\Delta V_{\mathrm{RS}} = -V_{\mathrm{RS}} = -13.0\mathrm{mV}$ in Equation 3, which leaves the *right-shift* of steady-state *availability* unchanged.

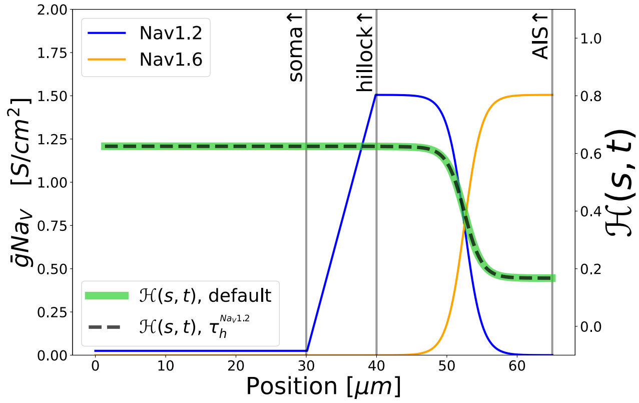

(i) Solid green curve: nominal $\mathrm{Na_V}1.2$ *right-shift*. Travelling from left to right across the AIS, $\mathrm{Na_V}1.6$ overtakes $\mathrm{Na_V}1.2$ as a share of the total local $\mathrm{Na_V}$ density. The accompanying change in $\mathrm{Na_V}$ *right-shift* causes $\mathcal{H}(s)$ to drop from $\approx$ 0.6 at the soma and proximal AIS, down to $\approx$ 0.2 at the distal AIS and axon. Dashed black curve: the *right-shift* of $\tau_h^{\mathrm{Na_V}1.2}$ has been selectively disabled without altering $h_\infty^{\mathrm{Na_V}1.2}$, and thereby $\mathcal{H}$ is also unaffected. See (ii) to the right as well as Equation 3.

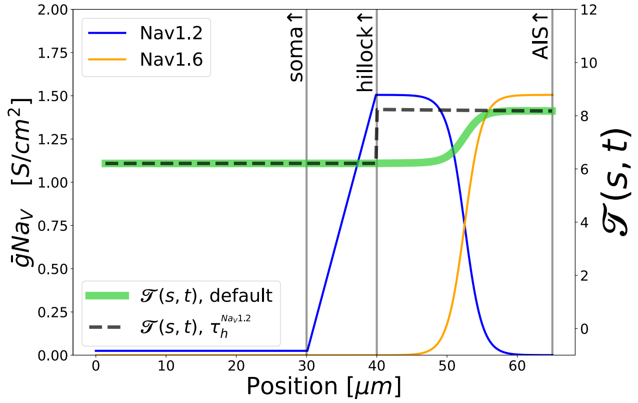

(ii) Solid green curve: nominal $\mathrm{Na_V}1.2$ *right-shift*. Where $\mathrm{Na_V}1.6$ overtakes $\mathrm{Na_V}1.2$ as a share of the total local $\mathrm{Na_V}$ density, $\mathcal{T}(s)$ increases from $\approx$ 6ms to $\approx$ 8ms at V_{rest}. Dashed black curve: *right-shift* of $\tau_h^{\mathrm{Na_V}1.2}$ selectively disabled by setting $\Delta V_{\mathrm{RS}} = -V_{\mathrm{RS}}$ in Equation 3. The effective time constant of $\mathrm{Na_V}$ *availability* $\mathcal{T}(s)$ is now uniform along the AIS. The abrupt transition in $\mathcal{T}$ at the AIS-hillock interface —instead of the smooth transition along the AIS in the green curve— is due to the *right-shift* of $\tau_h^{\mathrm{Na_V}1.2}(V)$ being selectively disabled —see Equation S12. The *right-shift* of $h_\infty^{\mathrm{Na_V}1.2}(V)$ has not been altered (see Equation S11), and the corresponding $\mathcal{H}$ (dashed black) curve in (i) is unchanged —hence the term "selective".

Fig Q: The effect of $\mathrm{Na_V}1.2$ *right-shift* on local $\mathrm{Na_V}$ *availability* along the cell is visible in space plots of steady-state total *availability* $\mathcal{H}(s)$ (i) and total time constant $\mathcal{T}(s)$ (ii) at $V = V_{\mathrm{rest}}$. See Equation S11 and Equation S12, respectively.

G Transformed backpropagation threshold data

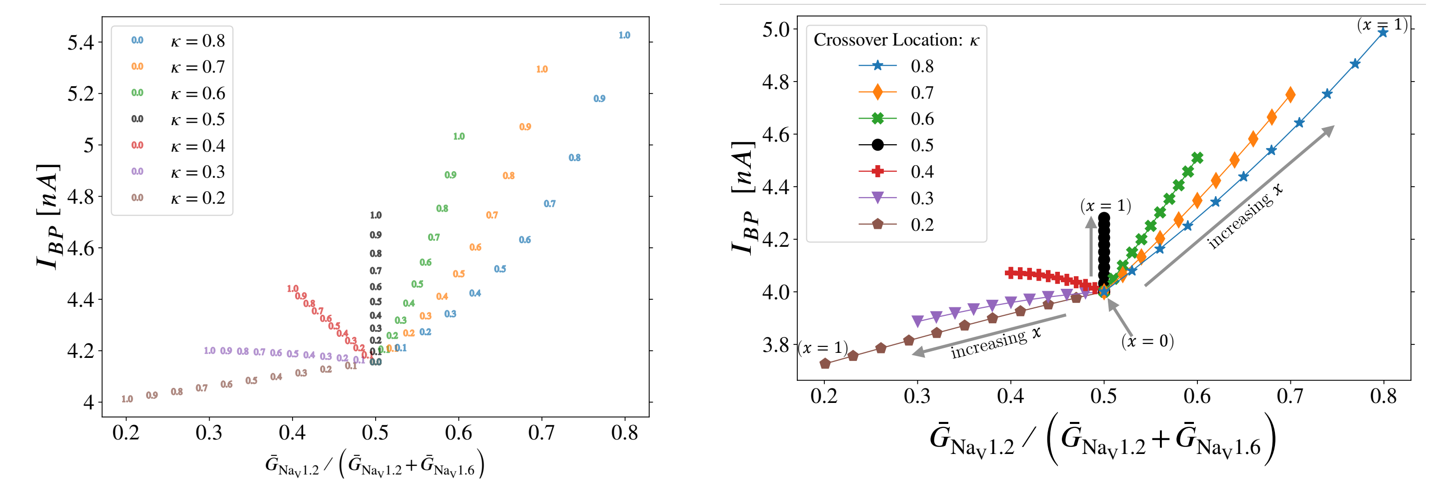

Fig R: **Backpropagation threshold plotted against the proportion of total AIS $\mathbf{Na_V}$ conductance allocated to $\mathbf{Na_V}$1.2. (Somatic Stimulation.)** The data presented here are the I_{BP} measurements with somatic stimulation in the Hay-based model (left, see Fig 7A) and Hu-based model (right, see Fig 2) from the main text, transformed as follows: On the abscissa we replaced x with the ratio of $\mathrm{Na_V}$1.2 channels —summed across all compartments in the AIS— to all $\mathrm{Na_V}$ channels in the AIS $\left(\frac{\sum_{\text{AIS segments}} \bar{g}_{\mathrm{Na_V}1.2}}{\sum_{\text{AIS segments}} \bar{g}_{\mathrm{Na_V}1.2}+\bar{g}_{\mathrm{Na_V}1.6}}\right)$. In the leftmost plot, each data point is marked by its x–value. All curves converge to a single point at 0.5 on the abscissa. That point is the I_{BP} for the flat distribution ($x = 0$), where half of the $\mathrm{Na_V}$ channels in the AIS are $\mathrm{Na_V}$1.2 and the other half are $\mathrm{Na_V}$1.6, and κ has no effect. So, $x = 0$ at the point where the curves converge, and x increases radially outward from this point, with all curves terminating at $x = 1$. The same pattern holds in the rightmost plot, in which the original markers were kept for comparison with Fig 2. These plots demonstrate that the impact on I_{BP} of spatially separating the $\mathrm{Na_V}$ subtypes (by increasing x) cannot be reduced to the concomitant change in total $\mathrm{Na_V}$1.2 (or $\mathrm{Na_V}$1.6) conductance —which only occurs when $\kappa \neq 0.5$. If the proportion of $\mathrm{Na_V}$ conductance contributed by each subtype in the AIS were really what determines the threshold, then every black marker would land at the same point. However, varying the spatial separation of $\mathrm{Na_V}$ subtypes in the AIS changes I_{BP} without any change to the ratio of total $\mathrm{Na_V}$1.2 versus $\mathrm{Na_V}$1.6 conductance (i.e. when $\kappa = 0.5$, the ratio is not affected by x).

H Diffusion coefficients

Diffusion coefficients for Na^+, K^+, and Cl^- in water at 25.0 ℃ are provided by [49] :

$$\begin{cases} D_{\mathrm{Na}^+} = 1.334 \times 10^{-9} \frac{\mathrm{m}^2}{\mathrm{s}} \\ D_{\mathrm{K}^+} = 1.957 \times 10^{-9} \frac{\mathrm{m}^2}{\mathrm{s}} \\ D_{\mathrm{Cl}^-} = 2.032 \times 10^{-9} \frac{\mathrm{m}^2}{\mathrm{s}}. \end{cases} \tag{S14}$$

Our simulations are done at a warmer temperature, so these coefficients need to be adjusted. We make the adjustment using the Stokes-Einstein equation

$$D = \frac{k_B T}{\zeta(T)} = \frac{k_B T}{6\pi r \eta(T)} ; \tag{S15}$$

where k_B is Boltzmann's constant, T is the temperature (Kelvins K), and ζ is called the drag coefficient. ζ is given by the ion's radius r and the viscosity $\eta(T)$ of the medium (liquid water), which depends on temperature. Hence the ratio of diffusion coefficients for ion species Z at T_2 and T_1 is

$$\frac{D_{\mathrm{Z}}(T_2)}{D_{\mathrm{Z}}(T_1)} = \left(\frac{T_2}{T_1}\right)\left(\frac{\eta(T_1)}{\eta(T_2)}\right), \tag{S16}$$

with T_2 and T_1 converted to K. The reference values provided by [49] are measured at 298.15K (25.0℃), and the simulation temperature is 310.15K (37.0℃). Assuming the viscosity of water, we have $\eta(T_1) \cong 0.89$ mPa s and $\eta(T_2) \cong 0.691$ mPa s. Substituting these η's into Equation S16 gives for each ionic species Z

$$\underbrace{D_{\mathrm{Z}}(310.15\mathrm{K})}_{37.0℃} \cong 1.34 \times \underbrace{D_{\mathrm{Z}}(298.15\mathrm{K})}_{25.0℃}, \tag{S17}$$

which yields the temperature-adjusted diffusion coefficients

$$\begin{cases} D_{\mathrm{Na}^+} = 1.79 \times 10^{-9} \frac{\mathrm{m}^2}{\mathrm{s}} \\ D_{\mathrm{K}^+} = 2.62 \times 10^{-9} \frac{\mathrm{m}^2}{\mathrm{s}} \\ D_{\mathrm{Cl}^-} = 2.72 \times 10^{-9} \frac{\mathrm{m}^2}{\mathrm{s}}. \end{cases} \tag{S18}$$

I Tables of parameters

In Table A, we compare our Hu-based model from the main text to previous models on which it is based ([12, 26, 15]). At different locations in the cell, we tabularize parameter values from those papers alongside our own. We also include certain measurements (membrane potential, ion concentrations, Nernst potentials) taken from our model at each location once the cell has equilibrated.

The following symbols are used in Table A: (Units are given in the rightmost column.)

"×" This symbol appears when a parameter is not featured in the model corresponding to a given column.

$\boldsymbol{V_{\mathrm{rest}}}$ Resting potential. The transmembrane voltage of the model neuron at steady-state with no injected current.

$\boldsymbol{V_{\mathrm{m}}^{\mathrm{location}}}$ Transmembrane voltage at 'location'. In our model, the steady-state transmembrane voltage is actively maintained everywhere by Na^+/K^+-pumps and longitudinal diffusion, which control the explicit intracellular and extracellular ion concentrations. As such, the tabulated values are *recorded* from the model, not parameters per se. In the other models listed, V_{m} is identical to V_{rest}.

$\boldsymbol{R_{\mathrm{axial}}}$ Axial resistance. The resistance to axial current flow across a compartment is R_{axial} multiplied by the compartment length, divided by the compartment's cross-sectional area.

$\boldsymbol{D_{\mathrm{ion}}}$ Diffusion coefficient of the specified ion in water at $37℃$.

$\boldsymbol{T_{\mathrm{ref}}}$ Reference temperature of experimentally developed channel properties used in the models. Past modelling, on which this paper is based, used temperature factors (described below) to adjust channel densities and speed up channel kinetics to warmer temperatures.

T Simulation temperature.

$\boldsymbol{Q_{\bar{g}_{\mathrm{V}}}^{\mathrm{Mainen}} = Q_{10,\bar{g}_{\mathrm{V}}}^{(T-T_{\mathrm{ref}})/10℃}}$ To adjust for a warmer simulation temperature of $T = 37℃$ [26] scales up the maximal voltage-gated conductances, which were originally developed at $T = 23℃$ ([12]), by a factor which we denote $Q_{\mathrm{Mainen}}^{\bar{g}_{\mathrm{V}}}$. The authors warn in their model code that this scaling is valid only at $37℃$ and state that their program is not designed to model other temperatures. This internal temperature scaling can accidentally obscure parameter settings when one attempts to borrow parameters separately from the past code or from later papers that reuse these channel models. For this reason, we

have removed the temperature scaling from our own model code by setting our reference temperature equal to the simulation temperature, which sets $Q^{\bar{g}_\mathrm{V}}_\mathrm{Mainen} = 1$.

$\bar{g}^{\mathbf{location}}_{\mathbf{Na_V \cdot Total}}$ Total combined maximal voltage-gated conductance density of $\mathrm{Na_V}$1.2 and $\mathrm{Na_V}$1.6 at the specified 'location' in the cell (see Equation S2). The location can be an entire Section if the membrane properties are uniform (in NEURON, Sections consist of multiple compartments). For example, this model has uniform somatodendritic channel densities, so 'soma' is sufficient to specify those parameters.

$\bar{g}^{\mathbf{location}}_{\mathbf{K_V \cdot Total}}$ Maximal voltage-gated K^+ conductance density at the specified 'location'.

$C_\mathbf{m}$ Membrane capacitance per unit area. Applies everywhere except at internodes.

$C^{\mathbf{myelin}}_\mathbf{m}$ Membrane capacitance of myelinated internodes.

$L_\mathbf{Section}$ Length of the specified 'Section' (soma, hillock, AIS, etc.)

$d^{\mathbf{position\ within\ Section}}_{\mathbf{Section}}$ Diameter at a given normalized position $(0 \leqslant s \leqslant 1)$ within the specified Section (soma, hillock, AIS, etc.). The position is not specified when the diameter is uniform.

$R^{\mathbf{Section}}_{\mathbf{Passive}}$ Membrane resistivity in 'Section'.

$g^{\mathbf{Section}}_{\mathbf{Passive}}$ Passive generic transmembrane leak conductance density in 'Section'.

$g^{\mathbf{Section}}_{\mathbf{Ion,\ leak}}$ Specific leak conductance density of 'Ion' in 'Section'.

$I^{\mathbf{Section}}_{\mathbf{MaxPump}}$ Maximum Na^+/K^+-pump current density in 'Section'.

$E^{\mathbf{Section}}_{\mathbf{current\ type}}$ Nernst Potential (reversal potential) of transmembrane current 'current type' (e.g. 'leak') in 'Section' (e.g. 'soma').

$[\mathbf{Ion}]^{\mathbf{Section}}_{\mathbf{in/out}}$ Intracellular ('in') or Extracellular ('out') concentration of 'Ion' at 'Section', with the neuron at steady-state. Because these concentrations are maintained by Na^+/K^+-pumps and longitudinal diffusion in our model, the concentrations tabulated here are measurements *recorded* from the simulation at steady-state, rather than being fixed parameters.

ranvier0, myelin0: In our model, there are 15 nodes of Ranvier and 16 myelinated internodes. The Section named 'ranvier0' is the first node of Ranvier. And 'myelin0' is the first myelinated internode of the axon, located between the distal end of the AIS (or the distal end of the bare axon when that is included) and the proximal end of ranvier0.

dend11[**22**] Main apical dendrite.

Table A: Parameters of the Hu-Based model from the main text, compared across the Hu model [15] and the original Mainen models [12, 26] upon which the Hu model was built. In the soma and dendrites of the Hu-based model, channel densities differ significantly from Hu et al. (2009) [15] to attenuate the backpropagated action potential. In the Supporting "modified Hu-based model" (Section D), those conductances match [15] closely. (We have opted to call the version in the main text the "Hu-based model" (i.e. with attenuating BAP), and the version in the Supporting Information the "modified Hu-based model" even though the parameters of the latter more closely match those of [15].) In the latter, BAP attenuation is negligible, however, the impacts on I_{BP} of varying the AIS Na_V distribution are qualitatively unchanged from the main text.

Type	Parameter Name	Hu-Based	Mainen95	Mainen96	Hu2009	units
Global						
	V_{rest}	-70.0	-70.0	-70.0	-70.0	mV
	R_{axial}	150	200	150	150	$\Omega \cdot \mathrm{cm}$
	D_{Cl^-}	2.72	×	×	×	$10^{-9}\frac{\mathrm{m}^2}{\mathrm{s}}$
	D_{Na^+}	1.79	×	×	×	$10^{-9}\frac{\mathrm{m}^2}{\mathrm{s}}$
	D_{K^+}	2.62	×	×	×	$10^{-9}\frac{\mathrm{m}^2}{\mathrm{s}}$
	T_{ref}	37.0	23.0	23.0	23.0	°C
	T	37.0	23.0	37.0	37.0	°C
	$Q_{\bar{g}_{\mathrm{V}}}^{\mathrm{Mainen}} = Q_{10,\bar{g}_{\mathrm{V}}}^{(T-T_{\mathrm{ref}})/10°\mathrm{C}}$	1.0	1.0	3.21	3.21	dimensionless
	C_{m}	0.75	0.75	0.75	1.0	$\frac{\mu F}{\mathrm{cm}^2}$

section	Parameter Name	Hu-Based	Mainen95	Mainen96	Hu2009	units
	$C_{\text{m}}^{\text{myelin}}$	0.02	0.04	0.02	0.02	$\frac{\mu F}{\text{cm}^2}$
Geometry						
	L_{soma}	30.0	25.8	30.0	30.0	μm
	L_{hillock}	10.0	10.0	10.0	10.0	μm
	L_{AIS}	25.0	15.0	15.0	50.0	μm
	$L_{\text{bare·axon}}$	400	×	×	400	μm
	$d_{\text{soma}}^{0.5}$	19.38	15.2	19.0	19.0	μm
	$d_{\text{soma}}^{1.0}$	8.62	15.2	8.6	8.6	μm
	$d_{\text{hillock}}^{0.0}$	8.62	4.0	4.0	3.8	μm
	$d_{\text{hillock}}^{1.0}$	1.22	1.0	1.0	2.4	μm
	d_{AIS}	1.22	1.0	1.0	1.22	μm
	$d_{\text{bare·axon}}$	1.22	×	×	1.02	μm

section	Parameter Name	Hu-Based	Mainen95	Mainen96	Hu2009	units
soma						
	$\bar{g}_{\text{Na}_\text{V}\cdot\text{Total}}^{\text{soma}}$	7.53×10^{-2}	0.003	0.002	0.008	$\frac{\mho}{\text{cm}^2}$

$\bar{g}^{\text{soma}}_{\text{Na}_\text{V}\cdot\text{Total}} \times Q^{\text{Mainen}}_{\bar{g}_\text{V}}$	7.53×10^{-2}	3.00×10^{-3}	6.42×10^{-3}	2.57×10^{-2}	$\frac{\mho}{\text{cm}^2}$
$\bar{g}^{\text{soma}}_{\text{K}_\text{V}}$	2.26×10^{-2}	0.01	0.02	0.002	$\frac{\mho}{\text{cm}^2}$
$\bar{g}^{\text{soma}}_{\text{K}_\text{V}} \times Q^{\text{Mainen}}_{\bar{g}_\text{V}}$	2.26×10^{-2}	1.00×10^{-2}	6.42×10^{-2}	6.42×10^{-3}	$\frac{\mho}{\text{cm}^2}$
$R^{\text{soma}}_{\text{Passive}}$	2.39×10^{3}	4.00×10^{4}	3.00×10^{4}	3.00×10^{4}	$\Omega \cdot \text{cm}^2$
$g^{\text{soma}}_{\text{Passive}}$	4.19×10^{-4}	2.50×10^{-5}	3.33×10^{-5}	3.33×10^{-5}	$\frac{\mho}{\text{cm}^2}$
$g^{\text{soma}}_{\text{Cl}^-\text{, leak}}$	6.27×10^{-5}	×	×	×	$\frac{\mho}{\text{cm}^2}$
$g^{\text{soma}}_{\text{Na}^+\text{, leak}}$	6.68×10^{-5}	×	×	×	$\frac{\mho}{\text{cm}^2}$
$g^{\text{soma}}_{\text{K}^+\text{, leak}}$	2.90×10^{-4}	×	×	×	$\frac{\mho}{\text{cm}^2}$
$I^{\text{soma}}_{\text{MaxPump}}$	3.99×10^{-2}	×	×	×	$\frac{\text{mA}}{\text{cm}^2}$
$V^{\text{soma}}_{\text{m}}$	−70.0	−70.0	−70.0	−70.0	mV
$E^{\text{soma}}_{\text{leak}}$	×	−70.0	−70.0	−70.0	mV
$E^{\text{soma}}_{\text{Cl}}$	−70.0	×	×	×	mV
$E^{\text{soma}}_{\text{Na}}$	60.0	60.0	50.0	60.0	mV
$E^{\text{soma}}_{\text{K}}$	−90.0	−90.0	−90.0	−90.0	mV

	$[\mathrm{Cl}^-]_{\mathrm{in}}^{\mathrm{soma}}$	11.0	×	×	×	mM
	$[\mathrm{Cl}^-]_{\mathrm{out}}^{\mathrm{soma}}$	151.0	×	×	×	mM
	$[\mathrm{Na}^+]_{\mathrm{in}}^{\mathrm{soma}}$	15.0	×	×	×	mM
	$[\mathrm{Na}^+]_{\mathrm{out}}^{\mathrm{soma}}$	141.6	×	×	×	mM
	$[\mathrm{K}^+]_{\mathrm{in}}^{\mathrm{soma}}$	140.0	×	×	×	mM
	$[\mathrm{K}^+]_{\mathrm{out}}^{\mathrm{soma}}$	4.8	×	×	×	mM

section	Parameter Name	Hu-Based	Mainen95	Mainen96	Hu2009	units
AIS						
	$\bar{g}_{\mathrm{Na_V}\cdot\mathrm{Total}}^{\mathrm{AIS}}$	1.505	3.0	3.0	0.32	$\frac{℧}{\mathrm{cm}^2}$
	$\bar{g}_{\mathrm{Na_V}\cdot\mathrm{Total}}^{\mathrm{AIS}} \times Q_{\bar{g}_\mathrm{V}}^{\mathrm{Mainen}}$	1.505	3.0	9.628	1.027	$\frac{℧}{\mathrm{cm}^2}$
	$\bar{g}_{\mathrm{K_V}}^{\mathrm{AIS}}$	4.52×10^{-1}	×	0.2	0.1	$\frac{℧}{\mathrm{cm}^2}$
	$\bar{g}_{\mathrm{K_V}}^{\mathrm{AIS}} \times Q_{\bar{g}_\mathrm{V}}^{\mathrm{Mainen}}$	4.52×10^{-1}	×	6.42×10^{-1}	3.21×10^{-1}	$\frac{℧}{\mathrm{cm}^2}$
	$R_{\mathrm{Passive}}^{\mathrm{AIS}}$	4.77×10^{1}	5.00×10^{1}	5.00×10^{1}	5.00×10^{1}	$\Omega \cdot \mathrm{cm}^2$
	$g_{\mathrm{Passive}}^{\mathrm{AIS}}$	2.10×10^{-2}	2.00×10^{-2}	2.00×10^{-2}	2.00×10^{-2}	$\frac{℧}{\mathrm{cm}^2}$
	$g_{\mathrm{Cl}^-,\ \mathrm{leak}}^{\mathrm{AIS}}$	3.14×10^{-3}	×	×	×	$\frac{℧}{\mathrm{cm}^2}$

$g_{\mathrm{Na^+, leak}}^{\mathrm{AIS}}$	3.34×10^{-3}	×	×	×	$\frac{℧}{\mathrm{cm^2}}$
$g_{\mathrm{K^+, leak}}^{\mathrm{AIS}}$	1.45×10^{-2}	×	×	×	$\frac{℧}{\mathrm{cm^2}}$
$I_{\mathrm{MaxPump}}^{\mathrm{AIS}}$	1.995	×	×	×	$\frac{\mathrm{mA}}{\mathrm{cm^2}}$
$V_{\mathrm{m}}^{\mathrm{AIS}}$	−69.9	−70.0	−70.0	−70.0	mV
$E_{\mathrm{leak}}^{\mathrm{AIS}}$	×	−70.0	−70.0	−70.0	mV
$E_{\mathrm{Cl}}^{\mathrm{AIS}}$	−70.0	×	×	×	mV
$E_{\mathrm{Na}}^{\mathrm{AIS}}$	59.9	60.0	50.0	60.0	mV
$E_{\mathrm{K}}^{\mathrm{AIS}}$	−90.0	−90.0	−90.0	−90.0	mV
$[\mathrm{Cl^-}]_{\mathrm{in}}^{\mathrm{AIS}}$	11.0	×	×	×	mM
$[\mathrm{Cl^-}]_{\mathrm{out}}^{\mathrm{AIS}}$	151.0	×	×	×	mM
$[\mathrm{Na^+}]_{\mathrm{in}}^{\mathrm{AIS}}$	15.1	×	×	×	mM
$[\mathrm{Na^+}]_{\mathrm{out}}^{\mathrm{AIS}}$	141.6	×	×	×	mM
$[\mathrm{K^+}]_{\mathrm{in}}^{\mathrm{AIS}}$	139.9	×	×	×	mM
$[\mathrm{K^+}]_{\mathrm{out}}^{\mathrm{AIS}}$	4.8	×	×	×	mM

section	Parameter Name	Hu-Based	Mainen95	Mainen96	Hu2009	units
myelin0						
	$\bar{g}^{\text{myelin0}}_{\text{Na}_\text{V}\cdot\text{Total}}$	1.61×10^{-2}	0.003	×	0.002	$\frac{\mho}{\text{cm}^2}$
	$\bar{g}^{\text{myelin0}}_{\text{Na}_\text{V}\cdot\text{Total}} \times Q^{\text{Mainen}}_{\bar{g}_\text{V}}$	1.61×10^{-2}	3.00×10^{-3}	×	6.42×10^{-3}	$\frac{\mho}{\text{cm}^2}$
	$\bar{g}^{\text{myelin0}}_{\text{K}_\text{V}}$	4.82×10^{-3}	×	×	×	$\frac{\mho}{\text{cm}^2}$
	$\bar{g}^{\text{myelin0}}_{\text{K}_\text{V}} \times Q^{\text{Mainen}}_{\bar{g}_\text{V}}$	4.82×10^{-3}	×	×	×	$\frac{\mho}{\text{cm}^2}$
	$R^{\text{myelin0}}_{\text{Passive}}$	1.12×10^{4}	4.00×10^{4}	3.00×10^{4}	3.00×10^{4}	$\Omega \cdot \text{cm}^2$
	$g^{\text{myelin0}}_{\text{Passive}}$	8.94×10^{-5}	2.50×10^{-5}	3.33×10^{-5}	3.33×10^{-5}	$\frac{\mho}{\text{cm}^2}$
	$g^{\text{myelin0}}_{\text{Cl}^-\text{, leak}}$	1.34×10^{-5}	×	×	×	$\frac{\mho}{\text{cm}^2}$
	$g^{\text{myelin0}}_{\text{Na}^+\text{, leak}}$	1.43×10^{-5}	×	×	×	$\frac{\mho}{\text{cm}^2}$
	$g^{\text{myelin0}}_{\text{K}^+\text{, leak}}$	6.18×10^{-5}	×	×	×	$\frac{\mho}{\text{cm}^2}$
	$I^{\text{myelin0}}_{\text{MaxPump}}$	8.51×10^{-3}	×	×	×	$\frac{\text{mA}}{\text{cm}^2}$
	$V^{\text{myelin0}}_{\text{m}}$	−69.5	−70.0	−70.0	−70.0	mV
	$E^{\text{myelin0}}_{\text{leak}}$	×	−70.0	−70.0	−70.0	mV
	$E^{\text{myelin0}}_{\text{Cl}}$	−70.0	×	×	×	mV

section	Parameter Name	Hu-Based	Mainen95	Mainen96	Hu2009	units
	$E_{\mathrm{Na}}^{\mathrm{myelin0}}$	60.0	60.0	50.0	60.0	mV
	$E_{\mathrm{K}}^{\mathrm{myelin0}}$	−90.0	−90.0	−90.0	−90.0	mV
	$[\mathrm{Cl}^-]_{\mathrm{in}}^{\mathrm{myelin0}}$	11.0	×	×	×	mM
	$[\mathrm{Cl}^-]_{\mathrm{out}}^{\mathrm{myelin0}}$	151.0	×	×	×	mM
	$[\mathrm{Na}^+]_{\mathrm{in}}^{\mathrm{myelin0}}$	15.0	×	×	×	mM
	$[\mathrm{Na}^+]_{\mathrm{out}}^{\mathrm{myelin0}}$	141.6	×	×	×	mM
	$[\mathrm{K}^+]_{\mathrm{in}}^{\mathrm{myelin0}}$	140.0	×	×	×	mM
	$[\mathrm{K}^+]_{\mathrm{out}}^{\mathrm{myelin0}}$	4.8	×	×	×	mM

section	Parameter Name	Hu-Based	Mainen95	Mainen96	Hu2009	units
ranvier0						
	$\bar{g}_{\mathrm{Na_V \cdot Total}}^{\mathrm{ranvier0}}$	8.03×10^{-1}	3.0	3.0	0.16	$\frac{\mho}{\mathrm{cm}^2}$
	$\bar{g}_{\mathrm{Na_V \cdot Total}}^{\mathrm{ranvier0}} \times Q_{\bar{g}_{\mathrm{V}}}^{\mathrm{Mainen}}$	8.03×10^{-1}	3.0	9.628	5.13×10^{-1}	$\frac{\mho}{\mathrm{cm}^2}$
	$\bar{g}_{\mathrm{K_V}}^{\mathrm{ranvier0}}$	2.41×10^{-1}	×	×	×	$\frac{\mho}{\mathrm{cm}^2}$
	$\bar{g}_{\mathrm{K_V}}^{\mathrm{ranvier0}} \times Q_{\bar{g}_{\mathrm{V}}}^{\mathrm{Mainen}}$	2.41×10^{-1}	×	×	×	$\frac{\mho}{\mathrm{cm}^2}$
	$R_{\mathrm{Passive}}^{\mathrm{ranvier0}}$	2.24×10^{2}	5.00×10^{1}	5.00×10^{1}	5.00×10^{1}	$\Omega \cdot \mathrm{cm}^2$

$g_{\text{Passive}}^{\text{ranvier0}}$	4.47×10^{-3}	2.00×10^{-2}	2.00×10^{-2}	2.00×10^{-2}	$\frac{\mho}{\text{cm}^2}$
$g_{\text{Cl}^-\text{, leak}}^{\text{ranvier0}}$	6.69×10^{-4}	×	×	×	$\frac{\mho}{\text{cm}^2}$
$g_{\text{Na}^+\text{, leak}}^{\text{ranvier0}}$	7.13×10^{-4}	×	×	×	$\frac{\mho}{\text{cm}^2}$
$g_{\text{K}^+\text{, leak}}^{\text{ranvier0}}$	3.09×10^{-3}	×	×	×	$\frac{\mho}{\text{cm}^2}$
$I_{\text{MaxPump}}^{\text{ranvier0}}$	4.26×10^{-1}	×	×	×	$\frac{\text{mA}}{\text{cm}^2}$
$V_{\text{m}}^{\text{ranvier0}}$	−69.4	−70.0	−70.0	−70.0	mV
$E_{\text{leak}}^{\text{ranvier0}}$	×	−70.0	−70.0	−70.0	mV
$E_{\text{Cl}}^{\text{ranvier0}}$	−70.0	×	×	×	mV
$E_{\text{Na}}^{\text{ranvier0}}$	60.0	60.0	50.0	60.0	mV
$E_{\text{K}}^{\text{ranvier0}}$	−90.0	−90.0	−90.0	−90.0	mV
$[\text{Cl}^-]_{\text{in}}^{\text{ranvier0}}$	11.0	×	×	×	mM
$[\text{Cl}^-]_{\text{out}}^{\text{ranvier0}}$	151.0	×	×	×	mM
$[\text{Na}^+]_{\text{in}}^{\text{ranvier0}}$	15.0	×	×	×	mM
$[\text{Na}^+]_{\text{out}}^{\text{ranvier0}}$	141.6	×	×	×	mM

section	Parameter Name	HU-BASED	MAINEN95	MAINEN96	HU2009	UNITS
	$[K^+]_{in}^{ranvier0}$	140.0	×	×	×	mM
	$[K^+]_{out}^{ranvier0}$	4.8	×	×	×	mM

section	Parameter Name	HU-BASED	MAINEN95	MAINEN96	HU2009	UNITS
dend11[22]						
	$\bar{g}_{Na_V \cdot Total}^{dend11[22]}$	2.51×10^{-3}	0.003	0.00015	8.0×10^{-3}	$\frac{℧}{cm^2}$
	$\bar{g}_{Na_V \cdot Total}^{dend11[22]} \times Q_{\bar{g}_V}^{Mainen}$	2.51×10^{-3}	3.00×10^{-3}	4.82×10^{-3}	2.57×10^{-2}	$\frac{℧}{cm^2}$
	$\bar{g}_{K_V}^{dend11[22]}$	7.53×10^{-4}	0.01	×	1.0×10^{-3}	$\frac{℧}{cm^2}$
	$\bar{g}_{K_V}^{dend11[22]} \times Q_{\bar{g}_V}^{Mainen}$	7.53×10^{-4}	1.00×10^{-2}	×	3.21×10^{-3}	$\frac{℧}{cm^2}$
	$R_{Passive}^{dend11[22]}$	7.16×10^{4}	4.00×10^{4}	3.00×10^{4}	3.00×10^{4}	$\Omega \cdot cm^2$
	$g_{Passive}^{dend11[22]}$	1.40×10^{-5}	2.50×10^{-5}	3.33×10^{-5}	3.33×10^{-5}	$\frac{℧}{cm^2}$
	$g_{Cl^-, leak}^{dend11[22]}$	2.09×10^{-6}	×	×	×	$\frac{℧}{cm^2}$
	$g_{Na^+, leak}^{dend11[22]}$	2.23×10^{-6}	×	×	×	$\frac{℧}{cm^2}$
	$g_{K^+, leak}^{dend11[22]}$	9.66×10^{-6}	×	×	×	$\frac{℧}{cm^2}$
	$I_{MaxPump}^{dend11[22]}$	1.33×10^{-3}	×	×	×	$\frac{mA}{cm^2}$
	$V_m^{dend11[22]}$	−70.0	−70.0	−70.0	−70.0	mV

$E_{\mathrm{leak}}^{\mathrm{dend11[22]}}$	×	−70.0	−70.0	−70.0	mV
$E_{\mathrm{Cl}}^{\mathrm{dend11[22]}}$	−70.0	×	×	×	mV
$E_{\mathrm{Na}}^{\mathrm{dend11[22]}}$	60.0	60.0	50.0	60.0	mV
$E_{\mathrm{K}}^{\mathrm{dend11[22]}}$	−90.0	−90.0	−90.0	−90.0	mV
$[\mathrm{Cl}^-]_{\mathrm{in}}^{\mathrm{dend11[22]}}$	11.0	×	×	×	mM
$[\mathrm{Cl}^-]_{\mathrm{out}}^{\mathrm{dend11[22]}}$	151.0	×	×	×	mM
$[\mathrm{Na}^+]_{\mathrm{in}}^{\mathrm{dend11[22]}}$	15.0	×	×	×	mM
$[\mathrm{Na}^+]_{\mathrm{out}}^{\mathrm{dend11[22]}}$	141.6	×	×	×	mM
$[\mathrm{K}^+]_{\mathrm{in}}^{\mathrm{dend11[22]}}$	140.0	×	×	×	mM
$[\mathrm{K}^+]_{\mathrm{out}}^{\mathrm{dend11[22]}}$	4.8	×	×	×	mM
